# Multi‐Physical Lattice Metamaterials Enabled by Additive Manufacturing: Design Principles, Interaction Mechanisms, and Multifunctional Applications

**DOI:** 10.1002/advs.202405835

**Published:** 2025-01-20

**Authors:** Winston Wai Shing Ma, Hang Yang, Yijing Zhao, Xinwei Li, Junhao Ding, Shuo Qu, Quyang Liu, Zongxin Hu, Rui Li, Quanqing Tao, Haoming Mo, Wei Zhai, Xu Song

**Affiliations:** ^1^ Department of Mechanical and Automation Engineering Chinese University of Hong Kong Sha Tin Hong Kong 999077 China; ^2^ Department of Mechanical Engineering National University of Singapore Singapore 117575 Singapore; ^3^ Faculty of Science, Agriculture, and Engineering Newcastle University Singapore 567739 Singapore

**Keywords:** multi‐physical lattice metamaterials, additive manufacturing, structure‐mechanism‐property relationships, interaction mechanisms, multifunctional applications

## Abstract

Lattice metamaterials emerge as advanced architected materials with superior physical properties and significant potential for lightweight applications. Recent developments in additive manufacturing (AM) techniques facilitate the manufacturing of lattice metamaterials with intricate microarchitectures and promote their applications in multi‐physical scenarios. Previous reviews on lattice metamaterials have largely focused on a specific/single physical field, with limited discussion on their multi‐physical properties, interaction mechanisms, and multifunctional applications. Accordingly, this article critically reviews the design principles, structure‐mechanism‐property relationships, interaction mechanisms, and multifunctional applications of multi‐physical lattice metamaterials enabled by AM techniques. First, lattice metamaterials are categorized into homogeneous lattices, inhomogeneous lattices, and other forms, whose design principles and AM processes are critically discussed, including the benefits and drawbacks of different AM techniques for fabricating different types of lattices. Subsequently, the structure–mechanism–property relationships and interaction mechanisms of lattice metamaterials in a range of physical fields, including mechanical, acoustic, electromagnetic/optical, and thermal disciplines, are summarized to reveal critical design principles. Moreover, the multifunctional applications of lattice metamaterials, such as sound absorbers, insulators, and manipulators, sensors, actuators, and soft robots, thermal management, invisible cloaks, and biomedical implants, are enumerated. These design principles and structure‐mechanism‐property relationships provide effective design guidelines for lattice metamaterials in multifunctional applications.

## Graphical abstract

This article critically reviews the structure–mechanism–property relationships and interaction mechanisms of lattice metamaterials enabled by additive manufacturing in a range of physical (mechanical, acoustic, electromagnetic/optical, and thermal) fields. Accordingly, critical design principles are summarized to achieve simultaneous enhancement of their multi‐physical properties. These design principles and structure–mechanism–property relationships deliver effective design guidelines for the multifunctional applications of multi‐physical lattice metamaterials.

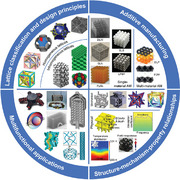

## 1 Introduction

Metamaterials are architected materials with special properties governed by internal microarchitectures instead of chemical compositions.^[^ ^1^, ^2^, ^3^ ^]^ Through appropriate design of internal microarchitectures, metamaterials can be strategically engineered to realize a range of unconventional physical properties.^[^ ^1^, ^2^, ^3^ ^]^ As a distinctive subclass of metamaterials, lattice metamaterials comprise periodic arrays of unit cells and exhibit significant potential for lightweight structural applications.^[^ ^4^, ^5^ ^]^ Depending on the spatial distribution patterns of microarchitectures, lattice metamaterials are divided into homogeneous lattices, inhomogeneous lattices, and other forms.^[^ ^5^ ^]^ Typically, homogeneous lattices are classified based on the structural geometries/configurations of elementary units as truss,^[^ ^6^, ^7^, ^8^, ^9^ ^]^ plate,^[^ ^10^, ^11^, ^12^ ^]^ shell,^[^ ^13^, ^14^, ^15^ ^]^ hybrid,^[^ ^16^, ^17^, ^18^ ^]^ and hierarchical lattices.^[^ ^19^, ^20^ ^]^ Besides, inhomogeneous lattices are classified based on the architected patterns of microarchitectures as graded^[^ ^21^, ^22^ ^]^ and heterogeneous^[^ ^23^, ^24^, ^25^, ^26^ ^]^ lattices. Lattice metamaterials generally possess intricate microarchitectures, which are difficult to fabricate via processes that employ traditional manufacturing techniques,^[^ ^27^, ^28^ ^]^ such as casting, forging, welding, cutting, folding, extrusion molding, etc. Recent developments in additive manufacturing (AM) techniques have circumvented these difficulties and facilitated the manufacture of lattice metamaterials with intricate microarchitectures significantly.^[^ ^12^, ^15^, ^29^ ^]^

To date, many researchers have explored the design of novel lattice metamaterials with superior mechanical/physical properties and investigated their applications in different industries.^[^ ^30^, ^31^ ^]^ The underlying relationships between the structural geometries/configurations and mechanical behaviors of lattice metamaterials have been comprehensively investigated to achieve a broad range of unconventional properties, including exceptional mechanical properties, non‐positive equivalent parameters, anomalous deformation mechanisms, amongst many other interesting phenomena. Specifically, exceptional mechanical properties encompass ultra‐lightweight, ultra‐stiff, ultra‐strong,^[^ ^32^, ^33^, ^34^ ^]^ (an)isotropic,^[^ ^10^, ^11^, ^12^ ^]^ pentamode properties,^[^ ^35^, ^36^, ^37^ ^]^ etc. Non‐positive equivalent parameters are associated with non‐positive Poisson's ratios,^[^ ^38^, ^39^, ^40^ ^]^ non‐positive stiffnesses and non‐positive incremental stiffnesses,^[^ ^41^, ^42^ ^]^ non‐positive coefficients of thermal expansion (CTEs),^[^ ^43^, ^44^ ^]^ etc. Anomalous deformation mechanisms involve phenomena like torsion under axial tensile/compressive loads,^[^ ^45^, ^46^ ^]^ lateral expansion/contraction, and longitudinal extension/shrinkage under torsional loads,^[^ ^47^, ^48^ ^]^ non‐reciprocity,^[^ ^38^ ^]^ etc. Lattice metamaterials have also been devised to exhibit numerous unconventional physical properties, including acoustic,^[^ ^49^, ^50^ ^]^ electromagnetic (EM)/optical,^[^ ^51^, ^52^ ^]^ thermal,^[^ ^53^, ^54^, ^55^, ^56^ ^]^ and other physical properties.^[^ ^57^, ^58^, ^59^ ^]^ Therefore, lattice metamaterials exhibit significant potential for multifunctional applications in a wide range of areas, including sound absorbers, insulators, and manipulators,^[^ ^17^, ^49^, ^60^ ^]^ sensors, actuators, and soft robots,^[^ ^61^, ^62^ ^]^ thermal management,^[^ ^63^, ^64^ ^]^ invisible cloaks,^[^ ^65^, ^66^ ^]^ biomedical implants,^[^ ^67^, ^68^ ^]^ and others.^[^ ^69^, ^70^, ^71^ ^]^ These applications involve the coupling of two or more independent properties, such as mechanical property and another physical property at least. Typically, there are specific functional requirements for lattice microarchitectures in specific applications, which means that an appropriate design is necessary. For instance, open‐cell channels are required for mass transport in biomedical implants, and their mechanical properties should be matched with natural bones to prevent stress shielding or load redistribution.^[^ ^72^, ^73^ ^]^ Accordingly, the underlying mechanisms governing interactions between different mechanical/physical properties should be determined to enable the development of rules for devising lattice metamaterials with desired multi‐physical properties. However, previous reviews on lattice metamaterials are either based on a specific type of constitutive materials,^[^ ^74^, ^75^ ^]^ or primarily focus on a specific form of internal microarchitectures,^[^ ^76^, ^77^, ^78^, ^79^ ^]^ or mainly discuss their AM procedures to realize high quality and fidelity,^[^ ^80^, ^81^ ^]^ or predominantly address the design rules to enhance their properties within a specific/single physical field,^[^ ^82^, ^83^, ^84^, ^85^, ^86^, ^87^, ^88^, ^89^ ^]^ with limited discussion on their multi‐physical properties, interaction mechanisms, and multifunctional applications.

Although recent advances in AM techniques have significantly facilitated the manufacture and application of lattice metamaterials with intricate microarchitectures,^[^ ^12^, ^15^, ^29^ ^]^ different AM techniques exhibit different advantages and disadvantages when applied to different types of lattice metamaterials. Typically, the fabrication of truss lattices via layer‐by‐layer AM processes, such as laser powder bed fusion (LPBF),^[^ ^90^, ^91^ ^]^ stereolithography (SLA),^[^ ^92^ ^]^ and fused deposition modeling (FDM),^[^ ^93^ ^]^ is limited by overhang constraints. These constraints tend to result in large dimensional variations and surface roughnesses in constituent bars with small overhang angles.^[^ ^90^, ^91^ ^]^ To overcome overhang constraints, truss lattices need to be properly rotated during these AM processes. Alternatively, direct laser writing (DLW) procedure allows laser photons to penetrate deeply into constitutive materials and convert the liquid into a solid within the focal volume.^[^ ^94^ ^]^ As a result, DLW offers a method for accumulating materials that differs significantly from the layer‐by‐layer manner, thereby directly addressing inherent limitations associated with overhang constraints^[^ ^94^ ^]^ and guaranteeing the fidelity of samples.^[^ ^95^ ^]^ Additionally, plate lattices generally consist of a closed‐cell network of plates, which complicate their fabrication via most AM techniques, such as LPBF, SLA, and DLW, since additional holes need to be introduced for the removal of unmolten metal powders or residual resins within the closed‐cell internal cavities.^[^ ^11^, ^96^ ^]^ In contrast, FDM process constructs an object through the gradual deposition of a fine filament of polymers via a programmable print head and exhibits more exceptional additive manufacturability for the fabrication of plate lattices, as no additional holes are required.^[^ ^97^ ^]^ Moreover, shell lattices generally consist of open‐cell, non‐intersecting, smooth, and periodic thin shells, and exhibit more exceptional additive manufacturability than truss and plate lattices. The open‐cell feature enables the direct removal of unmolten metal powders or residual resins produced in metal powder or liquid resin‐based AM processes.^[^ ^15^, ^96^, ^98^ ^]^ Besides, the smoothly connected surfaces of shell lattices automatically overcome overhang constraints and guarantee the high quality and fidelity of samples.^[^ ^90^, ^91^, ^99^ ^]^ The AM processes of other different classes of lattice metamaterials will be discussed in more detail in the main section of this review.

**Figure 1:**
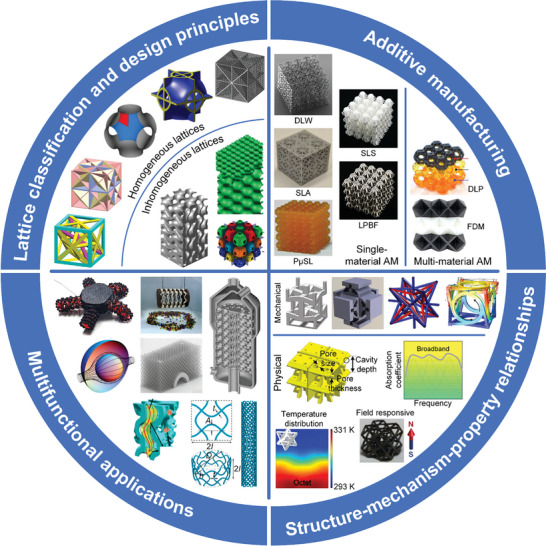
Schematic illustration of the design principles, AM procedures, structure‐mechanism‐property relationships, and multifunctional applications of lattice metamaterials reviewed in this article. Reproduced with permission.^[^ ^8^ ^]^ Copyright 2018, Elsevier. Reproduced with permission.^[^ ^11^ ^]^ Copyright 2018, Wiley. Reproduced with permission.^[^ ^96^ ^]^ Copyright 2021, Elsevier. Reproduced with permission.^[^ ^16^ ^]^ Copyright 2019, Elsevier. Reproduced with permission.^[^ ^170^ ^]^ Copyright 2021, Elsevier. Reproduced with permission.^[^ ^184^ ^]^ Copyright 2022, Wiley. Reproduced with permission.^[^ ^200^ ^]^ Copyright 2018, ACS. Reproduced with permission.^[^ ^208^ ^]^ Copyright 2022, Elsevier. Reproduced with permission.^[^ ^95^ ^]^ Copyright 2020, Elsevier. Reproduced with permission.^[^ ^139^ ^]^ Copyright 2021, Elsevier. Reproduced with permission.^[^ ^15^ ^]^ Copyright 2022, PNAS. Reproduced with permission.^[^ ^157^ ^]^ Copyright 2024, Springer. Reproduced with permission.^[^ ^205^ ^]^ Copyright 2021, AAAS. Reproduced with permission.^[^ ^207^ ^]^ Copyright 2023, Elsevier. Reproduced with permission.^[^ ^288^ ^]^ Copyright 2015, Elsevier. Reproduced with permission.^[^ ^337^ ^]^ Copyright 2018, Wiley. Reproduced with permission.^[^ ^384^ ^]^ Copyright 2018, Elsevier. Reproduced with permission.^[^ ^394^ ^]^ Copyright 2017, AAAS. Reproduced with permission.^[^ ^49^ ^]^ Copyright 2021, Wiley. Reproduced with permission.^[^ ^58^ ^]^ Copyright 2018, AAAS. Reproduced with permission.^[^ ^505^ ^]^ Copyright 2021, Elsevier. Reproduced with permission.^[^ ^581^ ^]^ Copyright 2020, AAAS. Reproduced with permission.^[^ ^323^ ^]^ Copyright 2023, Elsevier. Reproduced with permission.^[^ ^52^ ^]^ Copyright 2006, AAAS. Reproduced with permission.^[^ ^35^ ^]^ Copyright 2014, Springer. Reproduced with permission.^[^ ^643^ ^]^ Copyright 2021, Elsevier. Reproduced with permission.^[^ ^633^ ^]^ Copyright 2022, Elsevier. Reproduced with permission.^[^ ^609^ ^]^ Copyright 2020, nTopology.

In this article, we critically review the design principles, structure‐mechanism‐property relationships, interaction mechanisms, and multifunctional applications of AM techniques‐enabled multi‐physical lattice metamaterials in a variety of forms (**Figure** **1**). Lattice metamaterials that are not enabled by AM techniques, i.e., those that can still be efficiently fabricated in a high‐quality and high‐fidelity manner using traditional manufacturing techniques (including casting, forging, welding, cutting, folding, extrusion molding, etc.), such as two‐dimensional (2D) lattices,^[^ ^100^, ^101^ ^]^ ordered/hierarchical honeycombs,^[^ ^102^, ^103^ ^]^ and kirigami/origami lattices,^[^ ^104^, ^105^, ^106^, ^107^, ^108^ ^]^ have been comprehensively reviewed in many existing literatures and are beyond the scope of this review. More specifically, we mainly address lattice metamaterials enabled by three‐dimensional (3D) printing techniques, and also concisely mention programmable lattice metamaterials enabled by four‐dimensional (4D) printing techniques, i.e., those that will alter their physicochemical properties, such as shape, color, and stiffness, in response to external stimuli, such as sound, electromagnet/light, heat, humidity, and solvent, to achieve tailored multifunctionality. More comprehensive discussions on 4D‐printed programmable lattice metamaterials already exist in many literatures.^[^ ^109^, ^110^, ^111^, ^112^ ^]^ Moreover, we predominantly focus on the enhancement of multi‐physical properties of lattice metamaterials enabled by the design of internal microarchitectures, rather than that enabled by the chemical compositions of constitutive materials. First, the design principles and AM procedures of homogeneous lattices, inhomogeneous lattices, and other forms of lattice metamaterials are reviewed, in which the benefits and drawbacks of utilizing different AM techniques to fabricate various types of lattices are discussed. Subsequently, the relationships between the microarchitecture configurations and mechanical/physical properties of lattice metamaterials are summarized. This demonstrates that by customizing lattice microarchitectures and selecting appropriate constitutive materials, lattice metamaterials with various unconventional mechanical/physical properties can be formed. Moreover, the multifunctional applications of lattice metamaterials in various fields are enumerated, in which the interaction mechanisms between different mechanical/physical properties are summarized to reveal design guidelines for achieving simultaneous enhancement of their multi‐physical properties. Finally, challenges and future prospects regarding the expanded design principles, unexplored multi‐physical properties, and potential multifunctional applications of lattice metamaterials enabled by AM techniques are discussed, followed by a concise conclusion of the entire work.

## 2 Design Principles and Additive Manufacturing Procedures of Lattice Metamaterials

Various approaches have been proposed to devise lattice metamaterials that are exceptionally lightweight and also possess superior mechanical/physical properties. In addition, recent developments in AM techniques have overcome the difficulties in fabricating lattice metamaterials with intricate microarchitectures. In this section, the design principles and AM procedures of different types of lattice metamaterials, including homogeneous lattices, inhomogeneous lattices, and other forms of lattice metamaterials, are critically reviewed. The advantages and disadvantages of using different AM approaches to fabricate various types of lattices are also discussed.

### 2.1 Homogeneous Lattices

First, the design principles and AM procedures of homogeneous lattices are reviewed. These lattices are classified as truss, plate, shell, hybrid, and hierarchical lattices, based on the geometries/configurations of elementary structural units.

#### 2.1.1 Truss Lattices

**Figure 2:**
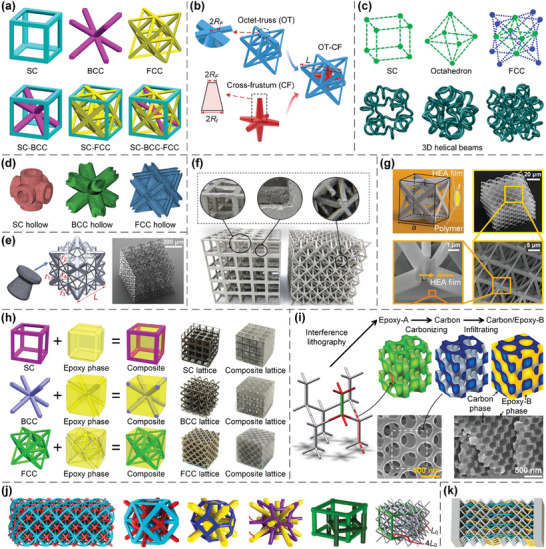
Design principles and AM processes of truss lattices. a) The fundamental SC, BCC, and FCC truss lattices within cubic symmetry group, and combined lattices from the three fundamental ones. Reproduced with permission.^[^ ^8^ ^]^ Copyright 2018, Elsevier. b) Combined truss lattices with variable cross sections for different constituent bars. Reproduced with permission.^[^ ^118^ ^]^ Copyright 2021, Elsevier. c) Truss lattices with curved‐beam struts. Reproduced with permission.^[^ ^29^ ^]^ Copyright 2020, Springer. d) Truss lattices with hollow cross sections of struts. Reproduced with permission.^[^ ^92^ ^]^ Copyright 2018, Elsevier. e) Truss lattices with different cross‐sectional radii for different constituent bars. Reproduced with permission.^[^ ^95^ ^]^ Copyright 2020, Elsevier. f) Additively manufactured truss lattice samples through a LPBF process. Reproduced with permission.^[^ ^90^ ^]^ Copyright 2020, Elsevier. g) The fabrication of composite nanolattices through DLW AM and HEA coating processes. Reproduced with permission.^[^ ^123^ ^]^ Copyright 2018, ACS. h) The fabrication of composite lattices made of SC, BCC, and FCC truss lattices and epoxy phase through LPBF AM and moulding processes. Reproduced with permission.^[^ ^124^ ^]^ Copyright 2020, Elsevier. i) The fabrication of composite lattices composed of variable cross‐sectional truss lattices and epoxy phase through interference lithography, carbonizing, and infiltrating. Reproduced with permission.^[^ ^125^ ^]^ Copyright 2012, ACS. j) Interpenetrating lattices composed of disconnected truss lattices. Reproduced with permission.^[^ ^127^ ^]^ Copyright 2021, Elsevier. k) Interpenetrating lattices composed of disconnected straight truss lattices and curved fibers. Reproduced with permission.^[^ ^127^ ^]^ Copyright 2021, Elsevier.

Truss lattices are a class of lattice metamaterials whose elementary structural units are periodic networks of bars connected through shared nodes.^[^ ^6^, ^7^, ^8^ ^]^ By tailoring their internal microarchitectures, truss lattices can be devised to exhibit stretching‐dominated deformation behaviors based on the well‐known Maxwell's criterion,^[^ ^6^, ^113^, ^114^, ^115^ ^]^ i.e., to bear external loads predominantly through the extension/shrinkage (instead of bending) of constituent bars. Typically, stretching‐dominated truss lattices outperform the earlier‐appearing bending‐dominated stochastic foams in stiffness and strength significantly.^[^ ^116^ ^]^ The most widely studied truss lattices comprise three elementary classes within cubic symmetry group:^[^ ^8^, ^9^ ^]^ simple cubic (SC), body‐centred cubic (BCC), and face‐centred cubic (FCC) (**Figure** **2**a). Stretching‐dominated octet truss lattices belong to FCC class, whereas SC and BCC lattices experience bending‐dominated deformations under an external load that is not aligned with the axial directions of their constituent bars. Various approaches have been devised to enlarge the design space of truss lattices such that they exhibit desired mechanical/physical properties. These approaches include the combination of fundamental lattices with tailored volume ratios^[^ ^8^, ^9^, ^117^ ^]^ (Figure 2a), the use of appropriately tailored variable cross sections for different constituent bars^[^ ^118^ ^]^ (Figure 2b), curved‐beam struts^[^ ^29^, ^119^, ^120^, ^121^ ^]^ (Figure 2c), hollow cross sections^[^ ^92^ ^]^ (Figure 2d), and different cross‐sectional radii for different constituent bars^[^ ^95^ ^]^ (Figure 2e). Among all target properties and functions, the design of truss lattices with isotropic elasticity, zero Poisson's ratio (ZPR) or negative Poisson's ratio (NPR), high deformability, and superior energy absorption capabilities are classic research topics.^[^ ^8^, ^9^, ^91^, ^92^, ^118^ ^]^

In recent decades, various AM techniques have emerged, facilitating the manufacture of truss lattices with intricate microarchitectures^[^ ^90^, ^91^, ^92^, ^93^, ^95^ ^]^ and significantly promoting their use in lightweight applications across a broad range of fields.^[^ ^118^, ^122^ ^]^ However, the fabrication of truss lattices via layer‐by‐layer AM processes, such as LPBF,^[^ ^90^, ^91^ ^]^ SLA,^[^ ^92^ ^]^ and FDM,^[^ ^93^ ^]^ is often limited by overhang constraints. These constraints generally result in large dimensional variations and surface roughnesses in constituent bars with small overhang angles, and some of these bars may become severely warped or even locally broken^[^ ^90^, ^91^ ^]^ (Figure 2f). Consequently, truss lattices should be appropriately rotated during these manufacturing procedures to overcome overhang constraints. Alternatively, the DLW procedure allows laser photons to penetrate deeply into constitutive materials and convert the liquid into a solid within the focal volume.^[^ ^94^ ^]^ Therefore, DLW offers a method for accumulating materials that differs significantly from the layer‐by‐layer manner, thereby directly addressing inherent limitations associated with overhang constraints^[^ ^94^ ^]^ and guaranteeing the fidelity of samples.^[^ ^95^ ^]^ Furthermore, composite nanolattices are fabricated from a polymer scaffold via DLW AM technique, followed by conformally coating the scaffold with high‐entropy alloy (HEA) films using magnetron sputtering deposition to overcome the strength‐recoverability trade‐off^[^ ^123^ ^]^ (Figure 2g). Besides, composite lattices made of SC, BCC, and FCC truss lattices and epoxy phase are devised and fabricated via LPBF AM and moulding processes to achieve enhanced strength and specific energy absorption^[^ ^124^ ^]^ (Figure 2h). Additionally, variable cross‐sectional truss lattices and epoxy phase are combined to form composite lattices through interference lithography, carbonizing, and infiltrating to achieve superior energy absorption capabilities^[^ ^125^ ^]^ (Figure 2i). Composite lattices composed of octet truss lattices and graphene aerogel phase are also devised and fabricated via low‐force SLA AM and hydrothermal processes to realize robust thermomechanical functionalities.^[^ ^126^ ^]^ In addition, interpenetrating lattices composed of disconnected truss lattices, and those composed of disconnected straight truss lattices and curved fibers are devised and fabricated by LPBF, PolyJet, DLW, and multi‐jet fusion AM processes to realize stress‐dependent resistivity, enhanced toughness, higher deformation ratio, and tailorable specific energy absorption^[^ ^127^, ^128^ ^]^ (Figure 2j,k).

#### 2.1.2 Plate Lattices

**Figure 3:**
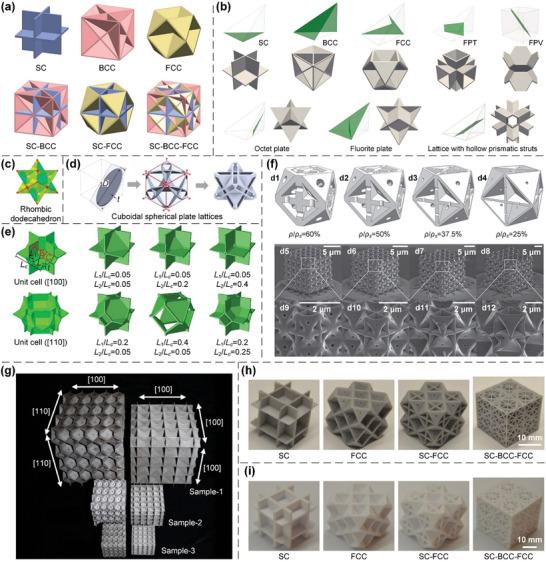
Design principles and AM processes of plate lattices. a) Elementary cubic plate lattices of SC, BCC, and FCC classes are assembled with appropriate volume ratios to generate combined lattices with superior isotropic elasticity. Reproduced with permission.^[^ ^11^ ^]^ Copyright 2018, Wiley. b) A family of parametric plate lattices are presented to enlarge the design space to achieve target mechanical properties. Reproduced with permission.^[^ ^134^ ^]^ Copyright 2023, Elsevier. c) A series of rhombic dodecahedron plate lattices are devised to achieve both isotropic elasticity and nearly isotropic yield strength. Reproduced with permission.^[^ ^136^ ^]^ Copyright 2024, Elsevier. d) A set of cuboidal spherical plate lattices are topologically optimized to achieve enhanced stiffness and strength. Reproduced with permission.^[^ ^137^ ^]^ Copyright 2024, Taylor & Francis. e) A series of half‐open‐cell plate lattices are proposed to enhance the additive manufacturability, with a moderate reduction in mechanical properties. Reproduced with permission.^[^ ^138^ ^]^ Copyright 2020, Elsevier. The fabrication of plate lattices through f) DLW, g) LPBF, h) SLA, and i) FDM AM processes. Reproduced with permission.^[^ ^12^ ^]^ Copyright 2020, Springer. Reproduced with permission.^[^ ^138^ ^]^ Copyright 2020, Elsevier. Reproduced with permission.^[^ ^139^ ^]^ Copyright 2021, Elsevier. Reproduced with permission.^[^ ^97^ ^]^ Copyright 2021, Elsevier.

Despite numerous studies on the design of truss lattices to enhance their mechanical/physical properties, the stiffness and strength of truss lattices remain far below theoretical upper bounds since they bear external loads through the uniaxial extension/shrinkage of constituent bars.^[^ ^8^, ^9^, ^129^, ^130^ ^]^ Consequently, plate lattices, another class of lattice metamaterials whose elementary structural units are periodic networks of plates connected through shared edges, have emerged and exhibited superior mechanical/physical properties to those of truss lattices.^[^ ^10^, ^11^, ^12^, ^130^ ^]^ Recently, a series of single‐scale combined plate lattices have been devised by combing elementary SC, BCC, and FCC lattices with appropriate volume ratios to achieve superior isotropic elasticity^[^ ^10^, ^11^, ^12^ ^]^ (**Figure** **3**a). These combined lattices are stretching‐dominated under arbitrary external loads and undergo bi‐directional extension/shrinkage within the tangent planes of constituent plates. Therefore, they can be appropriately devised to approach the stiffness and strength upper bounds at low relative densities.^[^ ^131^, ^132^ ^]^ Additionally, dual‐scale combined plate lattices can be devised through the combination of elementary lattices with different length scales, which exhibit significantly superior energy absorption capabilities to their single‐scale counterparts.^[^ ^133^ ^]^ Moreover, a family of parametric plate lattices have been further developed to enlarge the design space to achieve tailored mechanical/physical properties (Figure 3b). The parametric model covers the widely‐known SC, BCC, and FCC lattices, and also includes the less studied Fluorite plate, flat plate tesseract (FPT), and flat plate vintile (FPV) lattices.^[^ ^134^, ^135^ ^]^ Furthermore, a family of rhombic dodecahedron plate lattices are devised to achieve isotropic elasticity and nearly isotropic yield strength^[^ ^136^ ^]^ (Figure 3c), and a set of cuboidal spherical plate lattices are topologically optimized to achieve enhanced stiffness and strength^[^ ^137^ ^]^ (Figure 3d). However, the closed‐cell features of these lattices complicate their AM processes and applications.^[^ ^11^, ^96^ ^]^ Thus, half‐open‐cell plate lattices were devised to enhance the additive manufacturability (Figure 3e), with a moderate reduction in stiffness and strength.^[^ ^138^ ^]^ The complexity of the AM procedures of plate lattices is further illustrated by DLW^[^ ^12^ ^]^ (Figure 3f), LPBF^[^ ^138^ ^]^ (Figure 3g), and SLA^[^ ^139^ ^]^ (Figure 3h) processes, in which additional holes must be installed for the removal of unmolten metal powders or residual resins within closed internal cavities. Numerical simulations showed that these small holes only resulted in a slight reduction in stiffness and strength.^[^ ^11^ ^]^ In comparison, FDM process constructs an object through the gradual deposition of a fine filament of polymers via a programmable print head, and exhibits significantly superior additive manufacturability to the other AM processes of plate lattices,^[^ ^97^ ^]^ as no additional holes are required (Figure 3i). In addition, combining 3D printing and glue‐free assembly, auxetic plate lattices can be devised to achieve numerous advantages, such as disassembly, assembly, replacement, recyclability, reusability, high specific stiffness, superior energy absorption capability, and exceptional impact resistance performances.^[^ ^140^ ^]^

#### 2.1.3 Shell Lattices

**Figure 4:**
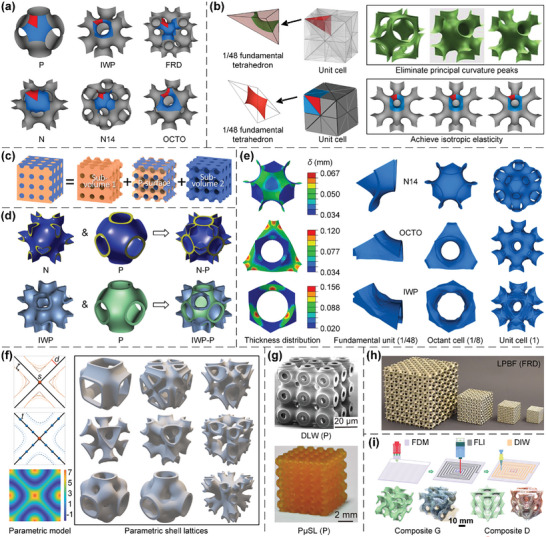
Design principles and AM processes of shell lattices. a) TPMS P, IWP, FRD, N, N14, and OCTO surfaces are generated within the 1/48 fundamental domain (red), 1/8 unit cell (blue), and unit cell (grey) via the open‐source Surface Evolver software, respectively. Reproduced with permission.^[^ ^96^ ^]^ Copyright 2021, Elsevier. b) The design of shell lattices through shape optimization of mid‐surfaces. Reproduced with permission.^[^ ^14^ ^]^ Copyright 2019, Elsevier. Reproduced with permission.^[^ ^143^ ^]^ Copyright 2022, Elsevier. c) P‐surface as a TPMS that splits a cube‐like space into two sub‐volumes bounded by the blue‐ and orange‐shaded surfaces. Reproduced with permission.^[^ ^144^ ^]^ Copyright 2024, Springer. d) The design of shell lattices by combining elementary TPMS lattices with appropriate volume ratios. Reproduced with permission.^[^ ^16^ ^]^ Copyright 2019, Elsevier. Reproduced with permission.^[^ ^145^ ^]^ Copyright 2021, Elsevier. e) The variable thickness design of shell lattices. Reproduced with permission.^[^ ^96^ ^]^ Copyright 2021, Elsevier. f) A parametric shell lattice model is proposed to enlarge the design space of shell lattices to obtain target mechanical properties. Reproduced with permission.^[^ ^148^ ^]^ Copyright 2022, Elsevier. The fabrication of TPMS shell lattices through g) DLW, PµSL, and h) micro‐LPBF AM processes. Reproduced with permission.^[^ ^15^ ^]^ Copyright 2022, PNAS. Reproduced with permission.^[^ ^98^ ^]^ Copyright 2019, Elsevier. i) A multi‐material process that synergistically combines the advantages of FDM, FLI, and DIW AM techniques to seamlessly integrate both structural and laser‐processable functional materials into 3D engineered shell lattices with intricate geometries. Reproduced with permission.^[^ ^152^ ^]^ Copyright 2024, Springer.

Although plate lattices are capable of approaching the theoretical upper bounds of stiffness and strength, the closed‐cell feature complicates their fabrication via most AM processes^[^ ^12^, ^138^, ^139^ ^]^ (Figure 3f‐h), and limits their applications in areas with increasing demands for mass and heat transfer.^[^ ^96^ ^]^ In this case, shell lattices, a class of lattice metamaterials whose elementary structural units are periodic networks of continuous, smooth, and non‐intersecting shells, attract increasing attention.^[^ ^13^, ^14^, ^15^ ^]^ Shell lattices are generally free from stress concentration owing to their smooth geometries, and open‐cell features facilitate their manufacture and use in lightweight multifunctional applications significantly.^[^ ^76^, ^141^, ^142^ ^]^ To date, triply periodic minimal surfaces (TPMSs), a well‐known family of surfaces with constant zero mean curvature, infinite repeatability in three orthogonal directions, and local minimization of surface area, are most widely studied in shell lattices.^[^ ^76^, ^141^, ^142^ ^]^ The six representative types of TPMSs, namely Primitive (P), I‐graph‐wrapped package (IWP), FCC rhombic dodecahedron (FRD), Neovius (N), N14, and OCTO surfaces, are illustrated in **Figure** **4**a.^[^ ^96^ ^]^ These surfaces are both reflectionally symmetric about the three orthogonal mid‐planes and rotationally symmetric about the three orthogonal axes, thus the unit cell can be divided into at least 48 equal subdomains.^[^ ^96^ ^]^ Accordingly, several numerical approaches were developed for optimizing the shell mid‐surface shape in the 1/48 fundamental tetrahedron to obtain target geometric features and mechanical/physical properties, including the elimination of principal curvature peaks^[^ ^14^, ^98^ ^]^ and the realization of isotropic elasticity^[^ ^143^ ^]^ (Figure 4b). Specifically, as TPMSs can split a cube‐like space into two neighboring non‐intersecting volumes, they can be suitably devised for enhancing the mass and heat transfer efficiency in case of multifunctional applications like pressure vessels^[^ ^144^ ^]^ (Figure 4c). Additionally, elementary TPMS shell lattices can be directly combined with appropriate volume ratios to achieve different target properties^[^ ^16^, ^145^, ^146^ ^]^ (Figure 4d). Alternatively, the wall thickness can be selected as an appropriate design variable, based on which an optimization procedure is developed to seek superior isotropic elasticity^[^ ^96^, ^147^ ^]^ (Figure 4e). Moreover, a parametric shell lattice model, which enables a much larger design space than conventional TPMS shell lattices, is proposed to achieve tailored mechanical/physical properties, including isotropic elasticity, enhanced stiffness, tailored Poisson's ratio, etc.^[^ ^148^, ^149^ ^]^ (Figure 4f). Alternatively, shell lattices can also be devised by introducing slight corrugations into plate lattices, which enables to achieve superior isotropic elasticity and a remarkable enhancement in strength and specific energy absorption.^[^ ^150^, ^151^ ^]^

Generally, the additive manufacturability of shell lattices is supeior to truss and plate lattices. For example, open‐cell features enable the direct removal of unmolten metal powders or residual resins produced in metal powder or liquid resin‐based AM processes,^[^ ^15^, ^96^, ^98^ ^]^ such that shell lattices exhibit better additive manufacturability than closed‐cell plate lattices. Moreover, the smoothly connected surfaces of shell lattices overcome overhang constraints and result in high fidelity in samples,^[^ ^90^, ^91^, ^99^ ^]^ such that the fabrication quality of shell lattices is substantially better than truss lattices. The projection micro‐stereolithography (PµSL) and DLW fabricated polymeric samples of P shell lattices^[^ ^15^ ^]^ and the micro‐LPBF fabricated metallic samples of FRD shell lattices^[^ ^98^ ^]^ are illustrated in Figure 4g,h, respectively. Recently, a multi‐material process that synergistically combines the advantages of FDM, freeform laser induction (FLI), and direct ink writing (DIW) AM techniques has been proposed to integrate laser‐processable and structural functional materials into 3D composite shell lattices with intricate geometries seamlessly^[^ ^152^ ^]^ (Figure 4i). All of these samples demonstrate superior fidelity and fabrication quality.

#### 2.1.4 Hybrid Lattices

**Figure 5:**
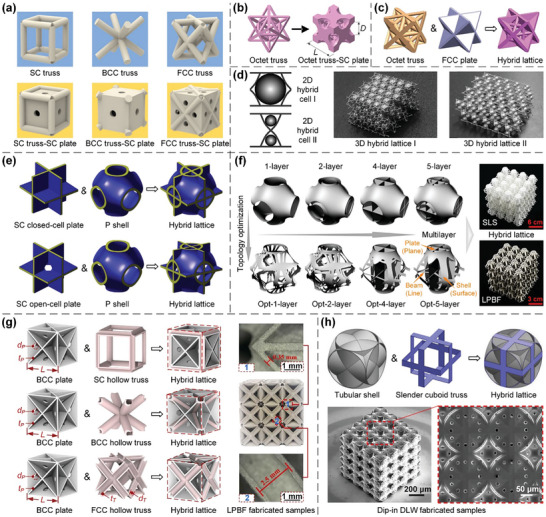
Design principles and AM processes of hybrid lattices. a) SC, BCC, and FCC truss lattices are hybridized with SC plate lattices to enhance the specific energy absorption and broadband vibration attenuation. Reproduced with permission.^[^ ^153^ ^]^ Copyright 2024, Taylor & Francis. b) Elastically isotropic hybrid open‐cell lattices are devised by combining octet truss and SC plate lattices. Reproduced with permission.^[^ ^154^ ^]^ Copyright 2023, Elsevier. c) Octet truss and FCC plate lattices are appropriately hybridized to enhance the stiffness, strength, and energy absorption capabilities simultaneously. Reproduced with permission.^[^ ^17^ ^]^ Copyright 2021, Wiley. d) MHS arrays are hybridized into the small octahedrons or tetrahedrons of WBK lattices' interior spaces to achieve exceptional energy absorption capabilities. Reproduced with permission.^[^ ^18^ ^]^ Copyright 2011, Wiley. e) Closed‐cell and open‐cell SC plate lattices and P shell lattices are hybridized with appropriate volume fractions to achieve superior isotropic elasticity. Reproduced with permission.^[^ ^16^ ^]^ Copyright 2019, Elsevier. f) Ultra‐stiff truss‐plate‐shell hybrid lattices with a reasonable distribution of materials are first devised through a multilayer strategy and topology optimization framework, and then fabricated via SLS in nylon (PA12) and TPU and LPBF in SS316L and AiSi10Mg. Reproduced with permission.^[^ ^157^ ^]^ Copyright 2024, Springer. g) Hybrid open‐cell lattices are first devised by combining porous BCC plate and SC, BCC, and FCC truss lattices, and then fabricated via LPBF in SS316L. Reproduced with permission.^[^ ^158^ ^]^ Copyright 2023, Elsevier. h) Tubular shell and slender cuboid truss lattices are first hybridized and then fabricated via dip‐in DLW in photosensitive polymers to enhance the elastic modulus, yield strength, and energy absorption capability. Reproduced with permission.^[^ ^159^ ^]^ Copyright 2022, Elsevier.

Hybrid lattices, another class of lattice metamaterials formed via combining at least two different types of structures among truss, plate, and shell lattices with the same length scale, offer another design alternative to achieve tailored mechanical/physical properties. For example, SC, BCC, and FCC truss lattices can be hybridized with SC plate lattices to enhance the specific energy absorption and broadband vibration attenuation^[^ ^153^ ^]^ (**Figure** **5**a). Octet truss and SC plate lattices can be hybridized with appropriate volume ratios to achieve superior isotropic elasticity^[^ ^154^ ^]^ (Figure 5b), and octet truss and FCC plate lattices can be appropriately hybridized to simultaneously enhance the stiffness, strength, and energy absorption capabilities^[^ ^17^ ^]^ (Figure 5c). Besides, metal hollow sphere (MHS) arrays can be hybridized into the small octahedrons or tetrahedrons of wire‐woven bulk Kagome (WBK) lattices' interior spaces, resulting in two typical types of truss‐shell hybrid lattices^[^ ^18^ ^]^ (Figure 5d). Owing to the moving plastic hinges in MHSs and the suppression of strut buckling in WBK lattices, these hybrid lattices exhibit exceptional energy absorption capabilities. Similarly, spheres can be placed inside FCC truss lattices to generate hybrid lattices with enhanced mechanical properties, and interpenetrating lattices composed of disconnected Gyroid (G) shell lattices and rhombic dodecahedron truss lattices are devised to achieve multifunctionality.^[^ ^127^, ^155^ ^]^ Additionally, truss lattices can be utilized to accommodate the void space of shell lattices to overcome their buckling instability.^[^ ^156^ ^]^ Furthermore, closed‐cell and open‐cell SC plate and P shell lattices can be hybridized with appropriate volume fractions to achieve superior isotropic elasticity^[^ ^16^ ^]^ (Figure 5e). Recently, a multilayer strategy and topology optimization framework was developed to automatically generate a series of ultra‐stiff truss‐plate‐shell hybrid lattices, which exhibited a reasonable distribution of materials^[^ ^157^ ^]^ (Figure 5f). Typically, closed‐cell hybrid lattices complicate the AM procedures since extra holes should be installed to enhance additive manufacturability^[^ ^16^ ^]^ (Figure 5a,b,e), whereas open‐cell hybrid lattices facilitate the AM procedures directly^[^ ^17^, ^18^ ^]^ (Figure 5c,d,f). The open‐cell truss‐plate‐shell hybrid lattices are manufactured via selective laser sintering (SLS) in nylon (PA12) and thermoplastic polyurethane (TPU) as well as LPBF in stainless steel 316 L (SS316L) and aluminium alloy (AiSi10Mg)^[^ ^157^ ^]^ (Figure 5f), and the open‐cell lattices hybridized from porous BCC plate and SC, BCC, and FCC hollow truss lattices are fabricated via LPBF in SS316L^[^ ^158^ ^]^ (Figure 5g). Furthermore, perforated lattices hybridized from tubular shell and slender cuboid truss lattices are devised to improve the stiffness, compressive strength, and energy absorption capability, and then fabricated via dip‐in DLW in photosensitive polymers^[^ ^159^ ^]^ (Figure 5h). All of these samples demonstrate superior fabrication quality and fidelity.

#### 2.1.5 Hierarchical Lattices

Hierarchical lattices, which feature organized microarchitectures across well‐ordered structural levels and different length scales, usually exhibit properties differing from those of their constitutive materials and single‐scale counterparts.^[^ ^109^, ^160^, ^161^, ^162^ ^]^ Natural hierarchical lattices are typically created through biologically controlled self‐assembly processes that allow microarchitectures to grow to meet local requirements,^[^ ^163^ ^]^ and their hierarchical structures enable multifunctionality and dynamic responses to external stimuli.^[^ ^164^, ^165^, ^166^ ^]^ In contrast, artificial hierarchical lattices are usually designed according to functional prerequisites and manufacturing constraints, which can be controlled and tuned independently by introducing hierarchy‐related parameters, such as unit cell length ratio,^[^ ^167^ ^]^ hierarchical order,^[^ ^168^ ^]^ etc.

**Figure 6:**
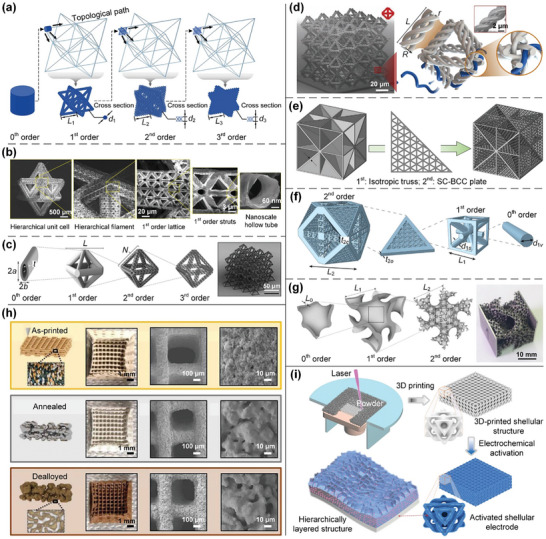
Design principles of various hierarchical lattices. a) 3D hierarchical structures with octet truss lattices as multi‐order configurations. Reproduced with permission.^[^ ^168^ ^]^ Copyright 2019, Elsevier. b) 3D hierarchical structures with octet truss and octet hollow truss as the first‐order and second‐order configurations. Reproduced with permission.^[^ ^20^ ^]^ Copyright 2016, Springer. c) 3D hierarchical structures with octet truss as the first‐order configuration and octahedron hollow truss as the second‐ and third‐order configurations. Reproduced with permission.^[^ ^19^ ^]^ Copyright 2015, PNAS. d) 3D hierarchical structures with octahedron truss and interwoven curved fibers as the first‐order and second‐order configurations. Reproduced with permission.^[^ ^121^ ^]^ Copyright 2020, Wiley. e) 3D hierarchical structures with SC‐BCC plate and 2D isotropic truss as the first‐order and second‐order configurations. Reproduced with permission.^[^ ^170^ ^]^ Copyright 2021, Elsevier. f) 3D hierarchical structures with SC‐FCC plate and 3D isotropic truss as the first‐order and second‐order configurations. Reproduced with permission.^[^ ^171^ ^]^ Copyright 2023, Wiley. g) 3D hierarchical structures with TPMS shell lattices as the first‐ and second‐order configurations. Reproduced with permission.^[^ ^167^ ^]^ Copyright 2021, Elsevier. h) Hierarchical porous lattices generated via dealloying after 3D printing. Reproduced with permission.^[^ ^178^ ^]^ Copyright 2018, AAAS. i) Hierarchical layered lattices generated via electrochemical activation after 3D printing. Reproduced with permission.^[^ ^179^ ^]^ Copyright 2021, Wiley.

Due to the constraints of manufacturing techniques, prior studies on hierarchical lattices mainly focus on hierarchical honeycombs^[^ ^169^ ^]^ and hierarchical plate lattices with perforated sheets.^[^ ^170^ ^]^ Compared with other lattices, hierarchical lattices allow a larger flexibility of geometric parameters at all levels, but their design methodologies remain limited. The most widely adopted approach for constructing hierarchical lattices is to recursively fill their upper‐scale structural elements using fundamental lattices,^[^ ^161^, ^162^ ^]^ such as trusses, plates, or shells. Accordingly, hierarchical lattices are classified according to the geometries/configurations of their elementary structural units as truss‐based^[^ ^19^, ^20^, ^121^, ^168^ ^]^ (**Figure** **6**a–d), plate‐based^[^ ^170^, ^171^ ^]^ (Figure 6e,f), and shell‐based^[^ ^167^, ^172^ ^]^ (Figure 6g) lattices. As a typical class of 3D hierarchical lattices, truss‐based hierarchical lattices possess excellent mechanical properties, such as superior compressive strength^[^ ^168^ ^]^ (Figure 6a), elasticity^[^ ^20^ ^]^ (Figure 6b), and recoverability^[^ ^19^ ^]^ (Figure 6c). Specifically, hierarchical woven truss lattices exhibit high tensile/compressive strains before the onset of failure, which combined the high toughness of woven structures and superior strength of truss lattices^[^ ^121^ ^]^ (Figure 6d). Furthermore, a class of hierarchical curved woven truss lattices with lower dynamic compressive strength, superior energy absorption capability, and programmable dynamic loading performance were engineered using an AM‐oriented design strategy and machine learning approaches.^[^ ^173^ ^]^ Besides, truss lattices with micro‐ or nano‐architectures also exhibit other numerous beneficial mechanical^[^ ^174^ ^]^ and optical^[^ ^175^ ^]^ properties. Typically, plate lattices possess a high degree of mechanical efficiency, and the combination of 3D isotropic plate lattices with 2D isotropic trusses or 3D conformal isotropic truss lattices with smaller length scales results in isotropic hierarchical lattices with superior stiffness and strength^[^ ^170^, ^171^ ^]^ (Figure 6e,f). Regarding 3D hierarchical shell‐based lattices, the mid‐surfaces of TPMS shell lattices possess a locally minimized surface area for a given boundary, in which each point on the surface possesses a constant zero mean curvature.^[^ ^176^ ^]^ Hierarchical TPMS shell lattices are usually constructed via recursive Boolean operations^[^ ^167^ ^]^ (Figure 6g), and they generally exhibit more exceptional stiffness, buckling strength, plateau stress, and energy absorption capabilities than hierarchical truss lattices, with more noticeable advantages at lower relative densities.^[^ ^177^ ^]^ Owing to the high‐resolution requirements of hierarchical lattices with large aspect ratios, most of their fabrication methods are based on photopolymerization processes.^[^ ^19^, ^20^ ^]^ Subsequently, dealloying and electrochemical activation are used to construct hierarchical lattices on additively manufactured parts (Figure 6h,i), which enables a substantial decrease of the feature size into micro or even nano scales and a significant increase in surface area.^[^ ^178^, ^179^ ^]^

The introduction of hierarchical principles into the design of synthetic lattices provides advantages in mechanical and structural properties.^[^ ^180^ ^]^ The enhancement of mechanical properties is generally due to (1) the suppression of brittle fracture failure, (2) the suppression of elastic buckling failure of upper‐scale structures, (3) the mitigation of loss in connectivity, and (4) the tunability of (an)isotropy. Typically, ceramics, which are characterized by high stiffness and strength with low toughness, exhibit brittle failure. However, by controlling the aspect ratios of hollow tubes in ceramics, their failure modes can be transitioned from brittle fracture to shell buckling.^[^ ^33^ ^]^ The incorporation of hierarchical microarchitectures can also enable micro‐level elastic buckling to accommodate large external strains without fracture, indicating superior recoverability.^[^ ^19^ ^]^ Additionally, the presence of multiple hierarchies can enhance their buckling strength at the microscale, shifting the failure mode from structure buckling to material yielding, thereby enhancing compressive strength at ultra‐low relative densities.^[^ ^167^ ^]^ Moreover, hierarchical microarchitectures can enhance the resistance to loss in connectivity^[^ ^181^ ^]^ and lead to smooth stress‐strain curves without significant stress fluctuations under large deformations.^[^ ^172^ ^]^ Furthermore, isotropic hierarchical lattices can be constructed from anisotropic lattices at different length scales.^[^ ^161^, ^171^ ^]^ For example, by tuning the relative densities of microarchitectures with different length scales, cell length ratio, and hierarchical order number, hierarchical TPMS shell lattices can be tailored to achieve isotropic elasticity.^[^ ^161^ ^]^ Besides, hierarchical plate lattices, devised through the introduction of 3D conformal trusses at the microscale, exhibit greater isotropic elasticity, stiffness, and strength than those with fractal geometries.^[^ ^171^ ^]^

### 2.2 Inhomogeneous Lattices

In addition to homogeneous lattices, the design principles and AM procedures of inhomogeneous lattices are reviewed in this section. Based on the architected patterns of microarchitectures, inhomogeneous lattices are classified as graded and heterogeneous lattices.

#### 2.2.1 Graded Lattices

**Figure 7:**
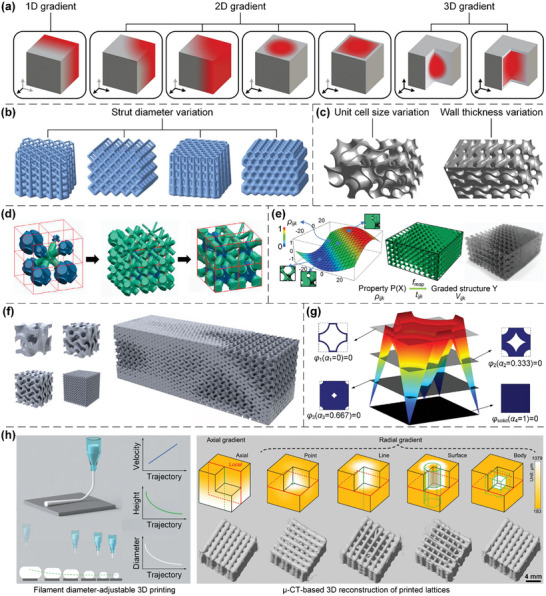
Design principles of various graded lattices. a) Graded lattices with 1D, 2D, and 3D gradient distribution of design parameters. Reproduced with permission.^[^ ^182^ ^]^ Copyright 2018, Elsevier. b) Graded truss lattices with 1D gradient variation of strut diameters. Reproduced with permission.^[^ ^187^ ^]^ Copyright 2017, Elsevier. c) Graded shell lattices with gradient variation of unit cell size and wall thickness. Reproduced with permission.^[^ ^184^ ^]^ Copyright 2022, Wiley. d) Graded truss lattices with 3D multi‐furcation gradient geometries. Reproduced with permission.^[^ ^183^ ^]^ Copyright 2019, Elsevier. e) 3D graded cellular structures devised by mapping mechanical properties using the material density function. Reproduced with permission.^[^ ^191^ ^]^ Copyright 2018, Elsevier. f) Components filled with graded TPMS shell lattices based on an optimized density field. Reproduced with permission.^[^ ^189^ ^]^ Copyright 2023, IEEE. g) Graded lattices determined by the zero‐contours of implicit level set functions. Reproduced with permission.^[^ ^190^ ^]^ Copyright 2018, Elsevier. h) Conventional extrusion‐based 3D printers can be employed to fabricate 1D, 2D, and 3D graded lattices by adjusting the filament diameters throughout the printing process, which is achieved by altering the amount of ink placed upon the printing trajectory. Reproduced with permission.^[^ ^192^ ^]^ Copyright 2024, Springer.

Functionally graded lattices (FGLs) are inhomogeneous lattices devised with varying geometric factors in the design domain.^[^ ^182^ ^]^ FGLs have been extensively used as highly customizable, tuneable, and multifunctional designs for lattice metamaterials.^[^ ^182^, ^183^, ^184^, ^185^ ^]^ Recent developments in AM techniques have made it possible to fabricate graded lattices with intricate microarchitectures and facilitated the integration of multifunctionalities into a single component via the graded design concept.^[^ ^186^ ^]^ The gradient types of graded lattices are classified as one‐dimensional (1D), 2D, and 3D gradients, according to their spatial patterns of gradient distribution^[^ ^182^ ^]^ (**Figure** **7**a). The most commonly used design parameters of FGLs are the dimensions of mesoscale gradient vectors, and the parameters of microarchitectures, including volume fraction, shape, orientation, and size.^[^ ^187^, ^188^ ^]^

To date, many approaches have been developed to design/optimize internal microarchitectures to enhance the mechanical/physical properties and broaden the applications of graded lattices. Connectivity is a crucial factor in the design process, which ensures the smooth transition and water‐tight connection between neighbouring unit cells with varying geometric factors. By adopting 1D gradient distribution of strut diameters and ensuring the connectivity between neighbouring cells, graded truss lattices can be devised to exhibit novel localized and gradual failure mode and enhanced plateau stress and specific energy absorption^[^ ^187^ ^]^ (Figure 7b). Typically, TPMS shell lattices exhibit better connectivity, which can be achieved by controlling the connectivity of implicit function parameters.^[^ ^76^, ^184^ ^]^ The relative densities of TPMS shell lattices can be fine‐tuned by varying unit cell sizes or wall thicknesses, which enables to enhance their mechanical and thermal properties (Figure 7c). Additionally, B‐spline surfaces, controlled by polygons, are used to generate multi‐furcation gradient geometries^[^ ^183^ ^]^ (Figure 7d), in which water‐tight boundaries between neighbouring unit cells can be ensured by creating polyhedron net distributions. Moreover, topology optimization serves as a key strategy to generate appropriate gradient layouts for devising lightweight graded lattices.^[^ ^28^, ^189^, ^190^, ^191^ ^]^ 3D functionally graded cellular structures can be devised by mapping their mechanical properties utilizing the material density function, which can be further incorporated into topology optimization frameworks for optimizing their mechanical/physical properties^[^ ^191^ ^]^ (Figure 7e). Similarly, A SIMP‐based optimization approach results in an optimized distribution of densities, which are further mapped to a macrostructure, resulting in a modified component with optimized density distribution^[^ ^28^, ^189^ ^]^ (Figure 7f). Alternatively, graded lattices can also be constructed via the zero‐contours of implicit level set functions, based on which geometric factors can be directly incorporated into the optimization process^[^ ^190^ ^]^ (Figure 7g). By implementing this strategy, the computational cost of solving downscale homogenization equations can be substantially decreased, thus leading to an enhanced efficiency for the computational and design process.^[^ ^190^ ^]^ Furthermore, conventional extrusion‐based 3D printers can be employed to fabricate 1D, 2D, and 3D graded lattices by adjusting the filament diameters throughout the printing process, which is achieved by altering the amount of ink placed upon the printing trajectory^[^ ^192^ ^]^ (Figure 7h). Additionally, an adaptive nozzle technique enables to alter the nozzle diameters and cross‐sectional shapes during the printing process dynamically, facilitating the manufacture of continuously graded parts, eliminating the sharp change/transition between different geometries, and resulting in higher precision in densities and contours than conventional 3D printing techniques.^[^ ^193^ ^]^ In addition, a 3D‐printed digital material filament consisting of various constitutive materials mixed in specific concentrations and distributions is developed towards 3D printing of FGLs, utilizing the readily available FDM printer.^[^ ^194^ ^]^

#### 2.2.2 Heterogeneous Lattices

Heterogeneity, which refers to the nonuniform distribution of features within a solid, is different from anisotropy.^[^ ^195^ ^]^ Heterogeneity in additively manufactured lattice metamaterials is typically manifested by the interconnections between neighbouring unit cells with different microarchitecture configurations or constitutive materials, in which different compositions, properties, and functionalities are precisely arranged in 3D space. Particularly, geometric parameters, including topology,^[^ ^26^, ^196^ ^]^ thickness,^[^ ^197^ ^]^ and orientation,^[^ ^23^, ^198^ ^]^ can be fine‐tuned to generate a diversity of heterogeneous lattices. Due to their varying mechanical,^[^ ^25^, ^26^ ^]^ conductive,^[^ ^199^ ^]^ biomimetic,^[^ ^200^ ^]^ and chemical^[^ ^25^, ^201^ ^]^ properties at different locations, heterogeneous lattices generally provide designers with a larger design freedom to explore a wider range of applications for lattice metamaterials.

**Figure 8:**
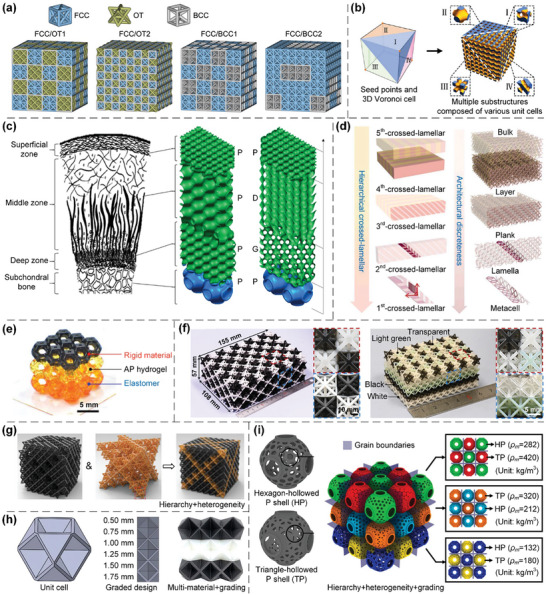
Design principles of various heterogeneous lattices. a) Heterogeneous truss lattices composed of different unit cells with the voxel distribution. Reproduced with permission.^[^ ^23^ ^]^ Copyright 2023, Wiley. b) Heterogeneous shell lattices composed of different unit cells with 3D voxel distribution. Reproduced with permission.^[^ ^204^ ^]^ Copyright 2023, ACS. c) Heterogeneous TPMS scaffolds with different microarchitecture configurations in four adjacent regions. Reproduced with permission.^[^ ^200^ ^]^ Copyright 2018, ACS. d) Bio‐inspired heterogeneous truss lattices with the crossed‐lamellar design. Reproduced with permission.^[^ ^198^ ^]^ Copyright 2024, Elsevier. e) Heterogeneous lattices of layered design, illustrated by a printed Kelvin foam consisting of rigid polymer, acrylamide‐poly(ethylene glycol) diacrylate (PEGDA) (AP) hydrogel, and elastomer. Reproduced with permission.^[^ ^205^ ^]^ Copyright 2021, AAAS. f) Heterogeneous lattices with the voxel distribution of unit cells with different constitutive materials. Reproduced with permission.^[^ ^25^ ^]^ Copyright 2022, Springer. g) Hierarchical heterogeneous truss lattices. Reproduced with permission.^[^ ^206^ ^]^ Copyright 2023, ACS. h) Graded heterogeneous plate lattices. Reproduced with permission.^[^ ^207^ ^]^ Copyright 2023, Elsevier. i) Hierarchical graded heterogeneous shell lattices. Reproduced with permission.^[^ ^208^ ^]^ Copyright 2022, Elsevier.

Herein, heterogeneity induced by microarchitecture configurations is primarily considered, in which different types of lattices are combined to achieve specific mechanical/physical properties. The deformation behaviors and collapse modes of lattice metamaterials under large strains are generally controllable. Homogeneous stretching‐dominated truss lattices typically exhibit global failure behavior, while the introduction of heterogeneous microarchitectures can inhibit global failure to enhance energy absorption capabilities^[^ ^23^, ^202^, ^203^ ^]^ (**Figure** **8**a). An FCC truss lattice with partially interpenetrated rhombic dodecahedron truss lattice can be used to control the crushing mode under compressive loads.^[^ ^127^ ^]^ By fully connecting different types of TPMS substructures within a cubic unit cell, the resulting heterogeneous shell lattices can exhibit tuneable mechanical properties. The energy absorption range can also be significantly enlarged^[^ ^204^ ^]^ (Figure 8b). Besides, different classes of TPMS shell lattices can be seamlessly interconnected in adjacent regions for achieving multifunctionalities, including tailorable mechanical stiffness, tissue‐specific properties, biomimetic functional gradients, etc.^[^ ^200^ ^]^ (Figure 8c). The large surface area to volume ratios and interconnected surfaces enable to enhance permeability and alleviate stress shielding. Additionally, as an inspiration from the 3D heterogeneous microarchitectures of *Strombus gigas*, heterogeneous truss lattices with crossed‐lamellar designs are proposed, which effectively reduce the risk of catastrophic failure caused by the unchecked extension of a single shear band and significantly enhance the strength and toughness^[^ ^198^ ^]^ (Figure 8d). Moreover, heterogeneous lattices can be obtained by combing microarchitectures with different constitutive materials within a lattice, such as hydrogels, functional polymers, and ceramics.^[^ ^25^ ^]^ Through the interplay between microarchitecture configurations and constitutive materials, heterogeneous lattices can be tailored to achieve different unique properties. For example, heterogeneous lattices can be generated via periodic arrangements of representative volume elements with different constitutive materials to achieve unique properties^[^ ^25^, ^205^ ^]^ (Figure 8e,f). The integration of hydrogel‐polymer lattices with additional polymers can enhance the strength of hydrogel lattices and introduce new functionalities. For instance, hydrogel‐based lattices with heterogeneous mechanical properties were devised by integrating rigid polymers, acrylamide‐poly(ethylene glycol) diacrylate (PEGDA) (AP) hydrogels, and elastomers.^[^ ^205^ ^]^ Besides, flexible ionic conductive octet truss lattices composed of an ionic conductive elastomer core surrounded by nonconductive soft polymer components were also devised, which substantially enhanced the wearability of ionic conductive lattices.^[^ ^25^ ^]^ Surprisingly, incorporating heterogeneity with hierarchical and graded designs can remarkably enhance the mechanical properties of lattices, including the design of hierarchical heterogeneous truss lattices to achieve enhanced stiffness and plateau stress^[^ ^206^ ^]^ (Figure 8g), the design of graded heterogeneous plate lattices to achieve tuneable energy absorption capabilities^[^ ^207^ ^]^ (Figure 8h), and the design of hierarchical graded heterogeneous shell lattices to achieve a layer‐wise failure mode, which significantly enhances the specific energy absorption^[^ ^208^ ^]^ (Figure 8i).

### 2.3 Other Forms of Lattice Metamaterials

In addition to the homogeneous structural units and inhomogeneous design methods mentioned above, researchers have also explored novel 2D and 3D lattice metamaterials with other innovative deformation mechanisms and superior mechanical/physical properties. The relevant design principles include planar tessellations and spatial packings theory,^[^ ^209^ ^]^ dimensional reduction and exfoliation,^[^ ^210^ ^]^ dimensional increase and reconstruction,^[^ ^211^ ^]^ topological analysis and optimization,^[^ ^212^, ^213^ ^]^ artificial intelligence (AI),^[^ ^214^, ^215^ ^]^ taking inspiration from nature,^[^ ^216^ ^]^ etc. The representative structural design forms include ordered honeycombs,^[^ ^217^ ^]^ hierarchical honeycombs,^[^ ^218^ ^]^ Kagome lattices,^[^ ^219^, ^220^ ^]^ chiral/anti‐chiral designs,^[^ ^221^ ^]^ Janus structures,^[^ ^222^ ^]^ filling structures,^[^ ^223^, ^224^ ^]^ cylindrical configurations,^[^ ^225^ ^]^ rotational axisymmetric configurations,^[^ ^226^ ^]^ kirigami‐inspired rotational mechanisms,^[^ ^227^, ^228^, ^229^ ^]^ origami‐inspired rotational mechanisms,^[^ ^230^ ^]^ tensegrity lattices,^[^ ^231^ ^]^ and periodic assemblies.^[^ ^232^, ^233^ ^]^

**Figure 9:**
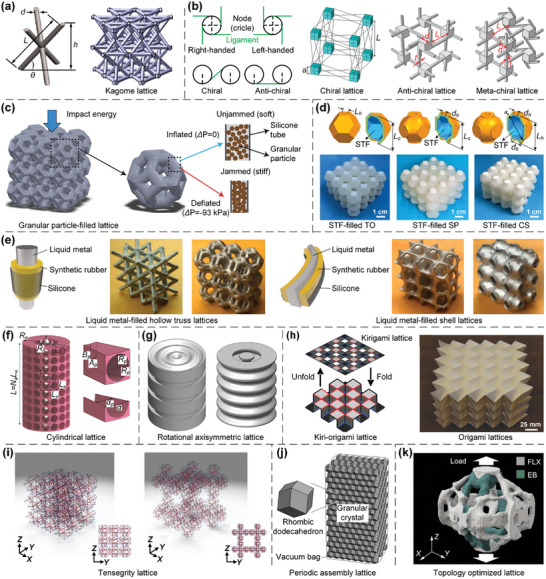
Design principles of various other forms of lattice metamaterials. a) 3D Kagome truss lattices. Reproduced with permission.^[^ ^220^ ^]^ Copyright 2023, Springer. b) 3D chiral, anti‐chiral, and meta‐chiral truss lattices. Reproduced with permission.^[^ ^234^ ^]^ Copyright 2016, Wiley. Reproduced with permission.^[^ ^235^ ^]^ Copyright 2018, Wiley. Reproduced with permission.^[^ ^236^ ^]^ Copyright 2018, Springer. c) Hollow tube lattices with granular particles filled inside. Reproduced with permission.^[^ ^223^ ^]^ Copyright 2019, Elsevier. d) Closed‐cell truncated octahedron (TO), Schwarz P (SP), and connected sphere (CS) lattices with STFs filled inside. Reproduced with permission.^[^ ^224^ ^]^ Copyright 2023, ACS. e) BCC hollow truss, Kelvin hollow truss, D shell, and P shell lattices with liquid metals filled inside. Reproduced with permission.^[^ ^239^ ^]^ Copyright 2022, Elsevier. f) Cylindrical lattices. Reproduced with permission.^[^ ^241^ ^]^ Copyright 2016, Elsevier. g) Rotational axisymmetric lattices. Reproduced with permission.^[^ ^226^ ^]^ Copyright 2020, Elsevier. h) Kirigami, kiri‐origami, and origami lattices. Reproduced with permission.^[^ ^228^ ^]^ Copyright 2022, Wiley. Reproduced with permission.^[^ ^230^ ^]^ Copyright 2019, Wiley. i) Tensegrity lattices. Reproduced with permission.^[^ ^231^ ^]^ Copyright 2019, Elsevier. j) Periodic assembly lattices. Reproduced with permission.^[^ ^242^ ^]^ Copyright 2024, Elsevier. k) Topologically optimized lattices. Reproduced with permission.^[^ ^24^ ^]^ Copyright 2023, AAAS.

More specifically, Kagome lattice, which takes the name from Japanese basket‐weaving patterns^[^ ^219^, ^220^ ^]^ (**Figure** **9**a), is an appealing structure to investigate novel physical phenomena. Chiral and anti‐chiral lattices are composed of nodes with attached ligaments, and their nodes are connected on either opposite sides or the same side of ligaments. Chiral lattices, which are either right‐handed or left‐handed, cannot be superimposed onto their mirror images by rotations and translations. In contrast, anti‐chiral lattices are racemic and possess an equal amount of right‐hand and left‐hand fundamental units. Besides, if some nodes of a lattice are attached to the same side of ligaments (such as in chiral lattices), while other nodes are attached to the opposite side (such as in anti‐chiral lattices), the lattice is named as meta‐chiral lattice^[^ ^221^, ^234^, ^235^, ^236^ ^]^ (Figure 9b). Filling lattices are typically hollow‐truss or closed‐cell lattices filled with granular particles, shear thickening fluids (STFs), liquid metals, etc. Using architected hollow‐truss lattices filled with granular particles, the jamming phase transition of granular particles can be tuned by actively controlling the pressure level within lattices to induce tailorable energy absorption and recoverable deformations^[^ ^223^ ^]^ (Figure 9c). Besides, 3D closed‐cell lattices filled with STFs offer low elastic moduli under quasi‐static conditions and superior energy absorption capabilities under dynamic impacts^[^ ^224^, ^237^, ^238^ ^]^ (Figure 9d). Additionally, hollow truss and shell lattices filled with liquid metals exhibit superior compressive strength, fracture toughness, damage recoverability, and reusable energy absorption^[^ ^239^, ^240^ ^]^ (Figure 9e). Furthermore, cylindrical and rotational axisymmetric lattices can be devised by the circular pattern^[^ ^241^ ^]^ (Figure 9f) and axisymmetric rotation^[^ ^226^ ^]^ (Figure 9g) of 2D configurations to obtain transversely isotropic performance, respectively. Another important class of lattice metamaterials are kirigami‐ and origami‐based lattices, which can be further categorized into three sub‐classes: kirigami‐based cutting lattices, origami‐based folding lattices, and kiri‐origami‐based cutting‐and‐folding hybrid lattices^[^ ^107^, ^108^, ^228^, ^230^ ^]^ (Figure 9h). The terms ‘kirigami’ and ‘origami’ originate from the Japanese words denoting their fabrication processes via paper cutting and folding, respectively. Therefore, kirigami‐ and origami‐based lattices are not AM techniques‐enabled lattices and will not be discussed in detail in this review. More comprehensive discussions on kirigami‐ and origami‐based lattices can be referred to in more detailed review articles.^[^ ^104^, ^105^, ^106^ ^]^ Moreover, tensegrity (derived from tensional integrity) lattices comprise discontinuous compressive members that are only connected via a continuous network of tensile members, thereby posing a stable volume in space with an internal balance of prestress. By appropriately tuning their prestress levels, tensegrity lattices can offer tuneable effective elastic properties, phononic bandgaps, and other mechanical/physical properties^[^ ^231^ ^]^ (Figure 9i). Furthermore, periodic assembly lattices exhibit plenty of mechanisms, such as nonlinear deformations, shear‐induced dilatancy, micro‐buckling, and crystal plasticity^[^ ^242^ ^]^ (Figure 9j). Topology optimization, as opposed to presetting the topology of microarchitectures as plate, shell, or truss, offers a more freeform design strategy for multi‐physical lattice metamaterials^[^ ^24^ ^]^ (Figure 9k).

## 3 Structure‐Mechanism‐Property Relationships of Lattice Metamaterials

The properties of lattice metamaterials are not only affected by their constitutive materials, but also by the spatial distribution of voids and solids. Internal microarchitectures that determine the mechanical properties of lattice metamaterials can also greatly affect their physical properties, including acoustic, EM/optical, thermal, and other properties. Through appropriate microarchitecture designs, the property space of lattice metamaterials can be substantially expanded, allowing for the achievement of numerous unprecedented properties, such as non‐positive equivalent parameters from constituents with positive parameters, chiral from achiral, anisotropic from isotropic, nonlinear from linear, etc. Researchers have thus been continuously working to uncover the relationships between the structural geometries/configurations and mechanical/physical properties of lattice metamaterials. Herein, we predominantly focus on the enhancement of the mechanical/physical properties of cellular architected lattices enabled by the design of internal microarchitectures, instead of that enabled by the chemical compositions of constitutive materials.

### 3.1 Mechanical Properties

This section reviews the relationships between the structural geometries/configurations and mechanical properties of lattice metamaterials. The mechanical properties considered in this review are classified as exceptional mechanical properties, non‐positive equivalent parameters, anomalous deformation mechanisms, and others. Specifically, exceptional mechanical properties refer to ultra‐lightweight, ultra‐stiff, ultra‐strong, (an)isotropic, pentamode properties, etc. Non‐positive equivalent parameters include non‐positive Poisson's ratios, non‐positive stiffnesses and non‐positive incremental stiffnesses, non‐positive CTEs, etc. Anomalous deformation mechanisms include torsion under axial tensile/compressive loads, lateral expansion/contraction and longitudinal extension/shrinkage under torsional loads, non‐reciprocity, etc.

#### 3.1.1 Exceptional Mechanical Properties

**Figure 10:**
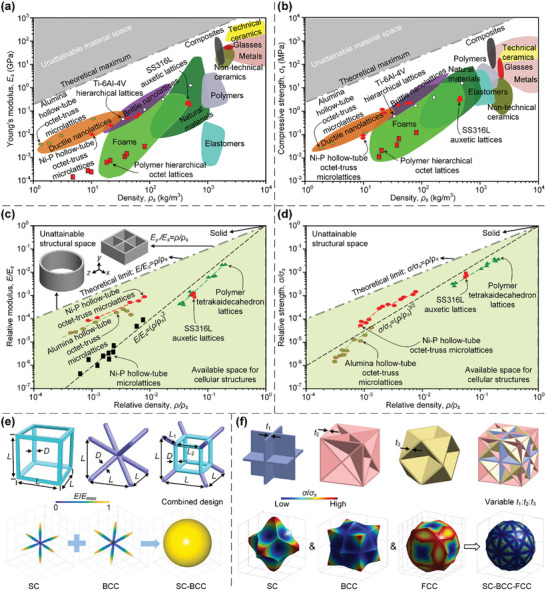
Plots of the Young's modulus and compressive strength versus density of solid constitutive materials, and the direction‐dependent relative Young's moduli and strengths of periodic lattice metamaterials. Experimentally measured a) Young's modulus and b) compressive strength versus density for existing solid constitutive materials. Reproduced with permission.^[^ ^244^ ^]^ Copyright 2021, Elsevier. Experimentally measured c) relative Young's modulus and d) relative compressive strength as a function of the relative density of lattices. Reproduced with permission.^[^ ^244^ ^]^ Copyright 2021, Elsevier. e) The direction‐dependent Young's moduli of SC, BCC, and SC‐BCC combined truss lattices, in which SC and BCC lattices possess extreme anisotropic elasticity, while SC‐BCC combined lattice exhibits isotropic elasticity. Reproduced with permission.^[^ ^252^ ^]^ Copyright 2022, Elsevier. f) The direction‐dependent uniaxial strengths of SC, BCC, FCC, and SC‐BCC‐FCC combined plate lattices, in which the optimal isotropic state in strength is realized by combining elementary lattices with variable plate thicknesses. Reproduced with permission.^[^ ^253^ ^]^ Copyright 2021, Elsevier.

The homogenized mechanical properties (including density *ρ*, Young's modulus *E*, strength *σ*, etc.) of lattice metamaterials are determined by both internal microarchitectures (i.e., the spatial distribution of voids and solids with single or multiple materials) and the mechanical properties of their constitutive materials (including density *ρ*_*s*_, Young's modulus *E_s_*, strength *σ*_*s*_, etc.). As a key indicator of stiffness, the effective Young's modulus *E* quantifies the ability of a lattice to resist deformations under external loads. The relative density *ρ*/*ρ*_*s*_ and relative Young's modulus *E*/*E_s_* of the lattice are dimensionless parameters that are determined by internal microarchitectures and independent on constitutive materials. In contrast to stiffness, the effective strength *σ* of the lattice is related to failure modes, and the relative strength *σ*/*σ*_*s*_ is a dimensionless parameter that is determined by both internal microarchitectures and the mechanical properties of constitutive materials (such as buckling strength *σ*_*bs*_, yield strength *σ*_*ys*_, ultimate strength *σ*_*us*_, and brittle fracture strength *σ*_*fs*_). The plots of *E_s_* and *σ*_*s*_ versus *ρ*_*s*_ for various solid constitutive materials are shown in **Figure** **10**a,b, and the values of *E_s_*/*ρ*_*s*_ and *σ*_*s*_/*ρ*_*s*_ are used to evaluate the specific stiffness and strength of solid constituents.^[^ ^119^, ^243^, ^244^ ^]^ Specifically, ultra‐lightweight lattices are defined as those with *ρ* < 10 kg/m^3^ or *ρ*/*ρ*_*s*_ < 0.1%, and are mainly formed by micro/nano fabrication.^[^ ^32^, ^34^ ^]^

The relative Young's modulus *E*/*E_s_*, relative strength *σ*/*σ*_*s*_, and relative density *ρ*/*ρ*_*s*_ of a lattice are related through a positive power law relationship according to the Gibson‐Ashby model:^[^ ^5^, ^6^, ^245^ ^]^

(1) $$\frac{E}{E_{s}}=C_{E}{\left(\frac{\rho }{\rho_{s}}\right)}^{n_{E}},\;\frac{\sigma }{\sigma_{s}}=C_{\sigma }{\left(\frac{\rho }{\rho_{s}}\right)}^{n_{\sigma }}$$

where *C_E_* and *C*_σ_ are scaling coefficients, *n_E_* and *n*_σ_ are exponential powers. The powers *n_E_* and *n*_σ_ are related to the deformation modes of lattices, ranging from bending‐dominated (*n_E_* ≥ 2, or *n*_σ_ ≥ 3/2) to stretching‐dominated modes (*n_E_* ≈ 1, or *n*_σ_ ≈ 1). The use of this model is referenced to the Maxwell strut connectivity number *m*:^[^ ^113^, ^114^, ^115^ ^]^

(2) m=b−3j+6

where *b* and *j* denote the number of struts and nodes of the lattice, respectively, and *m* is utilized to decide if the lattice is stretching‐dominated (*m*≥ 0) or bending‐dominated (*m*<0). The plots of *E*/*E_s_* and *σ*/*σ*_*s*_ versus *ρ*/*ρ*_*s*_ for several representative lattices are shown in Figure 10c,d. The theoretical Voigt upper bounds of Young's modulus and strength are *E*/*E_s_* = *ρ*/*ρ*_*s*_ and *σ*/*σ*_*s*_ = *ρ*/*ρ*_*s*_ for all lattices,^[^ ^15^, ^246^ ^]^ and they are represented by the out‐of‐plane relative stiffness and strength of 2D lattices with stretching‐dominated deformation behaviors. Rational design of microarchitectures and selection of appropriate geometric parameters allow the lattices to operate within the space below Voigt upper bounds. Gibson‐Ashby model, which is based on Euler‐Bernoulli beam theory,^[^ ^247^ ^]^ only considers axial and bending deformations. In comparison, Timoshenko‐Ehrenfest beam theory^[^ ^248^ ^]^ considers the axial, shear, and bending deformations of beams simultaneously, thereby resulting in more accurate predictions of their mechanical properties.^[^ ^249^, ^250^ ^]^

Herein, the direction‐dependent stiffness and strength are used to illustrate the anisotropy of lattice metamaterials. For instance, hexagonal and triangular honeycombs possess isotropic elasticity, in which hexagonal honeycombs exhibit much lower in‐plane stiffness than triangular honeycombs with identical length (*l*) and cell wall thickness (*t*). In comparison, square honeycombs are extremely anisotropic, and their in‐plane stiffness lies between that of triangular honeycombs and that of hexagonal honeycombs. Square honeycombs are stiffer in some directions but significantly weaker in other directions, representing a transition from stretching‐dominated to bending‐dominated deformation modes.^[^ ^247^ ^]^ The relative stiffness of 3D lattices is a function of 3D spatial directions, and their direction‐dependent behavior is like crystallographic orientation.^[^ ^117^, ^251^ ^]^ As illustrated by the effective Young's modulus surfaces within spatial coordinates in Figure 10e, SC and BCC truss lattices possess extreme anisotropic elasticity, whereas they can be combined with appropriate volume ratios to achieve isotropic elasticity.^[^ ^252^ ^]^ The value *E*_min_/*E*_max_ is utilized to define the anisotropic ratio of Young's modulus, in which *E*_min _/*E*_max _ = 1 represents an elastically isotropic lattice, whereas *E*_min _/*E*_max _ ≈ 0 represents an extremely anisotropic lattice. Similarly, *σ*_min _/*σ*_max _ is used to define the anisotropic ratio of strength. For example, the direction‐dependent uniaxial strengths of SC, BCC, FCC, and SC‐BCC‐FCC combined plate lattices are shown in Figure 10f, in which the optimal isotropic state in strength can be realized by combining elementary lattices with variable plate thicknesses.^[^ ^253^ ^]^ Typically, it is unreasonable to compare the highest stiffness or strength of a lattice with the weakest stiffness or strength of another. Instead, a specific evaluation standard should be determined, such as reaching a certain stiffness along the most rigid direction or achieving a certain strength along the strongest direction, or reaching an overall threshold of stiffness/strength, according to actual engineering conditions.

The structural design forms of ultra‐stiff lattices include truss lattices,^[^ ^34^ ^]^ hollow tube lattices,^[^ ^33^ ^]^ plate lattices,^[^ ^10^ ^]^ shell lattices,^[^ ^13^ ^]^ and hybrid lattices.^[^ ^157^ ^]^ Their stiffness can be enhanced by adjusting nodal connectivity to switch deformation modes from bending‐dominated to stretching‐dominated,^[^ ^34^ ^]^ replacing solid components with hollow tubular components,^[^ ^33^ ^]^ adopting hierarchical designs to increase the moment of inertia and stability under moderate deformations,^[^ ^20^ ^]^ etc. On the other hand, internal microarchitectures can cause the failure modes of lattice metamaterials to be different from those of their solid constituents. For example, reducing wall thickness or relative density can cause completely irrecoverable ceramic nanolattices to transform into partially or fully recoverable.^[^ ^33^ ^]^ Similarly, introducing hierarchical microarchitectures can significantly improve material utilization and endow a structure with various enhanced mechanical properties.^[^ ^20^ ^]^ The introduction of composite bars/struts with dual/multiple constitutive materials can enable the realization of ultra‐high recoverability, ultra‐high deformation ratios, and ultra‐high strength.^[^ ^123^ ^]^ Additionally, size effects achieved by reducing the characteristic sizes of structural components can result in substantially smaller lattices with significantly higher stiffness and strength.^[^ ^254^, ^255^, ^256^ ^]^ Other exceptional properties, such as ultra‐high ductility,^[^ ^257^ ^]^ ultra‐high fracture toughness,^[^ ^258^, ^259^, ^260^ ^]^ ultra‐high fatigue resistance,^[^ ^261^ ^]^ ultra‐high impact resilience,^[^ ^262^ ^]^ and pentamode properties with finite bulk moduli *K* but ultra‐low shear moduli *G* (*G*/*K*→0),^[^ ^35^, ^36^, ^37^ ^]^ have also been extensively explored in studies on lattice metamaterials.

#### 3.1.2 Non‐Positive Equivalent Parameters

**Figure 11:**
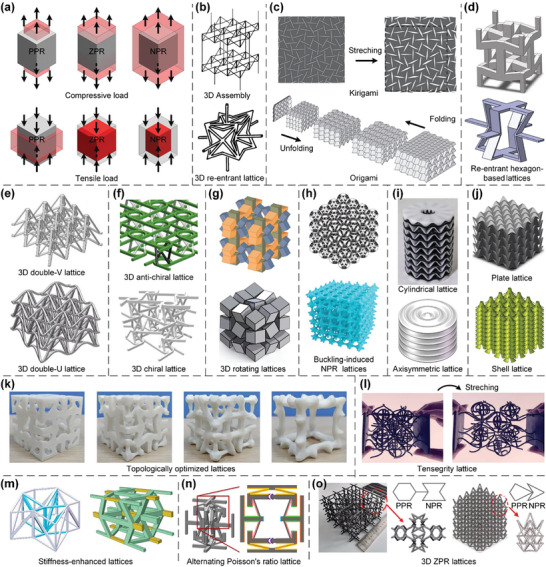
Representative mechanisms and 3D lattice metamaterials with negative or zero Poisson's ratios. a) The deformation processes of PPR, ZPR, and NPR isotropic lattices under compressive and tensile loads. The lattice in grey represents the initial state, while that in red represents the deformed state. b) 3D hierarchical assembly NPR lattices based on rod, hinge, and spring components, and 3D re‐entrant NPR lattices. Reproduced with permission.^[^ ^276^ ^]^ Copyright 1985, Springer. Reproduced with permission.^[^ ^278^ ^]^ Copyright 1987, AAAS. c) NPR kirigami and origami lattices. Reproduced with permission.^[^ ^286^ ^]^ Copyright 2016, Wiley. Reproduced with permission.^[^ ^287^ ^]^ Copyright 2013, PNAS. d) Re‐entrant/concave hexagonal 3D NPR lattices. Reproduced with permission.^[^ ^288^ ^]^ Copyright 2015, Elsevier. Reproduced with permission.^[^ ^289^ ^]^ Copyright 2021, Elsevier. e) Re‐entrant/concave double‐V straight‐beam and double‐U curved‐beam 3D NPR lattices. Reproduced with permission.^[^ ^290^ ^]^ Copyright 2018, Elsevier. Reproduced with permission.^[^ ^119^ ^]^ Copyright 2019, Elsevier. f) Chiral/anti‐chiral 3D NPR lattices. Reproduced with permission.^[^ ^293^ ^]^ Copyright 2018, Elsevier. g) Rotational 3D NPR lattices. Reproduced with permission.^[^ ^294^ ^]^ Copyright 2012, Wiley. Reproduced with permission.^[^ ^295^ ^]^ Copyright 2018, MSP. h) Buckling‐induced 3D NPR lattices. Reproduced with permission.^[^ ^296^ ^]^ Copyright 2013, Wiley. Reproduced with permission.^[^ ^297^ ^]^ Copyright 2021, Elsevier. i) Cylindrical and rotational axisymmetric NPR lattices. Reproduced with permission.^[^ ^298^ ^]^ Copyright 2016, Elsevier. Reproduced with permission.^[^ ^226^ ^]^ Copyright 2020, Elsevier. j) 3D double arrow‐head NPR plate and shell lattices. Reproduced with permission.^[^ ^299^ ^]^ Copyright 2022, Elsevier. Reproduced with permission.^[^ ^300^ ^]^ Copyright 2022, Elsevier. k) Topologically optimized 3D NPR lattices. Reproduced with permission.^[^ ^301^ ^]^ Copyright 2019, Elsevier. l) 3D re‐entrant tensegrity NPR lattices. Reproduced with permission.^[^ ^40^ ^]^ Copyright 2021, AAAS. m) Stiffness‐enhanced 3D NPR lattices. Reproduced with permission.^[^ ^304^ ^]^ Copyright 2021, Elsevier. Reproduced with permission.^[^ ^305^ ^]^ Copyright 2017, Elsevier. n) Alternating Poisson's ratio lattices. Reproduced with permission.^[^ ^306^ ^]^ Copyright 2022, Springer. o) 3D ZPR lattices by connecting convex PPR and concave NPR hexagonal/quadrilateral unit cells in parallel. Reproduced with permission.^[^ ^318^ ^]^ Copyright 2020, Elsevier. Reproduced with permission.^[^ ^319^ ^]^ Copyright 2021, Elsevier.

Poisson's ratio *ν_s_*, the negative ratio of transverse to longitudinal strains, was first proposed to reflect the transverse deformation of a material under longitudinal loads^[^ ^263^, ^264^, ^265^ ^]^ (**Figure** **11**a). Most materials feature positive Poisson's ratios (PPRs),^[^ ^266^ ^]^ such as 0.10 to 0.30 for composites and technical ceramics, 0.25 to 0.44 for metals, and 0.30 to 0.47 for polymers and elastomers. In recent decades, some natural materials, such as pyrite, cancellous bone, skin, cubic lattice metals, and zeolite, were found to exhibit NPRs.^[^ ^267^, ^268^, ^269^, ^270^, ^271^, ^272^ ^]^ For anisotropic lattices, the Poisson's ratio *ν* is direction‐dependent and is correlated to the Young's modulus *E* according to the reciprocal theorem of static mechanical systems.^[^ ^226^ ^]^ The design of artificial NPR lattice metamaterials was initially based on concave mechanisms, including 2D re‐entrant/concave hexagonal honeycombs,^[^ ^245^ ^]^ 2D re‐entrant/concave quadrilateral lattices,^[^ ^273^ ^]^ 2D concave star‐shaped lattices,^[^ ^274^, ^275^ ^]^ 2D concave hexagonal and 3D hierarchical assembly lattices based on rod, hinge, and spring components^[^ ^276^, ^277^ ^]^ and 3D re‐entrant lattices^[^ ^278^ ^]^ (Figure 11b). Subsequently, other forms of NPR metamaterials were also developed, including 2D chiral and anti‐chiral lattices,^[^ ^275^, ^279^ ^]^ perforated 2D sheets under instability‐induced large deformations,^[^ ^280^ ^]^ and 2D lattices with rotational mechanisms of hinged rigid elements^[^ ^281^ ^]^ or translational mechanisms of interlocking assemblies.^[^ ^232^, ^233^ ^]^ Moreover, 2D NPR metamaterials with curved‐beam configurations,^[^ ^282^ ^]^ hierarchical honeycombs,^[^ ^283^ ^]^ topologically optimized microarchitectures,^[^ ^284^ ^]^ and bio‐inspired designs,^[^ ^285^ ^]^ and NPR lattices based on kirigami^[^ ^286^ ^]^ and origami^[^ ^287^ ^]^ (Figure 11c) have also been extensively studied. However, owing to the limitations of manufacturing processes, these studies predominantly focused on the design of 2D NPR metamaterials.

Recently, developments in AM techniques have facilitated the design of numerous 3D NPR lattice metamaterials, including 3D re‐entrant/concave lattices^[^ ^288^, ^289^ ^]^ (Figure 11d), 3D double‐V straight‐beam and double‐U curved‐beam lattices^[^ ^119^, ^290^ ^]^ (Figure 11e), 3D hierarchical lattices,^[^ ^291^, ^292^ ^]^ 3D chiral and anti‐chiral lattices^[^ ^293^ ^]^ (Figure 11f), 3D lattices with hinged rotating tessellations^[^ ^294^, ^295^ ^]^ (Figure 11g), 3D lattices with buckling‐induced NPR deformation mechanisms^[^ ^296^, ^297^ ^]^ (Figure 11h), 3D lattices with tubular/cylindrical^[^ ^298^ ^]^ and rotational axisymmetric^[^ ^226^ ^]^ configurations (Figure 11i), 3D double arrow‐head plate and shell lattices^[^ ^299^, ^300^ ^]^ (Figure 11j), 3D lattices with topologically optimized microarchitectures^[^ ^301^ ^]^ (Figure 11k), 3D re‐entrant tensegrity lattices^[^ ^40^ ^]^ (Figure 11l), and 3D lattices with out‐of‐plane NPR deformation mechanisms.^[^ ^302^ ^]^ The addition of connecting rods in bending‐dominated concave NPR lattices can significantly enhance their stiffness and strength^[^ ^303^, ^304^, ^305^ ^]^ (Figure 11m). Besides, the deformation modes of 2D/3D lattices can be repeatedly changed through variable stiffness distribution and sequential contact guidance, thereby enabling equivalent Poisson's ratios which alternate positive and negative changes under unidirectional loadings^[^ ^306^, ^307^ ^]^ (Figure 11n). Furthermore, these lattices can be fabricated using various manufacturing techniques, including single‐material AM,^[^ ^288^ ^]^ dual/multi‐material AM,^[^ ^308^, ^309^ ^]^ micro/nano processing,^[^ ^310^ ^]^ metal casting with wax,^[^ ^311^ ^]^ interlocking assembly with welding/bonding,^[^ ^290^ ^]^ etc.

In addition to NPR metamaterials, ZPR architected materials also attract increasing attention. Rectangular and square honeycombs and some parallelogram and parallelogram‐like structures represent the simplest ZPR metamaterials. Additionally, ZPR structures can be formed by replacing the edge of square honeycombs with Diamond (D) or helical beam to realize zero lateral deformations under large tensile strains.^[^ ^312^, ^313^ ^]^ Besides, quasi‐parallelogram ZPR honeycombs can be formed by removing some members from parallelogram honeycombs.^[^ ^314^ ^]^ Another typical ZPR mechanism involves alternating and paralleling NPR and PPR lattices to compensate for lateral expansion/contraction, including semi‐concave hexagonal honeycombs formed by paralleling concave and convex configurations,^[^ ^315^, ^316^, ^317^ ^]^ 3D AuxHex lattices comprising PPR hexagonal and NPR re‐entrant components in parallel,^[^ ^318^, ^319^ ^]^ and combined ZPR lattices formed by alternately parallelizing concave and convex double‐V elements^[^ ^320^ ^]^ (Figure 11o). Furthermore, flexible and rigid components (based on geometric parameters or the elastic moduli of constitutive materials) were alternately connected in series to achieve zero lateral deformations under large axial strains. For example, ZPR honeycombs can be formed by concatenating transverse bars with concave and convex hexagonal elements to maintain ZPR under large strains.^[^ ^321^, ^322^ ^]^ Overall, current studies on ZPR metamaterials have predominantly focused on 2D metamaterials, and the design principles and deformation mechanisms of metamaterials that maintain ZPR under large axial strains remain less explored.

**Figure 12:**
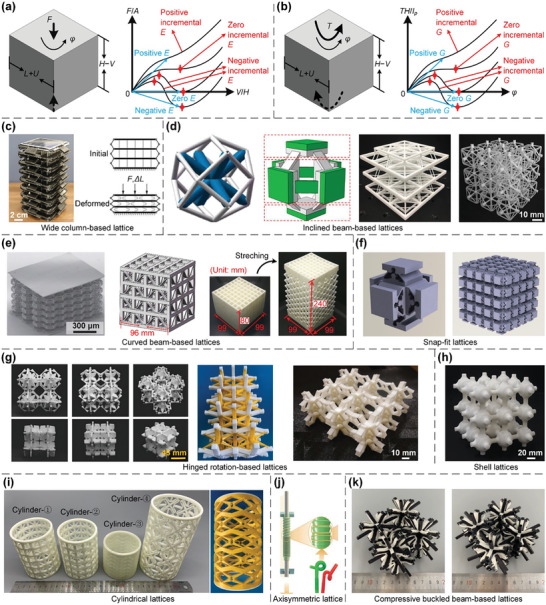
Representative multi‐stable and self‐recoverable snapping mechanisms and 3D lattice metamaterials with negative or zero incremental stiffness. a) Illustration of positive, zero, and negative Young's modulus *E* and incremental Young's modulus *E*_*in*_ of a lattice under an axial load. b) Illustration of positive, zero, and negative shear modulus *G* and incremental shear modulus *G*_*in*_ of a lattice under a torsional load. c) Self‐recoverable 3D lattices with wide hyperelastic columns as snapping elements. Reproduced with permission.^[^ ^324^ ^]^ Copyright 2021, Wiley. d) Multi‐stable and self‐recoverable 3D lattices with inclined beams as snapping elements. Reproduced with permission.^[^ ^325^ ^]^ Copyright 2015, Wiley. Reproduced with permission.^[^ ^326^ ^]^ Copyright 2024, Elsevier. Reproduced with permission.^[^ ^327^ ^]^ Copyright 2018, Elsevier. Reproduced with permission.^[^ ^328^ ^]^ Copyright 2019, Elsevier. e) Self‐recoverable and multi‐stable 3D lattices with original curved beams as snapping elements. Reproduced with permission.^[^ ^331^ ^]^ Copyright 2016, Wiley. Reproduced with permission.^[^ ^332^ ^]^ Copyright 2019, Springer. Reproduced with permission.^[^ ^333^ ^]^ Copyright 2018, Elsevier. f) Multidirectional snapping 3D lattices with snap‐fit interconnections. Reproduced with permission.^[^ ^337^ ^]^ Copyright 2018, Wiley. g) Multi‐stable shape reconfigurable 3D lattices based on hinged rotation mechanisms. Reproduced with permission.^[^ ^338^ ^]^ Copyright 2016, Wiley. Reproduced with permission.^[^ ^339^ ^]^ Copyright 2022, Elsevier. Reproduced with permission.^[^ ^340^ ^]^ Copyright 2023, Wiley. h) Multi‐stable perforated shellular lattices. Reproduced with permission.^[^ ^342^ ^]^ Copyright 2021, Wiley. i) Multi‐stable and self‐recoverable cylindrical lattices. Reproduced with permission.^[^ ^343^ ^]^ Copyright 2020, Elsevier. Reproduced with permission.^[^ ^339^ ^]^ Copyright 2022, Elsevier. j) Multi‐stable rotational axisymmetric lattices. Reproduced with permission.^[^ ^344^ ^]^ Copyright 2019, Wiley. k) Multi‐stable 3D lattices with compressively buckled beams as snapping elements. Reproduced with permission.^[^ ^346^ ^]^ Copyright 2020, Elsevier.

Elastic instability is a common phenomenon in nature, such as the rapid shutting of a Venus flytrap when it is touched and the curling of wrist straps when they are slapped on a wrist. These phenomena are typically associated with negative incremental modulus/stiffness that leads to snapping behaviors. Considering nonlinear large deformations, the equivalent Young's modulus *E* is defined based on original and specific final states under an axial load *F*, regardless of the deformation process, and can be divided into positive, zero, and negative tensile or compressive *E*, characterized by the longitudinal stress (*F*/*A*)‐longitudinal strain (*V*/*H*) curve (**Figure** **12**a). The incremental Young's modulus *E_in_* is defined as the slope of the stress‐strain curve at any given point,^[^ ^41^, ^42^ ^]^ which can be classified into positive, zero, and negative *E_in_*. When the external force becomes a torque *T*, the lattice will rotate, and its mechanical characteristic is the shear stress (*TH*/*I_P_*)‐twist degree (*φ*) curve (Figure 12b). The positive, zero, and negative equivalent shear modulus *G* and incremental shear modulus *G_in_* can be classified in an analogous manner to parameters *E* and *E_in_*. So far, various 2D/3D lattice metamaterials have been devised to exhibit snap‐through and snap‐back behaviors by the chain/series effects of discrete and periodic unstable snapping units.^[^ ^323^ ^]^ For example, self‐recoverable 3D lattices can be formed by harnessing the snapping behavior of wide hyperelastic columns^[^ ^324^ ^]^ (Figure 12c). Besides, multi‐stable lattices with inclined beams as bistable snapping elements were reported, which exhibited fully recoverable elastic buckling deformations and could store elastic strain energies to provide protection by absorbing impacts.^[^ ^325^, ^326^ ^]^ Compared with the compressive state, the tensile state of these lattices exhibited significantly reduced peak stresses under impact loadings (Figure 12d). Similarly, quadrilateral and cubic negative stiffness lattices were devised based on inclined snapping beams, which exhibited superior energy absorption capabilities under impact loads in one and three principal directions^[^ ^327^, ^328^ ^]^ (Figure 12d). Lattice metamaterials with multi‐step deformation modes and multiple compressive plateau stresses can also be formed by combining inclined snapping beams with Euler buckling mechanisms.^[^ ^329^ ^]^ Moreover, the effects of wall thickness and arch height on the mechanical responses of original curved beams were comprehensively studied, based on which 2D periodic lattices that could generate self‐recoverable large deformations under axial tensile loads were reported.^[^ ^330^ ^]^ Furthermore, self‐restoring lattices with enhanced energy absorption capabilities under compressive loads were devised based on original curved beams and regular hexagonal frameworks, and their stress‐strain hysteresis effects and energy dissipation mechanisms caused by chain/series effects were studied^[^ ^331^ ^]^ (Figure 12e). The other related designs include ZPR multi‐stable 3D lattices with original curved beams as snapping elements^[^ ^332^, ^333^ ^]^ (Figure 12e), multi‐stable lattices with determinable deformation sequences formed by changing the wall thickness and curve form of original curved beams in each layer,^[^ ^334^ ^]^ 1D, 2D, and 3D snapping metamaterials based on original curved beams,^[^ ^335^ ^]^ etc. In addition, the concept of phase transition was introduced into periodic porous lattices, and the fully recoverable deformation behaviors and energy absorption/dissipation mechanisms of 2D multi‐stable metamaterials based on curved snapping beams were investigated.^[^ ^336^ ^]^

Moreover, the multi‐stable mechanisms and energy dissipation performances of snap‐fit lattices were studied^[^ ^337^ ^]^ (Figure 12f). Based on hinged rotation mechanisms, 1D, 2D, and 3D multi‐stable shape reconfigurable metamaterials were devised to achieve enhanced strength and recoverable energy absorption^[^ ^338^ ^]^ (Figure 12g). Similarly, 3D snapping lattices were proposed based on the hinge rotation of triangular elements^[^ ^339^, ^340^ ^]^ (Figure 12g). Besides, a series of novel programmable and reusable energy‐absorbing metamaterials were devised by assembling flexible stretchable components and rigid cylinders or particles.^[^ ^341^ ^]^ The other related designs include multi‐stable perforated shellular lattices^[^ ^342^ ^]^ (Figure 12h), multi‐stable and self‐recoverable cylindrical lattices^[^ ^339^, ^343^ ^]^ (Figure 12i), 3D pixel metamaterials with superior mechanical properties, programmability, complex shape reconfigurability, and efficient and repeatable absorption of impact energy based on rotational axisymmetric shell elements^[^ ^344^ ^]^ (Figure 12j), 2D stretching‐dominated kirigami lattices with auxetic bi‐stability inspired by the geometric patterns of ancient building skins,^[^ ^345^ ^]^ dual negative parameter metamaterials with both NPRs and negative stiffness that combine multiple snapping mechanisms with planar topologies,^[^ ^42^ ^]^ etc. Besides, 1D, 2D, and 3D ZPR multi‐stable and 2D NPR bistable lattices were proposed based on compressively buckled beams^[^ ^346^, ^347^ ^]^ (Figure 12k), and a series of metamaterials with both adjustable Poisson's ratios and stiffness were subsequently devised.^[^ ^348^ ^]^ Other snapping lattices that have been formed include those with truncated cones,^[^ ^349^ ^]^ optimized topologies,^[^ ^350^ ^]^ tensegrity systems,^[^ ^351^ ^]^ spring and gear systems,^[^ ^352^ ^]^ self‐contact designs,^[^ ^353^, ^354^ ^]^ kirigami and origami lattices,^[^ ^355^, ^356^, ^357^ ^]^ and negative stiffness snapping mechanisms for shear,^[^ ^351^ ^]^ bending,^[^ ^358^ ^]^ torsional,^[^ ^358^ ^]^ pneumatic,^[^ ^359^, ^360^ ^]^ and hydraulic,^[^ ^361^, ^362^ ^]^ loads.

**Figure 13:**
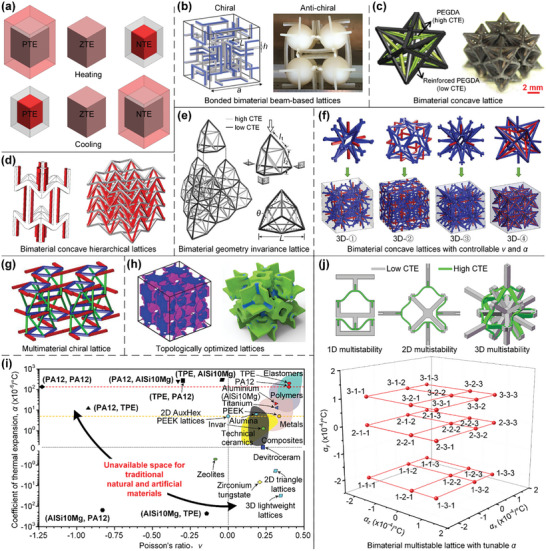
Representative mechanisms and 3D lattice metamaterials with negative or zero CTEs. a) The deformation processes of positive thermal expansion (PTE), zero thermal expansion (ZTE), and negative thermal expansion (NTE) isotropic lattices under heating and cooling conditions. The lattice in grey represents the initial state, while that in red represents the deformed state. b) Chiral/anti‐chiral 3D lattices consisting of bonded bi‐material beams. Reproduced with permission.^[^ ^371^ ^]^ Copyright 2017, Springer. Reproduced with permission.^[^ ^370^ ^]^ Copyright 2016, ACS. c) Bi‐material 3D concave lattices. Reproduced with permission.^[^ ^375^ ^]^ Copyright 2016, APS. d) Bi‐material concave hierarchical lattices. Reproduced with permission.^[^ ^376^ ^]^ Copyright 2018, IOP. Reproduced with permission.^[^ ^266^ ^]^ Copyright 2021, Elsevier. e) Bi‐material 3D lattices with spatial geometric invariance systems. Reproduced with permission.^[^ ^377^ ^]^ Copyright 2007, Elsevier. f) Four bi‐material 3D re‐entrant lattices with controllable positive, near zero, or negative Poisson's ratios and CTEs. Reproduced with permission.^[^ ^384^ ^]^ Copyright 2018, Elsevier. g) Tri‐material 3D chiral lattices with tuneable positive, near zero, or negative CTEs and Poisson's ratios. Reproduced with permission.^[^ ^386^ ^]^ Copyright 2020, IOP. h) Topologically optimized bi‐material 3D lattices with controllable positive, near zero, or negative Poisson's ratios and CTEs. Reproduced with permission.^[^ ^387^ ^]^ Copyright 2021, TSP. Reproduced with permission.^[^ ^388^ ^]^ Copyright 2022, Elsevier. i) Experimental CTE versus Poisson's ratio for natural and artificial materials. Reproduced with permission.^[^ ^266^ ^]^ Copyright 2021, Elsevier. j) A multi‐stable ZPR mechanism to achieve adjustable positive/zero/negative CTEs for 1D, 2D, and 3D lattices based on compressively buckled beams. Reproduced with permission.^[^ ^346^ ^]^ Copyright 2020, Elsevier.

In engineering applications, the temperature variations of most materials lead to changes in object sizes, which are typically characterized by their effective CTEs (*α*_*s*_).^[^ ^363^ ^]^ Accordingly, the thermal expansion/contraction deformation behaviors of structures induced by temperature changes have been widely studied. Single‐phase materials generally possess positive CTEs and exhibit thermal expansion and cold contraction, some natural materials^[^ ^364^, ^365^, ^366^ ^]^ possess negative CTEs and exhibit thermal contraction and cold expansion, and some special materials possess zero CTEs and exhibit no dimensional changes within a certain range of temperature (**Figure** **13**a). Although the effective CTE of a single‐material porous structure is the same as its constitutive material, the CTE of a composite structure comprising two uniform solid phases and a void space is highly tuneable.^[^ ^367^, ^368^, ^369^ ^]^ Current studies have reported various deformation mechanisms that regulate the CTEs of structures. For example, a bonded bi‐material beam structure with negative thermal expansion was devised through local bending deformations based on different CTEs of the two different constitutive materials.^[^ ^367^ ^]^ Thereafter, a series of chiral/anti‐chiral 3D lattices consisting of bonded bi‐material beams were proposed to achieve negative effective CTEs^[^ ^369^, ^370^, ^371^, ^372^ ^]^ (Figure 13b). Moreover, negative CTE lattices have also been developed based on 2D/3D concave configurations,^[^ ^266^, ^373^, ^374^, ^375^, ^376^ ^]^ including bi‐material 2D concave and convex combined lattices with negative and positive thermal expansions along two perpendicular directions,^[^ ^373^ ^]^ bi‐material 2D/3D concave lattices with negative CTEs that consisted of two materials with different positive CTEs^[^ ^374^, ^375^ ^]^ (Figure 13c), and bi‐material 3D re‐entrant and double‐V hierarchical lattices with effective CTEs ranging from positive to zero and negative^[^ ^266^, ^376^ ^]^ (Figure 13d). Due to the different thermal deformation behaviors of two constitutive materials during temperature changes, the concave section of these lattices underwent a greater thermal expansion than other sections, resulting in a macroscopic equivalent shrinkage upon exposure to heating. Besides, by studying the effects of hinged and fixed connection methods on effective CTEs, bi‐material negative CTE lattices with planar and spatial geometry invariance systems were also devised^[^ ^377^ ^]^ (Figure 13e). The other design forms of negative CTE lattices include non‐bonded bi‐material beams with amplified thermal deformations,^[^ ^378^, ^379^ ^]^ non‐bonded kirigami bi‐material composites,^[^ ^380^ ^]^ origami lattices with elastic films added to surfaces and controllable connection/disconnection of creases,^[^ ^43^ ^]^ cylindrical configurations,^[^ ^381^ ^]^ optimized topologies,^[^ ^382^ ^]^ etc.

Moreover, the interaction mechanisms between thermal and mechanical properties are also studied in lattice metamaterials. For instance, four 2D/3D lattices based on bi‐material concave star‐shaped elements were devised to obtain controllable positive, near zero, or negative Poisson's ratios and CTEs simultaneously^[^ ^383^, ^384^ ^]^ (Figure 13f). Similarly, based on the combination of concave quadrilateral NPR elements and triangular geometrically invariant hinged negative thermal expansion elements, 2D hierarchical lattices with controllable positive, zero, or negative Poisson's ratios and CTEs were developed.^[^ ^385^ ^]^ Besides, 3D tri‐material chiral lattices with tuneable positive, near zero, or negative CTEs and Poisson's ratios were also reported^[^ ^386^ ^]^ (Figure 13g). The other design forms of lattice metamaterials with both NPRs and negative CTEs include 3D bi‐material lattices with optimized topologies^[^ ^387^, ^388^ ^]^ (Figure 13h), 2D/3D ceramic aerogel lattices that realized thermal insulation in extreme environments,^[^ ^44^ ^]^ etc. As a summary, the experimentally measured CTE versus Poisson's ratio plots of natural materials and artificial lattice materials are summarized in Figure 13i.^[^ ^266^ ^]^ Additionally, an interlocking assembly method and a multi‐stable ZPR mechanism were proposed to devise 1D, 2D, and 3D lattices with adjustable positive/negative/zero CTEs, based on Euler buckling beams that functioned as fully symmetric bistable elements^[^ ^346^ ^]^ (Figure 13j). Subsequently, an angle‐dependent transition between bistable NPR and multi‐stable ZPR was achieved using square frameworks embedded Euler buckling beams, and triple‐negative‐index (NPR, negative CTE, and negative incremental stiffness) lattices using hexagonal frameworks embedded Euler buckling beams were further devised.^[^ ^347^ ^]^ Other non‐positive equivalent parameters, such as negative area and volume compressibility with negative bulk modulus^[^ ^389^, ^390^ ^]^ and negative hydration expansion,^[^ ^391^, ^392^, ^393^ ^]^ have also been extensively studied.

#### 3.1.3 Anomalous Deformation Mechanisms

**Figure 14:**
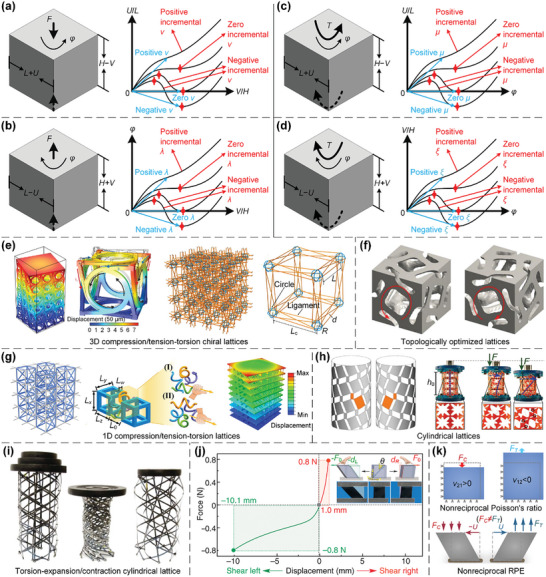
Representative lattice metamaterials with anomalous deformation mechanisms. a) Illustration of positive, zero, and negative Poisson's ratio *ν* and incremental Poisson's ratio *ν*_*in*_ of a lattice under an axial load. b) Illustration of positive, zero, and negative compression/tension‐torsion index *λ* and incremental index *λ*_*in*_ of a lattice under an axial load. c) Illustration of positive, zero, and negative torsion‐lateral expansion/contraction index *µ* and incremental index *µ*_*in*_ of a lattice under a torsional load. d) Illustration of positive, zero, and negative torsion‐vertical extension/shrinkage index and incremental index of a lattice under a torsional load. e) Compression/tension‐torsion chiral lattices which can twist under axial loads in all the three principal directions. Reproduced with permission.^[^ ^394^ ^]^ Copyright 2017, AAAS. Reproduced with permission.^[^ ^402^ ^]^ Copyright 2018, Elsevier. f) Topologically optimized compression/tension‐torsion chiral lattices with cubic symmetry. Reproduced with permission.^[^ ^396^ ^]^ Copyright 2019, Elsevier. g) 3D straight beam‐based, curved beam‐based, and truss‐plate hybrid lattices which can twist under an axial load in a specific principal direction. Reproduced with permission.^[^ ^403^ ^]^ Copyright 2019, Elsevier. Reproduced with permission.^[^ ^406^ ^]^ Copyright 2022, Wiley. Reproduced with permission.^[^ ^407^ ^]^ Copyright 2019, Springer. h) Cylindrical lattices with compression/tension‐torsion coupling effects. Reproduced with permission.^[^ ^47^ ^]^ Copyright 2018, AAAS. Reproduced with permission.^[^ ^48^ ^]^ Copyright 2021, Wiley. i) Cylindrical lattices with both torsion‐lateral expansion/contraction and torsion‐vertical extension/shrinkage coupling effects, which are devised based on spatial spiral rod systems. Reproduced with permission.^[^ ^47^ ^]^ Copyright 2018, AAAS. j) A uniform composite material based on direction‐dependent buckling of embedded nanofillers with static non‐reciprocity. Reproduced with permission.^[^ ^414^ ^]^ Copyright 2023, AAAS. k) Illustration of non‐reciprocal Poisson's ratio and RPE. Reproduced with permission.^[^ ^38^ ^]^ Copyright 2023, Springer. Reproduced with permission.^[^ ^415^ ^]^ Copyright 2024, AAAS.

According to the principles of conventional continuum mechanics, Cauchy elastic materials cannot reflect some coupling deformation effects, such as the inability to twist under axial tensile or compressive loads, the inability to extend/shrink longitudinally or expand/contract laterally under torsional loads, etc. However, lattice metamaterials can attain some representative anomalous deformation mechanisms through appropriate design of internal microarchitectures. Except for the positive, zero, and negative tensile or compressive *E* and *E_in_* under an axial load *F* for nonlinear large deformations (Figure 12a), lattice metamaterials can also expand or contract laterally under a longitudinal tensile/compressive load *F*, and their mechanical behaviors are characterized by the transverse (*U*/*L*)‐longitudinal strain (*V*/*H*) curve, equivalent Poisson's ratio *ν*, and incremental Poisson's ratio *ν_in_* (**Figure** **14**a). The equivalent Poisson's ratio *ν* is defined as the negative ratio of lateral and longitudinal strains based on the original and specific final states, which can be classified into positive, zero, and negative *ν*. The incremental Poisson's ratio *ν_in_* is defined as the slope of the lateral strain‐longitudinal strain curve, which can also be classified into positive, zero, and negative *ν_in_*. Another deformation mechanism under the longitudinal tensile or compressive load *F* is torsion, which can be characterized by the twist degree (*φ*)‐longitudinal strain (*V*/*H*) curve. According to the direction of rotation (the force and direction of rotation that meet the right‐hand criterion are positive), the compression/tension‐torsion index *λ* and incremental index *λ_in_* can be divided into positive, zero, and negative values, as shown in Figure 14b. The representative architected materials with these abnormal deformation mechanisms are called compression/tension‐torsion metamaterials.^[^ ^394^, ^395^, ^396^, ^397^, ^398^ ^]^ When the external force becomes a torque *T*, except for the positive, zero, and negative equivalent shear modulus *G* and incremental shear modulus *G_in_* (Figure 12b), the lattice can also expand or contract laterally under the torsional load *T*. This behavior can be mechanically characterized by the lateral strain (*U*/*L*)‐twist degree (*φ*) curve, the torsion‐lateral expansion/contraction index *µ*, and incremental index *µ_in_* (Figure 14c). The representative structured materials with these abnormal deformation mechanisms are called torsion‐lateral expansion/contraction metamaterials.^[^ ^47^, ^399^ ^]^ Simultaneously, the lattice will also elongate or shorten longitudinally under the torsional load *T*, which can be characterized by the longitudinal strain (*V*/*H*)‐twist degree (*φ*) curve, the torsion‐vertical extension/shrinkage index *ξ*, and incremental index *ξ_in_* (Figure 14d). The representative architected materials with these abnormal deformation mechanisms are called torsion‐vertical extension/shrinkage metamaterials.^[^ ^48^, ^400^, ^401^ ^]^ Overall, axial compression/tension‐vertical extension/shrinkage, compression/tension‐lateral expansion/contraction, compression/tension‐torsion, torsion‐twisting, torsion‐lateral expansion/contraction, and torsion‐vertical extension/shrinkage deformation mechanisms reflect special coupling relationships among the axial, lateral, and torsional/shear deformations of lattice metamaterials under axial or torsional/shear loads.

Current studies on compression/tension‐torsion coupling metamaterials began with the 3D chiral lattices that underwent torsion under axial loads.^[^ ^394^ ^]^ Henceforth, compression/tension‐torsion 3D chiral lattices which can twist under axial loads in all the three principal directions^[^ ^394^, ^402^ ^]^ (Figure 14e), topologically optimized 3D compression/tension‐torsion chiral lattices with cubic symmetry^[^ ^396^ ^]^ (Figure 14f), 3D straight beam‐based, curved beam‐based, and truss‐plate hybrid lattices that can twist under axial loads in a specific principal direction^[^ ^403^, ^404^, ^405^, ^406^, ^407^ ^]^ (Figure 14g), and cylindrical compression/tension‐torsion lattices^[^ ^47^, ^48^, ^408^, ^409^, ^410^ ^]^ (Figure 14h) have been extensively studied. Besides, 3D compression‐torsion lattices can also be formed based on kirigami and origami patterns.^[^ ^395^ ^]^ Based on the reciprocal theorem of static systems, the torsion‐vertical extension/shrinkage deformation mechanism corresponds to the compression/tension‐torsion mechanism. In addition, the basis of torsion‐vertical extension/shrinkage mechanism is the longitudinal dimensional change of lattices under shear loads.^[^ ^48^ ^]^ Tightly tied piano strings are shown to lengthen when twisted, since the torsion generates axial stresses perpendicular to the shear plane, on condition that the distance between two ends is fixed.^[^ ^400^ ^]^ This phenomenon is known as the Poynting effect, and the axial stress or deformation generated under torsional/shear loads are equivalent manifestations of this effect. On the other hand, another classical coupling effect, reverse Poynting effect (RPE), represents the torsional/shear deformation induced under an axial tensile or compressive load.^[^ ^401^ ^]^ Recently, a series of lattices with Poynting effect and RPE were devised based on ordered hollow elastic cylinders, which underwent normal elongation or contraction under torsional loads and nonlinear torsional deformations under axial compressive loads.^[^ ^48^ ^]^ Besides, lattice metamaterials with Poynting effect and RPE also play important roles in elastic wave propagation control, such as the mode conversion of longitudinal and torsional/shear waves.^[^ ^411^ ^]^ As the lateral deformation of lattices under axial loads are represented by Poisson's effect, the lateral deformation under torsional loads can be represented by the torsion‐lateral expansion/contraction effect. Recently, a series of torsion‐lateral expansion/contraction metamaterials were devised based on spatial spiral rod systems^[^ ^47^ ^]^ (Figure 14i).

Moreover, the process by which an object exhibits different motion behaviors in two opposite directions is known as non‐reciprocal effect.^[^ ^412^ ^]^ The most renowned non‐reciprocal effect in modern natural science is the semiconductor diode effect, in which a spontaneous internal electric field is formed at the p‐n junction, causing the carrier transport process along a direction to be substantially different from that along the opposite direction. Another example of the non‐reciprocal effect is found in Tesla valves, which achieve unidirectional fluid flows. In condensed matter physics, non‐reciprocal effects are common in the propagation of electrons, phonons, magnetic excitons (spin waves), and light in crystals. For example, the Faraday rotation effect of crystals generally results in the unidirectional propagation of light. If the symmetry of a certain physical property in a crystal is broken (for example, magnetism corresponds to time‐reversal symmetry breaking, and ferroelectricity corresponds to space‐reversal symmetry breaking), the corresponding transport process exhibits non‐reciprocity, enabling the realization of switching or unidirectional controllable functions.^[^ ^412^ ^]^ Based on asymmetric inclined snapping beams and ligaments connected to rotational snapping elements, a special class of lattices were devised to exhibit static and non‐reciprocal mechanical motion effects.^[^ ^413^ ^]^ Besides, uniform composite materials with static non‐reciprocity were also developed based on the direction‐dependent buckling of embedded nanofillers^[^ ^414^ ^]^ (Figure 14j). Recently, based on symmetry breaking and contact nonlinearity, various non‐reciprocal mechanical responses were obtained, such as non‐reciprocal Poisson's ratio and non‐reciprocal RPE^[^ ^38^, ^415^ ^]^ (Figure 14k). Other anomalous deformation mechanisms, such as non‐Hermitian topology,^[^ ^416^, ^417^ ^]^ non‐orientable order,^[^ ^418^ ^]^ and static solitons,^[^ ^419^ ^]^ have also been extensively studied.

### 3.2 Physical Properties

The structure‐mechanism‐property relationships of lattice metamaterials can also be established in various physical fields, including acoustic, EM/optical, thermal, and other physical properties that do not fall in these commonly mentioned categories. More specifically, the acoustic properties of lattice metamaterials mainly refer to sound absorption and insulation properties. The EM properties of lattice metamaterials are primarily categorized as magnetic permeability, electric permittivity, refractive indices, and their optical properties are widely recognized and hence included as a subset of EM properties. Additionally, thermal conductivity and heat transfer efficiency are two major factors considered in the design of thermal lattices. In all of these physical domains, lattice metamaterials can be devised to achieve superior and/or unconventional properties by tailoring their internal microarchitectures.

#### 3.2.1 Acoustic Properties

Acoustic metamaterials typically refer to lattice metamaterials that can insulate or manipulate sound waves, and absorb sound.^[^ ^420^, ^421^ ^]^ These metamaterials are engineered to have unique microarchitectures and properties that allow them to interact with sound waves in a specific and controlled manner.^[^ ^422^ ^]^ Sound‐insulating metamaterials are commonly based on periodic structures that can attenuate sound wave propagation by creating Bragg's band gaps or locally resonating structures where sound waves cannot pass through during resonance.^[^ ^60^ ^]^ Lattice metamaterials that manipulate sound include structures that create negative effective mass density *ρ* and bulk modulus *K*.^[^ ^423^, ^424^ ^]^ These structures allow sound waves to be rerouted in a way such that they emerge on alternate sides of an object or focus on a specific area,^[^ ^420^ ^]^ enabling applications such as acoustic cloaking,^[^ ^425^ ^]^ ultrasound imaging,^[^ ^426^ ^]^ and acoustic tweezers.^[^ ^427^ ^]^ In turn, sound‐absorbing lattice metamaterials are structures that enable resonance modes for maximized air molecule vibrational energy losses during sound wave propagation.^[^ ^84^ ^]^

**Figure 15:**
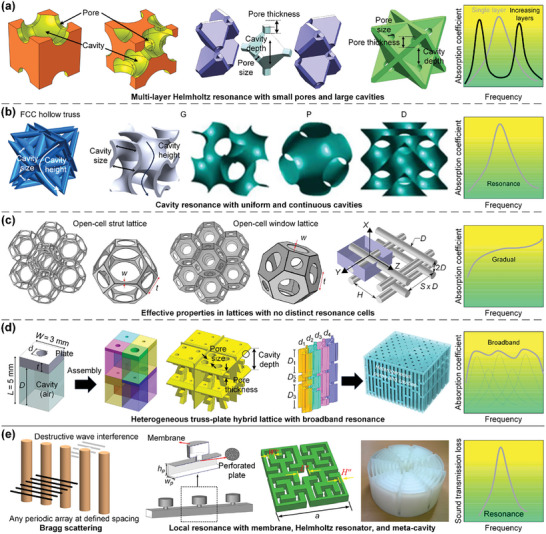
An overview of the structure‐mechanism‐property relationships of the sound absorption and insulation behaviors of lattice metamaterials. a) Lattice metamaterials functioning based on MLHR, associated with small pores and large cavities. Reproduced with permission.^[^ ^434^ ^]^ Copyright 2020, Elsevier. Reproduced with permission.^[^ ^17^ ^]^ Copyright 2021, Wiley. b) Lattice metamaterials functioning based on cavity resonance, associated with uniform and continuous cavities. Reproduced with permission.^[^ ^50^ ^]^ Copyright 2022, Wiley. Reproduced with permission.^[^ ^435^ ^]^ Copyright 2020, Taylor & Francis. c) Lattice metamaterials functioning based on effective property models, associated with uniform pores or cavities. Reproduced with permission.^[^ ^438^ ^]^ Copyright 2021, Elsevier. Reproduced with permission.^[^ ^439^ ^]^ Copyright 2019, AIP. d) The concept of heterogeneous lattices, based on an assembly of heterogeneously porous unit cells. Reproduced with permission.^[^ ^49^ ^]^ Copyright 2021, Wiley. Reproduced with permission.^[^ ^429^ ^]^ Copyright 2023, Wiley. For each case, their typical sound absorption coefficient curves and key acoustic geometries are denoted on the same figure. e) Bragg scattering via periodicity and local resonance for sound insulation. Reproduced with permission.^[^ ^442^ ^]^ Copyright 2021, Elsevier. Reproduced with permission.^[^ ^443^ ^]^ Copyright 2019, Elsevier. Reproduced with permission.^[^ ^444^ ^]^ Copyright 2015, Springer.

Thus far, studies on the acoustic properties of lattice metamaterials have mainly focused on sound absorption and insulation characteristics. In contrast to mechanical properties, the sound absorption properties of lattice metamaterials depend exclusively on their geometries, instead of their constitutive materials. Thus, the intricate structural porosity of lattice metamaterials facilitates a diverse range of acoustic behaviors. The processes of absorption, and consequently, dissipation of sound energy, primarily occur through frictional losses and thermal boundary losses as sound waves propagate across the surface of lattice materials. The extent of dissipation is intricately linked to the acoustically active geometric parameters of internal microarchitectures. Analytical models have exploited these geometric parameters to establish the relationship between the geometry of lattices and their sound absorption performances. Depending on the morphology of internal microarchitectures, these models incorporate mechanisms based on multi‐layer Helmholtz resonance (MLHR)^[^ ^17^, ^49^, ^428^, ^429^, ^430^, ^431^, ^432^, ^433^, ^434^ ^]^ (**Figure** **15**a), cavity resonance^[^ ^50^, ^435^, ^436^ ^]^ (Figure 15b), and effective properties^[^ ^437^, ^438^, ^439^ ^]^ (Figure 15c). The presence and effectiveness of these mechanisms depend on internal microarchitectures notably. The MLHR mechanism is based on the stacking of multiple Helmholtz resonators, which are resonators with a narrow neck and wide cavity that trap and amplify specific frequencies of sound. Consequently, the dissipation of sound energy is most pronounced at the neck region during resonance.^[^ ^17^, ^49^, ^428^, ^429^, ^430^, ^431^, ^432^ ^]^ The frequency and intensity of resonance are determined by the sizes of lattice pores and cavities, and the areal fraction occupied by pores. Thus, MLHR is the predominant sound absorption mechanism for lattice materials, offering high dissipation capabilities even at low sample thicknesses. In contrast, cavity resonance occurs within a cavity formed by the interference of standing waves, and is prominent in structures in which MLHR cannot occur, such as those comprising lattice materials without a narrow neck and wide cavity configuration.^[^ ^50^, ^435^ ^]^ The frequency and intensity of cavity resonance are dictated by cavity dimensions, such as its width and depth. Moreover, effective property models are typically empirical or phenomenological, and rely on specific parameters, such as pore sizes, surface porosity, and various derived metrics like tortuosity and characteristic lengths.^[^ ^437^ ^]^ Such models are typically applied to structures that lack prominent resonance features, or those with viscous boundary layer‐scale features.^[^ ^438^ ^]^ For example, foams characterized by a random arrangement of struts are often analyzed and modeled using effective property models,^[^ ^438^ ^]^ which can reliably predict sound absorption coefficients.

As previously outlined, our analysis has identified three primary sound absorption mechanisms from reports on various lattice materials. Considering the diversity of lattice configurations, we propose a comprehensive framework to elucidate the intricate relationships between lattice characteristics, underlying mechanisms, and the resultant sound absorption properties. In this context, we categorize acoustic lattices based on their acoustically active geometries into three distinct classes: 1) lattices with small pores and large cavities, 2) lattices with uniform and continuous cavities, and 3) lattices with uniform pores and cavities. Acoustic lattices with numerous small pores and large cavities operate based on MLHR. These lattices include high relative density truss lattices,^[^ ^17^, ^30^, ^50^ ^]^ plate lattices with embedded pores,^[^ ^17^, ^432^ ^]^ and intricately engineered lattices.^[^ ^49^, ^429^ ^]^ Despite their visual dissimilarity, all of these lattices possess narrow pores and wide cavities. In high relative density truss lattices, pores manifest at strut intersections, with the remainder constituting expansive cavities. Besides, plate lattices with embedded pores and intricately designed microarchitectures can be easily identified. In several instances, these lattices have been explicitly designed to promote MLHR. Examples of a plate and intricately designed lattices are shown in Figure 15a. Functioning based on MLHR, the resulting absorption curves usually exhibit resonance peaks with high amplitudes and narrow bandwidths. It is imperative to maintain a low surface porosity (<10%), as higher porosities typically lead to deviations from Helmholtz resonance. In comparison, acoustic lattices with uniform and continuous cavities generally exhibit cavity resonance. This category encompasses various sheet TPMS lattices derived from the inversion of truss lattices.^[^ ^50^, ^435^ ^]^ As shown in Figure 15b, these lattices are generally characterized by continuous and highly uniform air cavities. For example, sheet G‐type lattices with constant zero mean curvature consist of continuous sheets separated by a consistent distance. Consequently, G‐type shell lattices do not possess the characteristic geometry associated with Helmholtz resonators; instead, they exhibit cavity resonance and feature absorption peaks akin to Helmholtz resonators. Furthermore, acoustic lattices with uniform pores and cavities include low‐density trusses,^[^ ^437^ ^]^ woodpile trusses,^[^ ^439^ ^]^ etc. As shown in Figure 15c, these lattices exhibit more uniform pores and cavities than those shown in Figure 15a,b. The sound‐absorption properties of these lattices are predominantly governed by overall airflow resistance, and they can be accurately predicted using effective property models. Consequently, their properties resemble those of foams, and thus they exhibit smooth and gradual absorption curves. Additionally, certain lattices exhibit heterogeneous features, both in terms of structural design and acoustic mechanisms. One frequently studied form involves lattices composed of partitioned heterogeneous cells, such as Helmholtz resonance cells arranged in parallel.^[^ ^49^, ^428^, ^429^ ^]^ In this configuration, multiple resonance modes operate concurrently, resulting in a superimposed absorption curve derived from constituent elements (Figure 15d). This form effectively achieves broadband absorption within Helmholtz resonators. Similar broadband behavior is observed in FGLs with unit cells that vary in the direction of sound incidence.^[^ ^439^ ^]^ To date, acoustic lattices characterized by heterogeneous mechanisms include those based on hollow trusses and trusses featuring sonic black hole plate features.^[^ ^50^, ^440^ ^]^ Such configurations exploit the synergistic properties generated by the superposition of mechanisms of constituent phases, resulting in enhanced absorption bandwidths.

For sound insulation, there are two primary sound attenuation mechanisms in lattice metamaterials. The first mechanism is Bragg scattering via periodicity, and the second mechanism is local resonance.^[^ ^441^ ^]^ Typically, Bragg scattering occurs when the wavelength of sound waves matches the spacing of scatterers, creating destructive interference that attenuates specific frequencies, which results in band gaps where sound waves cannot propagate.^[^ ^60^ ^]^ Such metamaterials are commonly termed as sonic crystals, which are essentially periodic arrangements of any structures or features (Figure 15e). Locally resonating structures insulate sound using resonators that vibrate at specific frequencies. These resonators trap sound energy and convert it into other forms, such as heat, thereby dissipating it and preventing transmission. The representative examples include Helmholtz,^[^ ^442^ ^]^ cavity,^[^ ^443^ ^]^ and Mie^[^ ^444^ ^]^ resonators (Figure 15e). Additionally, sonic crystals are often embedded with features enabling local resonance. These combined effects make sonic crystals effective in isolating sound of certain frequencies, thus providing efficient sound insulation. The resonance effects are also highly tuneable, allowing for precise control over which frequencies are attenuated, making these structures particularly useful for targeted sound insulation applications. Till date, the potential of lattice metamaterials as sonic crystals for sound insulation have been affirmed. The potential of hybrid tubular and plate lattices as sonic crystals with embedded Helmholtz resonators has been demonstrated^[^ ^60^ ^]^ Experimental investigations on a lattice design comprising three layers with a 20 mm unit cell reveal a maximal sound attenuation of 32 dB at 1810 Hz. Besides, another strong attenuation band emerged past 5000 Hz, in which analytical calculations and numerical simulations identified local Helmholtz resonance and Bragg scattering as corresponding attenuation mechanisms, respectively.

#### 3.2.2 Electromagnetic/Optical Properties

The EM properties of lattice metamaterials are typically derived from those of their constitutive materials but can be further enhanced by the design of internal microarchitectures. The optical properties of lattice metamaterials are also included in this section, since light is also an EM wave. Generally, the intrinsic EM properties of a material are characterized by two physical parameters, namely electric permittivity *ε* and magnetic permeability *µ*. The appropriate design of internal microarchitectures can enable lattice metamaterials to achieve numerous unconventional EM properties, such as negative *ε*, negative *µ*, and negative refractive indices (left‐handed property).^[^ ^51^, ^52^, ^65^, ^445^, ^446^ ^]^ These extraordinary properties can enable the free manipulation of EM fields, leading to the realization of novel physical phenomena that do not exist in nature, including subwavelength imaging, perfect cloaking or absorption, and hyperlenses.^[^ ^81^, ^447^ ^]^ However, the microarchitecture design of EM metamaterials needs to be confined to a sub‐wavelength scale to realize these properties, thus very challenging, or at least very costly for traditional manufacturing methods to achieve, especially for microwave or terahertz (THz) frequency bands. Although traditional manufacturing techniques such as lithography can be used to fabricate EM metamaterials, the small fabrication scales limit their widespread applications. Recent advancements in AM techniques have significantly enhanced the flexibility and versatility in the design and fabrication of EM metamaterials, particularly at sub‐wavelength scales. Consequently, additively manufactured lattice metamaterials show great promise for achieving diverse but controllable unique EM properties.

The dielectric constant is one of the fundamental physical parameters of a material, determining its response to EM waves or electric fields, which generally takes a positive value. However, through appropriate design of internal microarchitectures, lattice metamaterials can be engineered to achieve negative dielectric constants, which result in unusual and remarkable EM characteristics, such as the reverse Doppler effect and anomalous Cherenkov radiation.^[^ ^448^, ^449^ ^]^ These properties can be utilized for perfect EM wave absorption and ultra‐low loss wireless power transmission.^[^ ^51^, ^450^ ^]^ The AM procedures of lattice metamaterials provide a larger design freedom for the dielectric control of EM metamaterials, including the structural design and component adjustment. More specifically, lattice metamaterials with specific dielectric constant values can be devised by incorporating dielectric constitutive materials. Besides, the geometric configurations affect the electric field distribution within the lattices, thus allowing for the precise tuning of their dielectric constants. For instance, tailorable high and low dielectric constant filaments can be achieved by utilizing dielectric‐tuneable polymer‐based micro‐ceramic composites as constitutive materials and adopting the dual‐extrusion printing.^[^ ^451^ ^]^ This metamaterial can manipulate microwave operating frequency and Mie resonance amplitude, thereby offering unprecedented EM characteristics, such as near‐zero or less than unity values of effective permittivity. Furthermore, the selection of appropriate constitutive materials and the design of internal microarchitectures can effectively regulate the conductivity of lattice metamaterials. By controlling the conductivity, the dielectric properties of the lattices can be affected as well. For example, conductive materials such as metals and doped polymers can be used to enhance the conductivity of lattice metamaterials.^[^ ^452^ ^]^ Besides, the geometric configurations also affect the overall conductivity of lattice metamaterials significantly, in which the key factors include the thickness of struts, connectivity, and porosity. The control of constitutive materials and internal microarchitectures is essential for devising lattice metamaterials with specific dielectric responses for applications in capacitors, waveguides, and resonators. Additionally, magnetic permeability is another critical parameter that can be controlled in lattice metamaterials through AM processes.^[^ ^453^ ^]^ The incorporation of magnetic constitutive materials and the design of specific geometric patterns that enable magnetic resonance can significantly enhance the permeability of the lattices. For instance, split‐ring resonators and other magnetic inclusions can be embedded within the lattices to achieve target magnetic responses.^[^ ^454^, ^455^, ^456^ ^]^ Besides, AM techniques can facilitate the precise placement of these features, thereby enabling the design of lattice metamaterials with customized magnetic permeability. For example, FDM‐printed multilayer metamaterials with magnetic carbonyl iron and polyether‐ether‐ketone (PEEK) as material filaments exhibit tailored permeability via adjusting the geometric parameters.^[^ ^457^ ^]^

**Figure 16:**
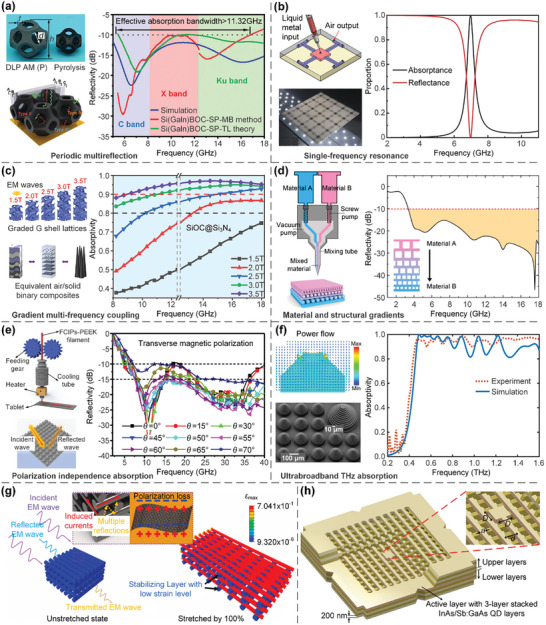
An overview of the structure‐mechanism‐property relationships of EM and optical lattice metamaterials. a) EM wave absorption properties of Schwarz P‐type TPMS shell lattices composed of SiBOC ceramics with uniformly distributed gallium indium alloy nanoparticles (Si(Galn)BOC) for an ultrawide effective absorption band. Reproduced with permission.^[^ ^470^ ^]^ Copyright 2023, Wiley. b) Single‐frequency resonance for lattice metamaterials with highly conductive microchannels. Reproduced with permission.^[^ ^463^ ^]^ Copyright 2020, Springer. c) Gradient G‐type TPMS shell lattices composed of polymer‐derived SiOC ceramics encapsulated by Si_3_N_4_ (SiOC@Si_3_N_4_) for multi‐frequency coupling. Reproduced with permission.^[^ ^464^ ^]^ Copyright 2023, Wiley. d) Material and structural graded lattices for tailored broadband EM responses. Reproduced with permission.^[^ ^467^ ^]^ Copyright 2022, Elsevier. e) Rotationally symmetric lattices composed of flaky carbonyl iron particles (FCIPs) and PEEK matrix for polarization independence wide‐angle absorption. Reproduced with permission.^[^ ^468^ ^]^ Copyright 2021, Elsevier. f) Ultrabroadband THz absorption of EM lattices by stacking multilayer concentric resonators on different‐level top surfaces of a monolithic 3D pagodalike substrate. Reproduced with permission.^[^ ^469^ ^]^ Copyright 2021, APS. g) Schematic illustration of the EMI shielding mechanism under large tensile strains in graphene/polydimethylsiloxane composite lattices. Reproduced with permission.^[^ ^471^ ^]^ Copyright 2021, Elsevier. h) Illustration of a high‐Q 3D photonic crystal lattice with a point‐defect nanocavity coupled to quantum dots. Reproduced with permission.^[^ ^476^ ^]^ Copyright 2009, AIP.

AM techniques possess superior capabilities in controlling the magnetic permeability and electric permittivity of lattice metamaterials to achieve customized EM performances.^[^ ^458^ ^]^ This precision allows lattice metamaterials to effectively couple EM fields, thereby facilitating the absorption and shielding of EM waves. These properties are highly beneficial for applications in modern science and engineering.^[^ ^459^ ^]^ The absorption mechanism of EM energy within lattice metamaterials is the conversion of incident EM energy into other forms of energy, such as heat energy via resonance and coupling, or interference cancellation.^[^ ^445^ ^]^ Depending on the configurations of microarchitectures and the properties of constitutive materials, the design principles and absorption mechanisms of EM metamaterials can be summarized as periodic multireflection,^[^ ^460^, ^461^, ^462^ ^]^ single‐frequency resonance,^[^ ^463^ ^]^ gradient multi‐frequency coupling,^[^ ^464^, ^465^ ^]^ material and structural gradients,^[^ ^466^, ^467^ ^]^ polarization independence absorption,^[^ ^468^ ^]^ and ultrabroadband THz absorption.^[^ ^469^ ^]^ The complex and synergistic interactions among constitutive materials, internal microarchitectures, and homogenized properties play a key role in additively manufactured EM lattice metamaterials. The core of this material‐structure‐property relationship is the design of microarchitectures, defined by the periodicity, geometry, and arrangement of lattice unit cells, which contribute to the fully controllable EM responses. The constitutive materials, whether metallic, dielectric, or a combination thereof, inherently possess certain EM properties, such as electric permittivity and magnetic permeability, and result in dielectric or magnetic loss capabilities for EM energies. However, when architected into specific geometric configurations, these lattices transcend the conventional limitations of constitutive materials and exhibit customizable unconventional capabilities when interacting with EM waves. Herein, according to the principles and mechanisms discussed above, we summarize the design principles of EM lattices for each type of absorption mechanism. First, in case of periodic multireflection designs, the inherent properties of constitutive materials, combined with the specific arrangement of internal microarchitectures, result in enhanced resonance and interference of EM waves. For example, the Schwarz P‐type TPMS shell lattices‐based metastructure enables tailorable control of dielectric constants via an intricate EM wave interference cancellation mechanism (**Figure** **16**a), thereby enlarging the absorption band effectively.^[^ ^470^ ^]^ Second, the single‐frequency resonance designs focus on maximizing the absorption efficiency at a specific frequency of EM waves, which is typically achieved by devising highly conductive patterns on high‐impedance surfaces to generate strong and near‐perfect resonances for absorption on the surfaces of specific frequencies (Figure 16b). These designs possess potential applications in tuneable microwave filters.^[^ ^463^ ^]^ Additionally, gradient multi‐frequency coupling takes the concept of stepwise impedance matching to capture EM waves as much as possible and adopts the gradient increasing of dissipation to achieve EM absorption black holes^[^ ^464^ ^]^ (Figure 16c). The effectiveness of this approach is predominantly dependent on the precise design of internal microarchitectures to ensure optimal impedance matching and coupling across a broadband frequency spectrum. Besides, material and structural gradient designs showcase the potential of combining different materials and structures to achieve a tailored broadband EM response^[^ ^467^ ^]^ (Figure 16d), in which each layer is devised for contributing to different EM losses, thereby culminating in a broad‐spectrum absorption performance. The success of this design hinges on the appropriate selection of constitutive materials for each layer and their spatial arrangements to ensure that the homogenized properties are aligned with target EM responses. Moreover, polarization‐independent absorption designs are pivotal in applications requiring omnidirectional absorption, in which the design of lattice microarchitectures to eliminate anisotropy is necessary. These lattices rely on rotational symmetry to absorb EM waves regardless of their polarization, such as the wide‐angle absorbing metastructures^[^ ^468^ ^]^ (Figure 16e). Furthermore, microscale designs for ultrabroadband THz absorption underscore the precision required in lattices to interact with specific wavelengths. By utilizing AM techniques, EM metamaterials can be fabricated and manipulated at microscale levels, showcasing the unique interaction and absorption between architected materials and THz waves^[^ ^469^ ^]^ (Figure 16f). Lastly, composite lattices can be devised to achieve tuneable mechanical and EM interference (EMI) shielding properties by adopting highly ordered and precisely controlled microarchitectures^[^ ^471^ ^]^ (Figure 16g). More specifically, precisely tailored graphene/polydimethylsiloxane composite lattices can achieve an exceptional stretchability of 130%, a tunable EMI shielding effectiveness of up to 45 dB, and superior durability, demonstrating a retention of EMI shielding effectiveness of over 90% even after 200 cycles of repetitive stretching and releasing under tensile strains as high as 100%.^[^ ^471^ ^]^ In summary, these material‐structure‐property relationships validate the importance of AM techniques in the design of EM lattices. Typically, AM techniques allow for the precise control and combination of constitutive materials and internal microarchitectures, thereby enabling to achieve tailored EM performances for a broad range of EM applications.^[^ ^469^ ^]^

As a subset of EM waves, light waves also exhibit unique EM phenomena induced by the design of internal microarchitectures in lattice metamaterials, such as negative refractive indices, negative electric permittivity, and negative magnetic permeability. These properties offer substantial potential for multifunctional applications in light field control, laser beam modulation, optical cloak, and light sensors of optical metamaterials.^[^ ^472^ ^]^ Typically, AM techniques enable the design and control of resonant units in lattice metamaterials, thus allowing the manipulation of light in ways that natural materials cannot achieve. This includes the realization of optical negative refraction,^[^ ^473^ ^]^ hyperbolic dispersion,^[^ ^474^ ^]^ super‐resolution imaging,^[^ ^475^ ^]^ and high‐quality (*Q*) factors^[^ ^476^ ^]^ (Figure 16h). For example, a 3D optical metamaterial with cascaded ‘fishnet’ structures was devised for the first time to achieve negative refractive indices in a wide spectral range.^[^ ^477^ ^]^ This metamaterial realizes negative refraction at optical frequencies through the negative phase evolution of waves propagating within its internal microarchitectures. This breakthrough provides a possibility for exploring various optical phenomena related to zero and negative refractive indices, and their applications in photonics and miniaturized imaging. Additionally, to reduce surface scatterings, an impedance‐matched hyperlens was devised based on the transformation optics principle.^[^ ^478^ ^]^ Besides, microwave experiments^[^ ^479^ ^]^ have demonstrated the feasibility of a transmuted Eaton lens,^[^ ^480^ ^]^ thereby enabling the realization of the cat's eye effect in all directions. A rectangular region in the virtual space can likewise be mapped onto a bending physical space via transformation optics, which enables to guide EM waves around desired bending regions.^[^ ^481^ ^]^ In daily life, transformation optics can serve as a tool for light‐bending parts, such as a perfect lens with *ε* = *µ* = −1 to achieve perfect imaging properties.^[^ ^482^ ^]^ Moreover, optical metamaterials can process wavefronts with the spatial resolution in sub‐wavelength, enabling efficient large‐angle light bending and focusing, complete the control of light phase and polarization, and realize the adjustment of orbital angular momentum for specific functionalities.^[^ ^483^ ^]^ For instance, 3D‐printed spiral phase plates can be utilized for the multiplexing and demultiplexing of different orbital angular momentum modes. This technique can fully exploit the THz band, expanding the free‐space optical communication range.^[^ ^484^ ^]^ Similarly, by implementing isotropic metamaterials in an alternating layered system, a wave combiner was fabricated to split, shift, and combine waves.^[^ ^485^ ^]^ Beyond traditional light field manipulation, AM procedures of optical metamaterials have also been utilized for the creation and manipulation of quantum light. For instance, metasurfaces composed of nano‐resonators can achieve quantum multi‐photon interference and state reconstruction,^[^ ^486^ ^]^ and dielectric metasurfaces made of anisotropic nano‐antennas can realize the quantum entanglement of photonic spin and orbital angular momentum^[^ ^487^ ^]^ This demonstrates that the control of light by AM of optical metamaterials can transcend classical optics and venture into the realm of quantum optics, thus opening new opportunities for the development of advanced optical devices. In addition, many other optical properties of lattice metamaterials based on transformation optics have also been reported, such as hidden gateways,^[^ ^488^, ^489^ ^]^ optical black holes,^[^ ^490^, ^491^, ^492^ ^]^ and EM wormholes.^[^ ^493^ ^]^

#### 3.2.3 Thermal Properties

Lattice metamaterials enabled by AM techniques generally possess high surface‐to‐volume ratios and porosities, which can significantly enhance their thermal properties. Unlike stochastic foams, the periodic microarchitectures of lattice metamaterials allow for substantial structural controllability and design freedom to meet various requirements of thermal properties. Among all considerations in thermal lattice metamaterials, thermal conductivity, and heat transfer efficiency are two key factors.^[^ ^53^, ^54^, ^55^, ^89^ ^]^ Typically, thermal conductivity is determined by both microarchitecture configurations and material properties, including those of constitutive materials and fluid media,^[^ ^53^, ^54^, ^55^ ^]^ while heat transfer efficiency is predominantly determined by microarchitecture configurations.^[^ ^55^, ^89^, ^494^ ^]^ The active cooling performance benefits afforded by optimizing the thermal properties of materials are generally within a factor of half of those afforded by optimizing the configurations of microarchitectures.^[^ ^495^ ^]^ Therefore, we predominantly focus on the effects of microarchitecture design on the thermal properties of lattice metamaterials in this section.

**Figure 17:**
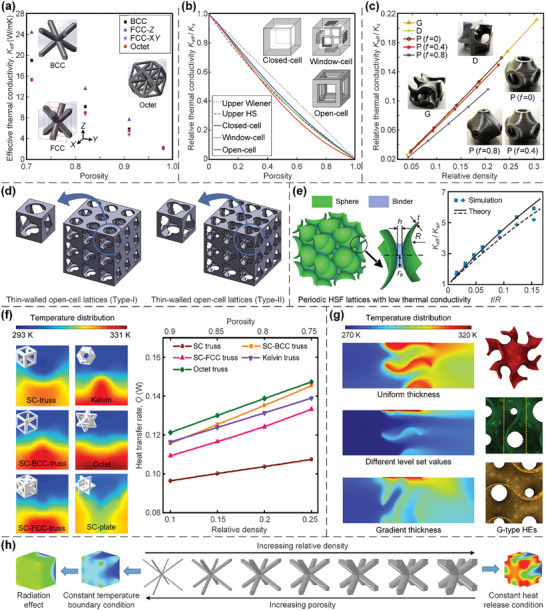
An overview of the structure‐mechanism‐property relationships of thermal lattice metamaterials. a) The design of truss lattices to achieve tailorable effective thermal conductivities. Reproduced with permission.^[^ ^55^ ^]^ Copyright 2024, Elsevier. b) The thermal conductivities of closed‐cell (SC plate), window‐cell, and open‐cell (SC truss) lattices. Reproduced with permission.^[^ ^499^ ^]^ Copyright 2019, Elsevier. c) The thermal conductivities of different types of TPMS shell lattices. Reproduced with permission.^[^ ^53^ ^]^ Copyright 2019, Elsevier. d) The design of novel thin‐walled open‐cell lattice metamaterials to achieve tailorable thermal conductivities. Reproduced with permission.^[^ ^53^ ^]^ Copyright 2019, Elsevier. e) The design of periodic HSF lattices to obtain low thermal conductivities (comparable to that of air) for thermal insulation. Reproduced with permission.^[^ ^54^ ^]^ Copyright 2020, AIP. f) The heat transfer efficiency and pressure drop of representative truss and plate lattices, with truss lattices demonstrating lower convective heat transfer rate and lower pressure drop, while plate lattices exhibiting a contrary trend. Reproduced with permission.^[^ ^505^ ^]^ Copyright 2021, Elsevier. g) The design of functionally graded G‐type HEs with different level set values and varying wall thicknesses to maximize heat transfer efficiency while minimizing pressure drop. Reproduced with permission.^[^ ^507^ ^]^ Copyright 2023, Elsevier. h) The conduction, convection, and radiation heat transfer in BCC lattice metamaterials with a wide range of porosities. Reproduced with permission.^[^ ^496^ ^]^ Copyright 2022, Elsevier.

Heat transfer generally occurs in three forms, namely conduction, convection, and radiation, which correspond to various service conditions and applications. Lattice metamaterials play a vital role in enhancing heat transfer via all the three forms.^[^ ^496^ ^]^ Typically, heat conduction involves solid–solid, solid–liquid, and solid–gas conduction, with solid‐solid conduction exhibiting the highest heat transfer efficiency. Unlike in solid–liquid conduction, the heat in solid‐solid conduction is mainly transferred along the solid parts of structures. Hence, the thermal conductivity of lattice metamaterials is strongly related to their relative densities and wall thicknesses.^[^ ^496^ ^]^ The structural design variables of truss lattices, including topology, cross‐sectional area, specific surface area, and porosity, have been thoroughly studied in relation to their effective thermal conductivities to achieve tailorable heat conduction performances.^[^ ^55^, ^496^, ^497^, ^498^ ^]^ For example, additively manufactured SC truss, face diagonal cubic, tetrakaidecahedron, and octet truss lattices exhibit close effective thermal conductivities at high porosities (*p *≥ 0.9), while the effect of microarchitecture configurations on effective thermal conductivities becomes more significant at relatively lower porosities (*p *< 0.9).^[^ ^498^ ^]^ Furthermore, given the same porosity, AlSi10Mg‐based FCC lattices exhibit the highest thermal conductivity along *z*‐axis, followed by BCC, *x*‐ or *y*‐axis of FCC lattices, and octet lattices (**Figure** **17**a). The thermal conductivity exhibits a general increasing trend with decreasing porosities or decreasing specific surface areas, and more strikingly, increasing the minimal cross‐sectional area in the primary conduction direction can significantly increase the thermal conductivity by nearly 51%.^[^ ^55^ ^]^ For closed‐cell (SC plate), window‐cell, and open‐cell (SC truss) lattices, closed‐cell SC plate lattices are shown to possess the highest relative conductivities, open‐cell SC truss lattices exhibit the lowest ones, while window‐cell lattices possess intermediate values given the same porosity^[^ ^499^ ^]^ (Figure 17b). In comparison, TPMS shell lattices exhibit a narrower range of thermal conductivity than that of truss lattices,^[^ ^53^, ^494^, ^500^, ^501^ ^]^ among which the effective thermal conductivities of FDM printed polylactic acid (PLA)‐based G‐, D‐, and P‐type shell lattices (with a zero level‐cutting parameter *f* = 0) are close to each other^[^ ^53^ ^]^ (Figure 17c). The three types of lattices exhibit a decrease trend in thermal conductivity with an increase in unit cell size,^[^ ^494^ ^]^ and the thermal conductivity of P‐type lattices decreases with an increase in the level‐cutting parameter (from *f* = 0 to 0.4 then 0.8)^[^ ^53^ ^]^ (Figure 17c). Typically, lattice metamaterials with larger unit cells possess larger wall thicknesses and tend to result in higher thermal conductivities.^[^ ^502^ ^]^ In addition, the induced contact that causes changes in the conductive path area can be used to achieve variable thermal conductivity. The contact can be externally generated by an applied mechanical loading, or internally generated by the thermal strain induced by shape memory alloys, bimetal springs, and CTE mismatch. By dynamically altering the bulk thermal conductivity for regulating the instantaneous heat flux through their internal microarchitectures, lattice metamaterials are capable of controlling the temperature of an interface actively.^[^ ^503^ ^]^ Additionally, other forms of lattice metamaterials have also been devised to obtain programmable thermal properties, including the design of novel thin‐walled open‐cell lattices with different tailored hollow patterns along different directions to achieve a large tailorable range of thermal conductivities^[^ ^53^ ^]^ (Figure 17d), the design of periodic hollow sphere foam (HSF) lattices with low thermal conductivity (comparable to that of air) for thermal insulation^[^ ^54^, ^504^ ^]^ (Figure 17e), and the design of architected FGLs to regulate the temperature field by tuning the thermal flow inside the porous media.^[^ ^53^ ^]^

Besides, variations in the microarchitecture configurations of lattice metamaterials mainly affect convective heat transfer in medium (e.g., air, water, or oil), and the pressure drop and Reynolds number significantly affect the heat transfer efficiency.^[^ ^505^, ^506^ ^]^ Typically, a larger pressure drop is a necessary trade‐off for achieving higher heat transfer efficiency.^[^ ^507^, ^508^ ^]^ A decrease in porosity tends to enhance heat transfer rate and increase pressure drop, since lower porosity generally results in a larger interactive surface area at a constant unit‐cell volume.^[^ ^505^ ^]^ Truss lattices, despite exhibiting the lowest pressure drop, generally possess the lowest convective heat transfer rate among truss, plate, and shell lattices^[^ ^496^, ^505^ ^]^ (Figure 17f). Among truss lattices, octet lattices exhibit the highest heat transfer rate, SC lattices exhibit the lowest rate, while SC‐BCC, SC‐FCC, and Kelvin lattices exhibit intermediate rates. All of these lattices show a gradual increase trend in heat transfer rate and pressure drop with increasing relative densities (Figure 17f), and a sudden increase trend is found in the pressure drop of SC‐FCC lattices at moderate relative densities.^[^ ^505^ ^]^ Typically, despite possessing superior convective heat transfer rates, plate lattices demonstrate a remarkably higher pressure drop than truss lattices, which limits their application for use as heat exchangers (HEs) owing to the considerably higher pumping power expense (Figure 17f).^[^ ^505^ ^]^ Moreover, some studies conduct numerical simulations of pure conduction and natural convection under isothermal circumstances and demonstrate that TPMS shell lattices possess superior overall thermal performances.^[^ ^502^, ^507^, ^508^, ^509^ ^]^ More specifically, a thorough study is conducted on the heat transfer performances of seven classical types of TPMS shell lattices, among which D‐ and P‐type lattices exhibit the best and worst overall performances, respectively.^[^ ^508^ ^]^ Strikingly, D‐type HEs only need a volume of 3–10 times lower than that of traditional tubular HEs to obtain the same heat transfer performance give the same pressure drop, showcasing great potential for industrial applications.^[^ ^508^ ^]^ Furthermore, functionally graded G‐type HEs with different level set values and varying wall thicknesses are devised, whose heat transfer efficiency is enhanced by 26%‐60% as compared to that of uniform G‐type HEs, and the pressure drop is decreased by 10–18% simultaneously^[^ ^507^ ^]^ (Figure 17g). Moreover, radiative heat transfer also affects the thermal conductivity of lattice metamaterials, especially in case of high porosities.^[^ ^496^ ^]^ Typically, radiative thermal conductivity increases with an increase in porosity, and this increase is linearly related to the unit cell size. By increasing the temperature difference and solid radiative emission coefficient, the increase can be slightly enhanced. Generally, heat conduction and heat radiation significantly affect the effective thermal conductivity of lattice metamaterials with lower and higher porosities, respectively.^[^ ^496^ ^]^ When the flow Reynolds number and the thermal conductivity of constitutive materials are low, or when the porosity is high, radiative heat transfer in BCC truss lattices generally cannot be ignored under the condition of constant heat release rate^[^ ^496^ ^]^ (Figure 17h). Overall, the aforementioned findings provide practical insights for devising lattice metamaterials with superior and tuneable thermal properties.

### 3.3 Other Properties

As mentioned above, appropriate microarchitecture designs, when combined with constitutive materials with specific mechanical/physical properties, can enable lattice metamaterials to achieve further enhanced mechanical,^[^ ^10^, ^11^, ^12^ ^]^ acoustic,^[^ ^49^, ^60^, ^420^ ^]^ EM/optical,^[^ ^52^, ^65^, ^445^ ^]^ and thermal^[^ ^53^, ^55^, ^89^ ^]^ properties far beyond those of bulk materials, even reaching their respective theoretical limits.

#### 3.3.1 Other Mechanical Properties

**Figure 18:**
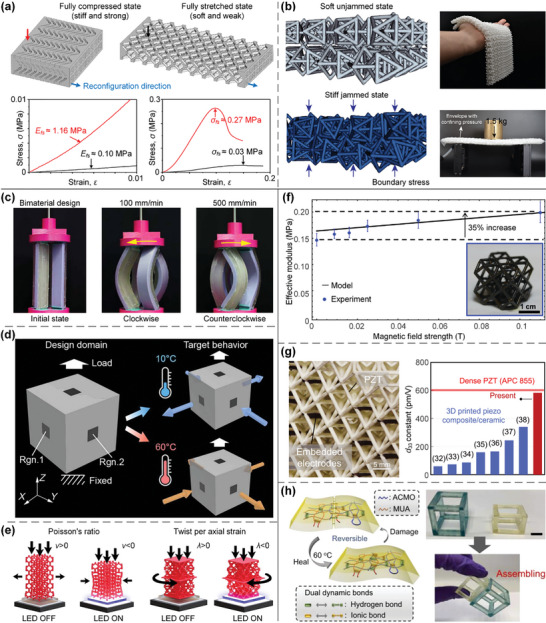
An overview of the other mechanical/physical properties of lattice metamaterials. a) Multi‐stable lattice metamaterials with tuneable stiffness and strength. Reproduced with permission.^[^ ^333^ ^]^ Copyright 2018, Elsevier. b) Pressure‐induced jamming phase transitions of interpenetrating lattices with tuneable stiffness and strength. Reproduced with permission.^[^ ^510^ ^]^ Copyright 2021, Springer. c) Strain rate‐dependent compression‐torsion deformation modes of bi‐material lattices. Reproduced with permission.^[^ ^512^ ^]^ Copyright 2020, AAAS. d) Tuneable mechanical properties of lattice metamaterials via an applied thermal field. Reproduced with permission.^[^ ^24^ ^]^ Copyright 2023, AAAS. e) Tuneable mechanical properties of lattice metamaterials induced by an external light from a blue LED. Reproduced with permission.^[^ ^528^ ^]^ Copyright 2022, Elsevier. f) Tuneable mechanical properties of lattice metamaterials via an applied magnetic field. Reproduced with permission.^[^ ^58^ ^]^ Copyright 2018, AAAS. g) Additively manufactured piezoelectric ceramics with embedded electrodes, including an image of 3D‐printed piezoelectric lattice block with embedded 3D electrodes and the piezoelectric charge coefficients of additively manufactured piezoelectric lattice metamaterials. Reproduced with permission.^[^ ^557^ ^]^ Copyright 2022, AAAS. h) DLP 3D printing of polymer composites with self‐healing properties, including the schematic representation of self‐healing properties functionalized by ionic bonds (yellow cylinders) and reversible hydrogen bonds (green cylinders), and the assembling of multi‐material structures with soft and rigid composites. Reproduced with permission.^[^ ^570^ ^]^ Copyright 2023, Elsevier.

In addition to the above‐mentioned mechanical properties of lattice metamaterials in Section 3.1, the other mechanical properties include the adjustment of the stiffness and strength of lattice metamaterials based on bistable/multi‐stable shape reconfigurations.^[^ ^333^ ^]^ and pressure‐induced jamming phase transitions^[^ ^510^ ^]^ Moreover, except for the aforementioned static/quasi‐static mechanical properties, when considering dynamic situations, some mechanical properties of lattice metamaterials may also change with the strain rate,^[^ ^511^ ^]^ such as the hardening of stiffness,^[^ ^244^ ^]^ weakening of Poisson's effect,^[^ ^244^ ^]^ strain rate‐dependent deformation modes,^[^ ^512^ ^]^ viscoelasticity‐induced pseudo bistability/multi‐stability,^[^ ^513^ ^]^ and wave manipulation of phononic crystals.^[^ ^514^, ^515^, ^516^, ^517^, ^518^, ^519^, ^520^ ^]^ More specifically, the passive variable stiffness, strength, and energy absorption performance of lattice metamaterials in the transverse direction can be realized through axial shape reconfigurations^[^ ^333^ ^]^ (**Figure** **18**a). The stiffness and strength in the fully enclosed state are approximately one order of magnitude larger than those in the fully open state. Moreover, tuneable stiffness and strength can be achieved by pneumatically controlling the jamming phase transitions of interpenetrating lattices^[^ ^510^ ^]^ (Figure 18b). In addition, the interaction between mechanical instability and viscoelasticity can result in strain rate‐dependent compression‐torsion deformation modes within bi‐material lattices^[^ ^512^ ^]^ (Figure 18c).

Moreover, different mechanical properties of lattice metamaterials may exhibit different coupling mechanisms. For example, most lattices exhibit a consistent trend between stiffness and strength, in which stretching‐dominated lattices generally possess higher stiffness and strength than bending‐dominated lattices with equal relative densities.^[^ ^11^, ^14^, ^151^ ^]^ Typically, plate/truss lattices possess the highest/lowest stiffness and strength given the same relative density; in comparison, shell lattices outperform truss lattices while falling behind plate lattices with equal relative densities in stiffness and strength.^[^ ^8^, ^11^, ^14^, ^521^ ^]^ However, in some cases, a decrease in elastic modulus can effectively enhance the strength of lattice metamaterials, resulting in a trade‐off between their stiffness and strength. For instance, the introduction of slightly corrugated geometries into SC plate lattices can efficiently enhance their stability and strength via buckling prevention at ultra‐low relative densities, albeit at the cost of lower stiffness.^[^ ^151^ ^]^ Additionally, the introduction of hierarchical microarchitectures can also effectively enhance the stability and strength of lattice metamaterials via buckling prevention at ultra‐low relative densities, while their stiffness is decreased significantly.^[^ ^167^ ^]^ In addition, for the coupling mechanisms between strength and energy absorption, strength and toughness, strength and recoverability, strength and ductility, the majority of lattice metamaterials exhibit a trade‐off correlation. More specifically, stretching‐dominated lattices typically undergo a layer‐by‐layer plastic buckling failure mode under large compressive strains, which results in consecutive sudden drops in the stress‐strain curves and reduces the energy absorption capabilities significantly.^[^ ^31^, ^522^ ^]^ In contrast, the stress‐strain curves of bending‐dominated lattices generally exhibit a long and flat plateau region under large compressive strains, leading to superior energy absorption capabilities.^[^ ^31^, ^522^ ^]^ However, the appropriate design of internal microarchitectures can sometimes enable simultaneous enhancement of the strength, energy absorption capability, and toughness of the lattices. For instance, plate and shell lattices generally outperform truss lattices with equal relative densities in strength and energy absorption capability significantly.^[^ ^523^, ^524^ ^]^ Besides, the introduction of functionally graded microarchitectures can also efficiently enhance both the strength and energy absorption capability of the lattices simultaneously.^[^ ^185^, ^525^ ^]^ Furthermore, the strength and energy absorption capability of lattice metamaterials can also be effectively enhanced by incorporating multiple constitutive materials with different mechanical properties.^[^ ^526^ ^]^ For example, by incorporating hard PLA struts to maintain the stiffness/strength and soft TPU cores to enhance the energy absorption capability, architected multi‐material honeycomb lattices with superior strength and specific energy absorption can be achieved.^[^ ^526^ ^]^

#### 3.3.2 Coupling of Multi‐Physical Properties

Apart from the classic physical properties mentioned in Section 3.2, such as acoustic, EM/optical, and thermal properties, here we also consider the coupling of multi‐physical properties of lattice metamaterials that involve the coupling of properties from two different or even more mechanical/physical disciplines. The interaction mechanisms between different mechanical/physical properties remarkably affect the enhancement of multi‐physical properties of lattice metamaterials enabled by 3D/4D printing techniques, such as tuneable mechanical properties induced by an applied thermal,^[^ ^24^, ^527^ ^]^ optical,^[^ ^528^ ^]^ electric,^[^ ^529^ ^]^ or magnetic,^[^ ^58^ ^]^ field, thermomechanics^[^ ^323^ ^]^ acoustothermy,^[^ ^530^ ^]^ electrothermy,^[^ ^531^ ^]^ magnetothermy,^[^ ^532^ ^]^ piezoresistivity,^[^ ^533^ ^]^ piezocapacitivity,^[^ ^534^ ^]^ piezoelectricity,^[^ ^535^ ^]^ piezomagnetism,^[^ ^536^ ^]^ triboelectricity,^[^ ^537^ ^]^ thermoresistivity,^[^ ^538^ ^]^ thermoelectricity,^[^ ^539^ ^]^ thermomagnetism,^[^ ^540^ ^]^ photoelectricity,^[^ ^541^ ^]^ and photomagnetism.^[^ ^542^ ^]^ More specifically, the elastic moduli,^[^ ^543^ ^]^ Poisson's ratios,^[^ ^544^ ^]^ band gaps,^[^ ^545^ ^]^ strengths,^[^ ^546^ ^]^ and deformation recovery^[^ ^546^ ^]^ of lattice metamaterials composed of active materials can be regulated by other physical fields, such as shape memory material‐based,^[^ ^547^ ^]^ liquid crystal elastomer‐based,^[^ ^548^ ^]^ and hydrophilic hydrogel‐based^[^ ^549^ ^]^ lattice metamaterials. Furthermore, lattice metamaterials made of two or more polymers with distinct glass transition temperatures have been leveraged to achieve programmable changes in mechanical properties and physical responses, such as elastic constants,^[^ ^550^ ^]^ deformation modes,^[^ ^527^ ^]^ pattern transformations,^[^ ^551^, ^552^ ^]^ band gaps,^[^ ^553^ ^]^ shape recovery,^[^ ^554^, ^555^ ^]^ and energy absorption performance^[^ ^556^ ^]^ via an applied thermal field^[^ ^24^ ^]^ (Figure 18d). Similarly, mechanical behaviors such as Poisson's ratio and torsional angle under axial loads can be controlled by an external light from a blue LED^[^ ^528^ ^]^ (Figure 18e). Besides, lattice microarchitectures in field‐responsive metamaterials typically enable reversible and dynamic tunability of mechanical properties, as demonstrated by magnetic field‐responsive lattices^[^ ^58^ ^]^ (Figure 18f). The stiffness change of these lattices is affected by both microarchitecture configurations and the direction of the magnetic field applied. In case the magnetic field is parallel applied to the direction of deformation, the effective stiffness of their constituent trusses is enhanced significantly. Additionally, the application of AM techniques to piezoresistive metamaterials allows for precise microarchitecture designs, thereby overcoming inherent trade‐offs between sensing performances and deformation recoverability. For example, a hierarchical lattice made of carbon aerogels enables superior elasticity and stretchability, thus maintaining electrical conductivity under both positive and negative strains.^[^ ^59^ ^]^ Besides, recent advances in programmable multi‐material AM techniques have led to the integration of functional and structural phases into complex lattice microarchitectures, such as a combined 3D lattice that includes piezoelectric, conductive, and structural elements^[^ ^557^ ^]^ (Figure 18g). The core concept in this case involves unrestricted placement of different materials into 3D lattices and the design via precise arrangement, which results in enhanced strain modes and mechanical properties. In this review, we mainly focus on lattice metamaterials enabled by 3D printing techniques, thus 4D printing techniques‐enabled programmable lattice metamaterials are only concisely discussed herein. More comprehensive discussions on 4D‐printed programmable lattice metamaterials already exist in many literatures,^[^ ^109^, ^110^, ^111^, ^112^ ^]^ and will not be discussed here in detail.

Moreover, the interaction mechanisms between mechanical and different physical properties are further summarized to reveal more effective design guidelines for multi‐physical lattice metamaterials. First, the coupling relations between acoustic and mechanical properties in lattice metamaterials, which are significantly governed by the morphology of internal microarchitectures, are critically reviewed. Different types of lattice metamaterials, namely truss, shell, and plate lattices, display varying degrees of coupling between these properties. In short, acoustic performances, such as the resonance mechanism, are primarily affected by the geometries of pores and cavities, regardless of the type of lattice metamaterials. In contrast, the mechanical properties of lattice metamaterials are determined by both the type and relative density of their internal microarchitectures, as outlined in Section 3.1. The acoustic and mechanical properties of truss and shell lattices are inherently coupled due to the limitations in structural customization. For truss lattices, the geometries of pores and cavities are constrained by the thicknesses of constituent struts or the overall complexity of microarchitectures. Truss lattices typically exhibit wide cavities at low relative densities,^[^ ^437^, ^558^ ^]^ while alternating regions of narrow pores and wide cavities appear at high relative densities.^[^ ^17^, ^50^ ^]^ Besides, the relative density, which translates into the thicknesses of struts, affects the sizes of pores and cavities significantly.^[^ ^50^ ^]^ Similarly, in shell lattices, although there is no distinct structure‐property relationship, the acoustic properties are also directly affected by the lattice geometry.^[^ ^435^, ^436^ ^]^ Attempts to alter mechanical properties in these structures, such as modifying the strut or shell thickness, relative density, or structural morphology, inevitably affect the sound absorption capability, as these adjustments simultaneously change the acoustically required geometries of lattices. In contrast, plate lattices offer a larger design freedom with the potential capability of decoupling between mechanical and acoustic properties.^[^ ^17^, ^432^, ^559^, ^560^ ^]^ This increased flexibility arises from the ability to introduce pores independently from lattice microarchitectures or relative densities. The pores can be strategically placed at various locations, such as the nodes,^[^ ^17^ ^]^ edges,^[^ ^559^ ^]^ or faces^[^ ^432^, ^560^ ^]^ of constituent plates, thereby enabling tailored modifications in sound absorption capabilities without inadvertently affecting their mechanical performances. To overcome limitations in the coupling of acoustic and mechanical properties in truss, shell, and plate lattices, hybrid lattice metamaterials, which typically integrate perforated plates with supporting structures underneath, serve as a potential design strategy to decouple acoustic from mechanical properties.^[^ ^433^, ^561^ ^]^ This design strategy allows for the independent optimization of acoustic properties, such as resonance frequency and absorption intensity, by adjusting the pore diameters, plate thicknesses, and cavity depths. Meanwhile, the supporting structures beneath the plates can be tailored to provide specific mechanical properties, such as elasticity, strength, and plastic properties, without altering the acoustic behaviors.

Albeit being governed by totally different mechanisms, the EM/optical and mechanical properties of lattice metamaterials are both significantly affected by the lattice design. Typically, EM properties are more dependent on the intrinsic EM characteristics of constitutive materials, while the optimization of lattice microarchitectures can further expand their EM wave response capabilities. For instance, by tuning the unit cell sizes of TPMS shell lattices, such as Schwarz‐P and Schoen‐G lattices, the impedance matching between the lattice and air can be optimized to promote multiple internal reflections within their internal microarchitectures. This mechanism can facilitate the near‐perfect EM wave absorption and minimize the energy loss.^[^ ^462^, ^562^ ^]^ However, given the same feature size, such as wall thickness, the changes in unit cell size inevitably affect their mechanical properties, primarily due to the alterations in relative density and deformation mechanism. The relative density decreases with an increase in unit cell size, resulting in a reduction in stiffness and strength. Conversely, a reduction in unit cell size can densify the lattice and enhance its stiffness and strength, while at the cost of compromising the lightweight design potential. In practical applications, a direct interdependence exists between the EM and mechanical properties of the lattices that enhancing the EM performances may inadvertently compromise the structural integrity. Besides, beyond adjustments in unit cell size, the EM properties of lattice metamaterials can also be enhanced by introducing more intricate microarchitectures. For example, graded G‐type shell lattices can achieve tuneable EM properties across multiple frequency bands (e.g., X, Ku, and C bands) through orientation and gradient adjustments.^[^ ^464^, ^563^ ^]^ Simultaneously, shell lattices generally offer high specific stiffness and strength while maintaining lightweight microarchitectures, due to their efficient stress distributions and smooth surfaces that minimize stress concentrations. In such cases, the appropriate design of lattice microarchitectures can harmonize EM and mechanical properties, thus allowing for a mutual enhancement. However, an excessive focus on optimizing EM performances by over‐complicating lattice microarchitectures may result in diminished mechanical properties. More specifically, the increased specific surface area of intricate lattices enhances the EM wave absorption but may render the lattices more susceptible to mechanical failure, particularly under dynamic loading conditions.^[^ ^465^ ^]^ Moreover, the selection of constitutive materials is also critical in tailoring the EM performances of lattice metamaterials. The emerging material systems not only optimize the EM performances but also enhance the mechanical robustness of lattices. For instance, the integration of polymer‐derived ceramics with lattice microarchitectures leverages the ceramics' intrinsic EM parameters to optimize the overall EM performances while utilizing their inherent high strength to achieve exceptional mechanical stability.^[^ ^459^, ^564^ ^]^ Besides, incorporating micro‐ and nano‐scale conductive fillers, such as MXene and carbon nanotubes, into aerogel lattice metamaterials can not only enhance the conductivity and EMI shielding effectiveness but also contribute to an overall lattice integrity due to multiscale reinforcement effects.^[^ ^565^ ^]^ However, adding certain fillers that do not provide reinforcements, such as liquid metals, may enhance the EM properties while compromising the mechanical stability, since the introduced microstructural defects may result in stress concentrations and reduce the mechanical robustness.^[^ ^470^ ^]^ Overall, factors such as unit cell size, geometry, and material selection collectively affect both the EM and mechanical properties of lattice metamaterials, and the decoupling of these effects in the design process is challenging. By employing adaptive optimization strategies, such as topology optimization, it is promising to fine‐tune the lattice microarchitectures to achieve simultaneous enhancement of their EM and mechanical properties. This synergistic enhancement can effectively broaden the potential of EM lattice metamaterials for applications in stealth technologies, EMI shielding, and aerospace structural components.

The coupling relations between the thermal and mechanical properties of lattice metamaterials depend on the form of heat transfer, i.e., conduction, convection, and radiation. Typically, the thermal conductivities of lattice metamaterials are not primarily affected by the type of their internal microarchitectures, such as plates, trusses, or shells.^[^ ^53^, ^55^ ^]^ Instead, their thermal conductivities are mainly determined by the relative density and pore architectures (the size, shape, and orientation of pores), since pores on one side of the unit cell alters the conductive heat transfer throughout the other sides.^[^ ^53^, ^55^ ^]^ The effective thermal conductivity of a specific type of lattices increases with the relative density, exhibiting a consistent trend to their mechanical properties, including stiffness, strength, specific energy absorption, and others.^[^ ^53^, ^55^, ^501^, ^502^ ^]^ More importantly, the utilization of FGLs can tune the heat flow and improve the thermal conductivity and heat flow rate significantly, which serves as an efficient design approach to enhance the thermal and mechanical properties of lattice metamaterials simultaneously.^[^ ^53^, ^525^ ^]^ In contrast, the convective properties of lattice metamaterials are significantly affected by the type of their internal microarchitectures, in which a trade‐off exists between the convective heat transfer rate and pressure drop.^[^ ^496^, ^502^, ^505^, ^507^ ^]^ Plate lattices typically possess more exceptional convective rates but higher pressure drops;^[^ ^505^ ^]^ conversely, truss lattices exhibit lower pressure drops but inferior convective rates.^[^ ^496^ ^]^ In comparison, shell lattices achieve a reasonable balance between the convective rate and pressure drop, thus exhibiting the best overall thermal performance.^[^ ^502^, ^507^ ^]^ The decrease in the strut diameters of truss lattices or wall thicknesses of plate/shell lattices tends to allow more air to invade their micro‐pores and can enhance the convective rates, while at the cost of decreasing conductive rates due to reducing surface areas and solid volumes.^[^ ^566^ ^]^ Besides, the mechanical properties of the lattices also decrease with decreasing relative densities, posing a trade‐off between their conductive/convective heat transfer rates and mechanical properties.^[^ ^566^ ^]^ Moreover, albeit being negligible in lattice metamaterials with lower porosities, the radiative heat transfer plays a significant role in the effective thermal conductivities of lattices with higher porosities.^[^ ^496^ ^]^ The radiative heat transfer rates of lattice metamaterials generally exhibit a consistent trend to their conductive rates and mechanical properties, which tend to increase with increasing relative densities or decreasing porosities.^[^ ^566^ ^]^

#### 3.3.3 Physicochemical Properties

Furthermore, in addition to multi‐physical properties, the other properties of lattice metamaterials also include electrochemistry,^[^ ^57^, ^567^ ^]^ photochemistry,^[^ ^568^ ^]^ chemical reactivity,^[^ ^178^, ^569^, ^570^ ^]^ as well as moisture, pH, and solvent responsiveness,^[^ ^571^ ^]^ amongst many other physicochemical properties.^[^ ^572^ ^]^ By exploring the interaction mechanisms among different mechanical/physical and chemical properties and fine‐tuning their microarchitecture configurations, lattice metamaterials can simultaneously enhance these multi‐physical and physicochemical properties. Typically, the enhancement of pure chemical properties is closely related to the chemical compositions of materials rather than the internal microarchitectures of lattice metamaterials, hence well beyond the scope of multi‐physical properties enabled by the design of lattice microarchitectures in this review. More specifically, for hydrogel materials with self‐repairing properties, the design in lattice form can enable further enhancement of their homogenized mechanical properties and self‐healing capabilities. This effect was demonstrated by integrating robust, self‐healing, and recyclable properties into polymer composites via innovative AM techniques^[^ ^570^ ^]^ (Figure 18h). Different lattice designs typically exhibit different stress responses and deformation behaviors, which are crucial for applications requiring durability and flexibility under external loads. Moreover, AM techniques offer flexibility in incorporating active materials into lattice metamaterials, thereby enabling diverse chemical responses, such as gas sensing and electrocatalysis.^[^ ^569^, ^573^ ^]^ Recent studies have also incorporated moisture‐responsive materials, such as humidity‐sensitive cholesteric liquid crystal oligomers, into lattice metamaterials. For example, the behaviors of adaptive hydrochromic coatings atop 3D‐printed structures are attributed to the unique alignment of cholesteric mesophases in the ink that exhibits a significant bathochromic shift in reflection upon exposure to water.^[^ ^574^ ^]^ Furthermore, the other multi‐physical and physicochemical properties of lattice metamaterials, including magneto‐thermomechanical,^[^ ^575^ ^]^ magneto‐photothermal,^[^ ^576^ ^]^ and photo‐electrochemical,^[^ ^577^ ^]^ etc., have also received increasing attention in recent years.

## 4 Multifunctional Applications of Lattice Metamaterials

In addition to the most widely studied applications as energy absorbers and vibration isolators, this section summarizes recent advancements in the multifunctional applications of lattice metamaterials, such as sound absorbers, insulators, and manipulators, sensors, actuators, and soft robots, thermal management, invisible cloaks, biomedical implants, etc. All the applications reviewed in this section must involve the coupling of properties derived from two different or even more disciplines: typically, a mechanical property and another physical property at least. The interaction mechanisms between different properties and the corresponding design principles of lattice metamaterials for these multifunctional applications are also discussed.

### 4.1 Sound Absorbers, Insulators, and Manipulators

Lattice metamaterials have emerged as highly advantageous alternatives to traditional sound absorbers,^[^ ^578^ ^]^ such as perforated panels, foams, fabrics, etc. This stems from their operation based on the same fundamental principles of sound absorption, using analogous acoustic geometries, but possessing greater design versatility. Consequently, lattice metamaterials exhibit comparable sound absorption characteristics, but their inherent design versatility extends opportunities for tailorable optimization and customization, thereby facilitating the achievement of enhanced sound absorption performances.

**Figure 19:**
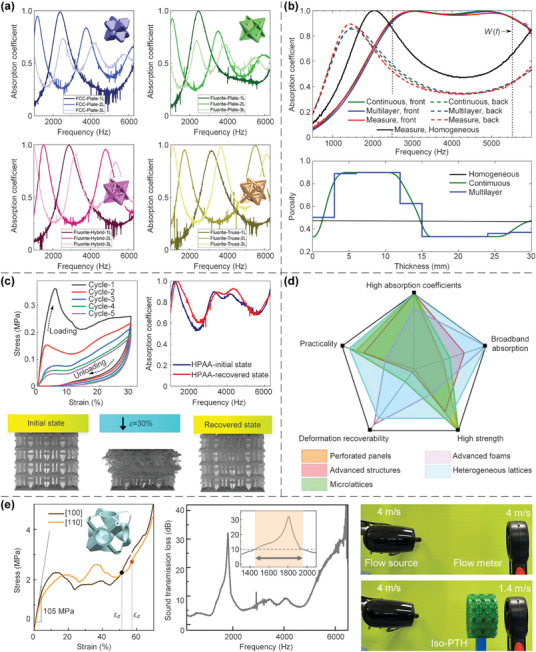
Multifunctional applications of lattice metamaterials as sound absorbers and isolators. The sound absorption coefficient curves of lattice metamaterials with a) distinct absorption resonance peaks, and b) smooth and gradual curves. Reproduced with permission.^[^ ^17^ ^]^ Copyright 2021, Wiley. Reproduced with permission.^[^ ^439^ ^]^ Copyright 2019, AIP. A heterogeneous sound‐absorbing lattice with high toughness, revealing c) high recovery under cyclic compression, minimal changes to post‐recovery absorption coefficients, and d) potential as an advanced sound absorber. Reproduced with permission.^[^ ^49^ ^]^ Copyright 2021, Wiley. e) Multifunctional lattice metamaterials for simultaneous sound insulation and structural applications, with a maximum sound attenuation of 32 dB at 1810 Hz. Reproduced with permission.^[^ ^60^ ^]^ Copyright 2023, Elsevier.

Extensive investigations have been performed to explore the sound‐absorption properties of a wide spectrum of lattice metamaterials, with different unit cell sizes and wall thicknesses. The unit cells examined are up to 10 mm in size, and the wall thicknesses, which depend on the layer number and unit cell size, typically vary from 20 to 60 mm. Within these ranges of geometric parameters, the frequency of lattices generally ranges from 1000 to 6000 Hz. Analogous to traditional sound‐absorbing materials, lattice metamaterials exhibit distinct sound‐absorption coefficient profiles, owing to their unique structural morphologies. For example, lattices with certain configurations exhibit sound‐absorption curves characterized by discernible resonant peaks (**Figure** **19**a), akin to perforated panels.^[^ ^17^ ^]^ Conversely, optimized FGLs exhibit absorption curves with gradual and smooth transitions (Figure 19b), resembling the behavior of foams and fabrics.^[^ ^439^ ^]^ The difference in the absorption curves of different lattices is attributed to their reliance on identical underlying sound‐absorption principles as traditional materials. Furthermore, lattice metamaterials can also be devised based on mechanism‐driven structural optimization, which generally results in robust and broadband sound‐absorption capabilities.

The introduction of distinctive features into sound‐absorbing lattices has been explored using specialized materials and innovative designs. For example, the utilization of a tough polymer in conjunction with an NPR design has yielded sound‐absorbing lattices capable of deformation recovery.^[^ ^49^, ^433^ ^]^ One reported lattice exhibited both high strain recovery (≈30%) and high strength (∼370 kPa), as demonstrated in cyclic compression tests^[^ ^49^ ^]^ (Figure 19c). These lattices can be regarded as strong, lightweight, and pseudo‐impact‐deformation‐reusable sound absorbers, as they display minimal alterations in sound‐absorption performances following impact and subsequent recovery. These lattices outperform various traditional sound absorbers, such as polyurethane foams and perforated panels, and are thus emerging as promising candidates for alternative advanced sound absorbers (Figure 19d).

In addition, lattice metamaterials also show advantageous potential for multifunctional sound insulation and structural applications. As mentioned previously, lattice metamaterials can achieve superior lightweight and energy absorption capabilities. Therefore, lattice sound insulators can also serve as effective architected materials simultaneously, unlike sonic crystals which only serve as sound barriers. For example, a hybrid plate and tube lattice has been optimized to achieve isotropic elasticity while harnessing dual sound insulation mechanisms,^[^ ^60^ ^]^ in which a peak sound transmission loss of 32 dB is observed in the case of three unit cells (unit cell size: 20 mm) throughout the thickness, with a high attenuation over 10 dB spanning a wide frequency range (1440–1950 Hz) (Figure 19e). Besides, another notable sound attenuation band is also observed beyond 5000 Hz. Additionally, due to its tubular design, the lattice maintains a high air ventilation, retaining 35% of the airflow (Figure 19e). Recently, an inertia design framework based on positioning microspheres has been proposed to tune the ultrasound propagation of 3D microscale metamaterials, which can achieve tuneable quasi‐static stiffness (up to 75%) and dynamic longitudinal wave velocities (up to 25%), while maintaining identical material density.^[^ ^579^ ^]^ Conversely, the wave propagation characteristics of lattice metamaterials can be utilized for non‐destructive extraction of dynamic linear characteristics, omnidirectional elastic information, damping characteristics, and defect quantification.^[^ ^580^ ^]^

### 4.2 Sensors, Actuators, and Soft Robots

Lattice metamaterials can enable numerous multi‐responsive applications in which their traditional rigid counterparts are not applicable, such as in sensors, actuators, and soft robots with programmability and adaptability. Typically, lattice metamaterials utilize the synergy between constitutive material properties and microarchitecture topologies to sense external stimuli. Besides, multiple energy inputs can be converted into mechanical outputs via tethered and untethered approaches. Therefore, lattice metamaterials can significantly enhance sensing and actuating capabilities in various applications.

**Figure 20:**
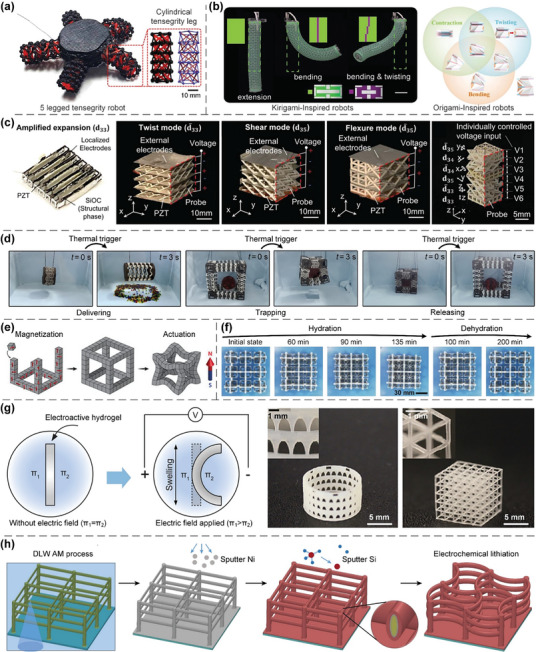
Multifunctional applications of lattice metamaterials as sensors, actuators, and soft robots. a) A walking starfish robot with a 3D‐printed body, five identical legs composed of cylindrical tensegrity, and two motors mounted on each leg for bending and contraction. Reproduced with permission.^[^ ^581^ ^]^ Copyright 2020, AAAS. b) Kirigami‐ and origami‐inspired robots can mimic target shapes with contraction/extension, bending, twisting, and their combined motion modes via pneumatic actuation. Reproduced with permission.^[^ ^583^ ^]^ Copyright 2020, Wiley. Reproduced with permission.^[^ ^584^ ^]^ Copyright 2023, Springer. c) Robotic lattices with the ability to exhibit multiple‐degree‐of‐freedom motions and amplify strains under the stimuli of an electric field. Reproduced with permission.^[^ ^557^ ^]^ Copyright 2022, AAAS. d) A cylindrical bi‐material lattice with an initial open‐cell configuration was utilized to demonstrate object delivery and release, and 3D hierarchical bi‐material lattices were utilized to capture and release spherical objects upon exposure to heat source. Reproduced with permission.^[^ ^323^ ^]^ Copyright 2023, Elsevier. e) 3D lattices with programmable shape variations under applied magnetic fields due to their designed magnetization patterns. Reproduced with permission.^[^ ^590^ ^]^ Copyright 2021, AAAS. f) The programmable shape morphing of 3D bi‐material lattices under hydration and dehydration. Reproduced with permission.^[^ ^591^ ^]^ Copyright 2020, Elsevier. g) Electroactive hydrogel actuator bends towards the cathode by the swelling difference of 3D‐printed structures. Reproduced with permission.^[^ ^592^ ^]^ Copyright 2018, ACS. h) Electrochemically driven reconfigurable microlattices. Reproduced with permission.^[^ ^593^ ^]^ Copyright 2019, Springer.

Tethered lattice robots are usually actuated through passive deformations induced by motion transmitters attached to external motors (for example, cables and tendons), fluidic pressure variations in the hollow channels of their soft bodies, and electrically driven shape variations. Passive traction lattices can achieve complex deformations and movements via the tension transmitted by tendons from external motors connected to different parts of soft structures. Tendon‐driven actuations are especially well‐suited for soft machines that possess discontinuous or architected bodies and are thus unable to utilize fluidic pressures. These machines can deliver rapid and precise deformations with substantial output forces, while maintaining the overall compliance of structures. For instance, a walking starfish robot was developed, which consisted of a 3D‐printed body, five identical legs comprising cylindrical tensegrity, and two motors mounted on each leg for bending and contraction^[^ ^581^ ^]^ (**Figure** **20**a). Pressure‐controlled lattices, which can convert the pressure of fluids (gases or liquids) into force and displacement outputs flexibly, are reusable and easy for 3D printing and can provide large deformations. A recent study investigated the alterations in the mechanical responses of a soft lattice under pressure gradients, in which the concept of pneumatic actuation was integrated into the lattice pattern design and the combination of NPR and PPR lattices resulted in programmable bending effects.^[^ ^582^ ^]^ Additionally, kirigami‐inspired robots can mimic target shapes with extension, bending, and twisting deformations by uniform and non‐uniform designs upon pressurization^[^ ^583^ ^]^ (Figure 20b). Besides, contraction/extension, bending, twisting, and any combination of these motion modes of origami‐inspired structures can be realized via pneumatic actuation^[^ ^584^ ^]^ (Figure 20b). Moreover, electrically driven lattices can convert electric energy into force outputs and shape variations in lattice metamaterials with smart constitutive materials, including shape memory materials, piezoelectric materials, and dielectric elastomers. For example, a category of robotic lattices were engineered with the ability to exhibit multiple‐degree‐of‐freedom motions and amplify strains in specified directions under the stimuli of electric fields (and vice versa), thereby enabling programmable motions with self‐sensing and feedback control.^[^ ^557^ ^]^ These lattices were formed by weaving an interconnected network of conductive, piezoelectric, and structural elements into 3D lattices intricately (Figure 20c). Overall, tethered lattice robots, which are activated by either onboard electronics or soft pumps, exhibit advantageous properties, such as substantial force outputs and flexible deformations.

Nevertheless, soft robots triggered by untethered stimuli are preferred in numerous specific applications. The stimuli sources of untethered soft robots can be classified as acoustic, temperature, light, magnetic, and chemical stimuli. Given the capability of deep penetration into soft lattices, fluidic media, and biological tissues, acoustic waves serve as a feasible mechanical actuation source. The efficient conversion of acoustic energy into mechanical motion necessitates mechanical resonance, which is generally achieved via incorporating oscillatory elements, such as gas bubbles, or flexible and sharp microarchitectures, into robot bodies for generating propulsion forces.^[^ ^585^, ^586^, ^587^, ^588^ ^]^ Besides, temperature variations in environments, whether in air or liquids, can activate thermo‐responsive soft lattices, such as those with two constitutive materials with different CTEs or different temperature‐dependent moduli, shape‐memory materials, hydrogels, and liquid crystal elastomers. For example, two 3D‐printable polymers that are independent of intrinsic shape memory effects and compositional alterations were exploited to generate robust and simplified shape memory responses in stimuli‐responsive lattices, thereby eliminating the complicated programming processes of archetypal shape memory polymers.^[^ ^323^ ^]^ The driven characteristics are the incompatibility of the temperature‐dependent moduli of different materials in interconnected double‐U units (Figure 20d). A bi‐material cylindrical structure with initial open‐cell configurations was utilized to demonstrate the delivery and release of drugs upon exposure to heat source, which could be used to accomplish targeted therapy, and 3D hierarchical bi‐material structures were utilized to capture and release spherical objects. In addition, light is another common wireless stimulus for soft robots, offering advantageous modulations with high spatial and temporal resolutions using well‐established equipment. The operational principle of synthetic light‐responsive actuators and robots typically relies on photochemical reactions and/or photothermal effects. For instance, an asymmetric bilayer comprising polyethylene and stacked graphene was devised to exhibit rapid deformation responses and programmable rolling locomotion under infrared illumination, owing to their significantly different CTEs.^[^ ^589^ ^]^ Additionally, soft lattice robots controlled by magnetic fields are typically actuated through torques or forces resulting from interactions between the magnetic properties of their constitutive materials and the regulated external magnetic fields or gradients. These robots are widely applied in minimally invasive surgery and targeted drug delivery within a confined workspace. For example, microscale building blocks comprising hard magnetic composites were devised, which can be magnetized and assembled into 3D structures.^[^ ^590^ ^]^ These structures exhibited programmable shape variations under applied magnetic fields due to their magnetization patterns (Figure 20e). Moreover, chemical stimuli, including humidity, ionic strength, and various chemical substances in liquid or vapor forms, can induce responses in stimuli‐responsive soft lattices and provide the actuation energy. For instance, hygroscopic actuators utilize the volume swelling behavior of water‐absorbing lattices with anisotropic or heterogeneous designs to realize locomotion and programmable shape morphing^[^ ^591^ ^]^ (Figure 20f). In addition, alterations in pH, ionic strength, solvent changes, the presence of solvent vapors, the byproducts of chemical reactions (such as gas and heat), and surface tension gradients can also cause actuators to respond. Furthermore, multiple physical fields or physicochemical control can achieve multi‐mode sensing and complex actuation of soft robots. For example, 3D‐printed microarchitectures with electroactive hydrogels were shown to be capable of soft robotic manipulation and locomotion^[^ ^592^ ^]^ (Figure 20g). The osmotic pressure balance between the two interfaces of electroactive hydrogel is disrupted (π_1_>π_2_) under an applied external electric field, which leads to nonuniform swelling between interfaces and enables the electroactive hydrogel to bend towards the cathode. In this case, an electrochemically driven silicon‐lithium alloying reaction can enable 3D silicon‐coated tetragonal microlattices to transform into sinusoidal patterns through cooperative beam buckling^[^ ^593^ ^]^ (Figure 20h).

### 4.3 Thermal Management

**Figure 21:**
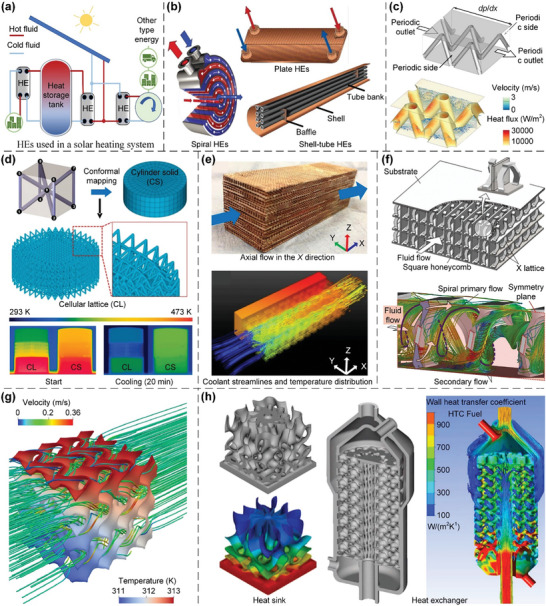
Multifunctional applications of lattice metamaterials for thermal management. a) HEs are used in a solar heating system to enhance the heat transfer efficiency. b) Spiral, plate, and shell‐tube HEs. Reproduced with permission.^[^ ^63^ ^]^ Copyright 2022, Elsevier. Reproduced with permission.^[^ ^599^ ^]^ Copyright 2021, Springer. c) Tetrahedral lattice‐frame metamaterials are devised for use as multifunctional compact HEs. Reproduced with permission.^[^ ^602^ ^]^ Copyright 2017, Elsevier. d) Boundary‐conformal lattice HEs are devised to achieve superior mechanical stiffness and strength to bear external loads and a maximal cooling capability enhancement of 68.7‐70.8% than conventional Ti‐6Al‐4V solid HEs. Reproduced with permission.^[^ ^604^ ^]^ Copyright 2021, Elsevier. e) 3D woven copper lattices are optimized to achieve enhanced mechanical stiffness, maximized flow permeability, and reduced pressure drop for use as efficient HEs. Reproduced with permission.^[^ ^605^ ^]^ Copyright 2016, Elsevier. f) The design of novel HEs in the form of X‐lattice cored rectangular honeycombs to improve the convective heat transfer efficiency. Reproduced with permission.^[^ ^64^ ^]^ Copyright 2020, Elsevier. g) Schwarz D‐type TPMS shell lattices are used as smooth HEs to enhance the heat transfer efficiency and minimize the pressure drop. Reproduced with permission.^[^ ^608^ ^]^ Copyright 2021, Elsevier. h) HEs infilled with different types of TPMS shell lattices are devised for use in aerospace turbines (realized by nTopology). Reproduced with permission.^[^ ^609^ ^]^ Copyright 2020, nTopology.

HEs, modules that transfer heat between a heat source and one or more working fluids, are a key component in various thermal management systems and widely used in air conditioning, chemical processing, renewable energy, and other fields.^[^ ^594^, ^595^ ^]^ To date, single‐phase heat transfer has been sufficiently developed in lattice metamaterials, while multi‐phase heat transfer involving evaporation or boiling remains less studied.^[^ ^595^, ^596^, ^597^, ^598^ ^]^ The operational principle of a solar heating system is shown in **Figure** **21**a, in which HEs considerably affect the overall heat transfer efficiency of the system. Typically, HEs are classified as spiral,^[^ ^63^ ^]^ plate,^[^ ^599^, ^600^ ^]^ and shell‐tube^[^ ^63^, ^599^, ^601^ ^]^ HEs (Figure 21b). By selecting appropriate HEs, the effectiveness of various energy systems, including solar thermal energy systems, can be substantially enhanced. However, as discussed, the limitations of conventional processes mean that only simple geometries, such as tubes, plates, and sheets, can be used to construct HEs, thus restricting their thermal performances. Efficiency enhancements necessitate radical design changes, such as designs that exhibit high heat transfer coefficients with an acceptable pressure drop and low leakage risk while ensuring compactness. Advancements in AM techniques and associated design strategies have enabled the development of HEs with superior thermal and mechanical performances through more freeform designs with highly complex geometries.

To enable the simultaneous achievement of superior thermal and mechanical properties, lattice metamaterials have been widely used in the design of HEs by tailoring their internal microarchitectures. For example, when used as compact HEs, tetrahedral lattice‐frame metamaterials exhibit superior stiffness‐ and strength‐to‐mass‐ratios, high surface‐area‐to‐volume ratios, and large porosities, which result in low‐pressure drop while maintaining high structural efficiency^[^ ^602^ ^]^ (Figure 21c). Additionally, a series of boundary‐conformal HEs were devised by conformally mapping BCCz truss lattices^[^ ^603^ ^]^ to a hexahedral finite element mesh^[^ ^604^ ^]^ (Figure 21d). These HEs possess superior mechanical stiffness and strength to bear external loads in working environments and exhibit a maximal cooling capability enhancement of 68.7‐70.8% than conventional Ti‐6Al‐4V solid HEs. Besides, 3D woven copper lattices possess higher thermal conductivities, higher surface‐area‐to‐volume ratios, and a more regular distribution of micropores, and can be devised in a standard architecture form to achieve a higher heat transfer efficiency.^[^ ^605^ ^]^ However, the standard architecture uses a larger volume of solid materials and generally results in a larger pressure drop, thus a topologically optimized architecture is proposed to achieve enhanced mechanical stiffness, maximized flow permeability, and reduced pressure drop, albeit at the cost of a more uneven distribution of temperature^[^ ^605^ ^]^ (Figure 21e). Furthermore, modified turbulence patterns in X‐lattice sandwich panels show that the high turbulent kinetic energy induced by intensified flow mixing and increased local flow velocity can enhance the heat transfer efficiency by 2.3 times^[^ ^64^, ^606^ ^]^ (Figure 21f). In addition, corrugated plates, such as those with wavy, chevron, washboard, or cross‐corrugated patterns, were often used to increase surface areas and promote turbulent flows.^[^ ^607^ ^]^

Moreover, TPMS shell lattices possess superior stiffness and strength, large surface‐area‐to‐volume ratios, and smooth surfaces, which enable their applications in high‐performance HEs and heat sinks. The curved and smooth surfaces of TPMS shell lattices serve as a vortex generator that can disturb boundary layer flows, thereby increasing heat transfer coefficients by mixing flows between the wall and core regions.^[^ ^595^ ^]^ More specifically, the utilization of Schwarz D‐type TPMS shell lattices can effectively enhance the heat transfer efficiency between the heat source and flowing air. In comparison to an empty channel, the amount of heat removed from the heat source only results in a very slight pressure drop penalty^[^ ^608^ ^]^ (Figure 21g). The excellent applicability of HEs comprising TPMS shell lattices and the automation of design to achieve superior thermal and mechanical performances have been demonstrated by more detailed numerical simulations^[^ ^73^, ^609^ ^]^ (Figure 21h). Compared with circuit HEs, TPMS‐based HEs exhibit 16–120% increase in Nusselt number, and 15–100% enhancement in overall thermal performance. Specifically, G‐ and D‐type TPMS lattices induce more complex vortices than circuit HEs in streamwise locations.^[^ ^610^ ^]^ The different flow behaviors within TPMS‐based heat sinks show that D‐type solid‐networks with a high tortuosity exhibit higher convective heat transfer coefficients than G‐type solid‐networks, albeit at the cost of larger pressure drops. In addition, expanding the channel size in the flow direction can decrease the pressure drop.^[^ ^611^ ^]^ The morphological analysis of a variety of TPMS shell lattices were conducted to identify the relationship between heat transfer performances and geometric parameters. The results indicate that P‐type TPMS lattices achieve the highest heat transfer coefficient and lowest flow resistance.^[^ ^612^ ^]^ Besides, the simulations of pure conduction and natural convection under isothermal conditions have demonstrated that TPMS shell lattices also provide enhanced thermal performances.^[^ ^509^ ^]^ Generally, P‐type TPMS shell lattices achieve the highest conductivity, and compared with smaller cells, larger cells possess higher conductivity due to greater intra‐cell convective heat transfer.^[^ ^502^ ^]^ The effects of pore architectures on thermal conductivity have also been investigated, revealing that FGLs can efficiently tune heat flows to control the temperature.^[^ ^53^ ^]^ Other studies have also demonstrated the superior heat transfer and mechanical performances of HEs comprising TPMS shell lattices.^[^ ^613^, ^614^, ^615^ ^]^

### 4.4 Invisible Cloaks

**Figure 22:**
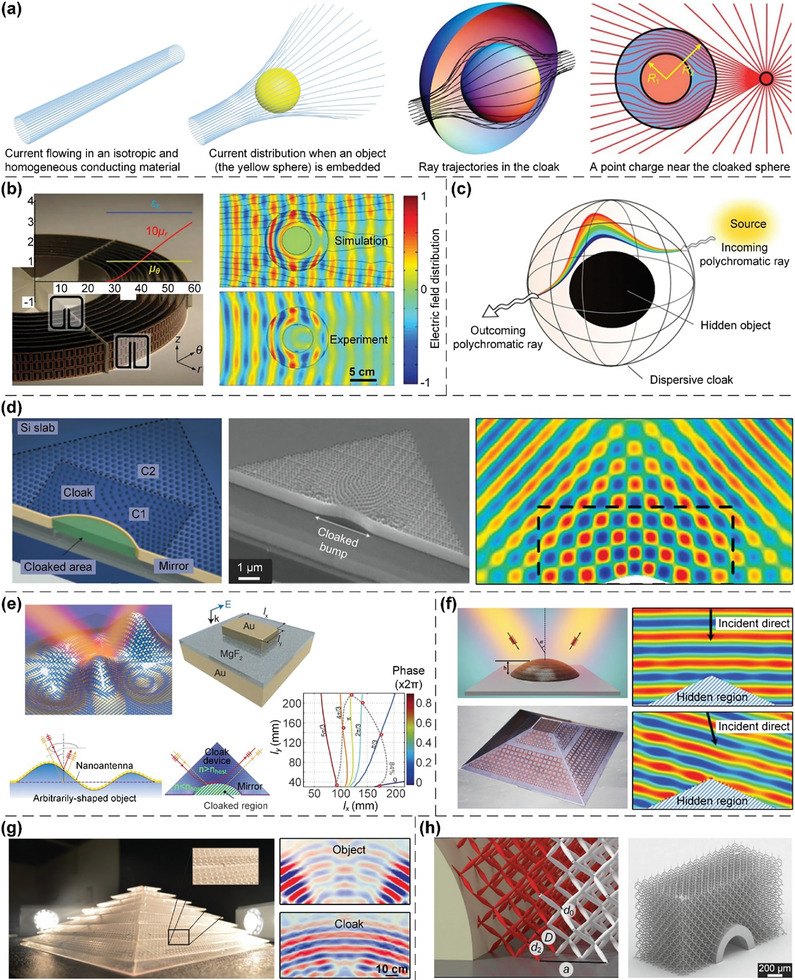
Multifunctional applications of lattice metamaterials as invisible cloaks. a) Illustration of EM invisible cloaks, in which the current flowing and EM ray trajectory in the cloaking shell are smoothly redirected around the object, rendering it invisible to outside observers. Reproduced with permission.^[^ ^619^ ^]^ Copyright 2013, Wiley. Reproduced with permission.^[^ ^52^ ^]^ Copyright 2006, AAAS. b) The experimental realization of a microwave cloaking shell composed of split‐ring resonators, together with the plot of material parameters and the numerical and experimental results that illustrate the reconstruction of incident waves after passing the cloak. Reproduced with permission.^[^ ^65^ ^]^ Copyright 2006, AAAS. c) Illustration of the rainbow effect within a frequency‐dispersive invisible cloak, in which the polychromatic ray is broken into different spectral constituents inside the cloak. Accordingly, each color follows its own path to avoid passing through the central region, and external observers can neither notice the cloak nor the object inside. Reproduced with permission.^[^ ^617^ ^]^ Copyright 2019, Springer. d) The carpet design of an optical cloak and the scanning electron microscope image of a fabricated part, in which the cloaked region (marked in green) resides below the reflecting bump (carpet) and can conceal any arbitrary object. Reproduced with permission.^[^ ^66^ ^]^ Copyright 2009, Springer. Reproduced with permission.^[^ ^622^ ^]^ Copyright 2008, APS. e) An ultrathin skin cloak enabled by a metasurface made of nanoantennas (gold blocks) over an arbitrary object. Reproduced with permission.^[^ ^623^ ^]^ Copyright 2015, AAAS. f) Illustration of a 3D full‐polarization metasurface carpet cloak and the reflected transverse electric field distribution for normal and 15° incident obtained via numerical simulations. Reproduced with permission.^[^ ^624^ ^]^ Copyright 2016, Wiley. g) A 3D acoustic ground cloak for the sound in air and the measured acoustic pressure field for objects with and without the cloak. Reproduced with permission.^[^ ^627^ ^]^ Copyright 2014, Springer. h) A pentamode lattice‐based elasto‐mechanical unfeelability cloak was fabricated by dip‐in DLW. Reproduced with permission.^[^ ^35^ ^]^ Copyright 2014, Springer.

The mechanisms that allow metamaterials to regulate the propagation of EM waves via the design of EM parameter distributions, including effects such as space distortion and negative refraction, can guile EM/optical waves to bypass spaces enclosed by cloaks and reconstruct them into the incident shape, thereby achieving perfect stealth functionality based on the principles of transformation optics.^[^ ^616^ ^]^ The theory of transformation optics has been validated through numerical simulations, especially in the intense near and far fields of an EM source^[^ ^617^, ^618^ ^]^ Based on this approach, various illusion devices have been devised, such as the direct current and EM invisible cloak^[^ ^52^, ^619^ ^]^ (**Figure** **22**a). A spherical shell served as an invisible cloak, preventing incident waves from impinging on any object inside the shell and keeping the external field unperturbed, which made the object invisible to outside observers. By means of resonances, the first cylindrical cloaking shell comprising split‐ring resonators is experimentally realized to satisfy the required permittivities and permeabilities for achieving cloaking functionality, which agrees well with simulation results^[^ ^65^ ^]^ (Figure 22b). Additionally, an invisible device operating below the quasi‐static limit was reported, in which electrical impedance tomography was unable to detect a certain region rendered by anisotropic conductivity.^[^ ^620^ ^]^

In optical cloaking, the path of incoming ray deviates from the central region after negative refraction, rendering objects invisible to certain frequencies of EM radiation^[^ ^617^ ^]^ (Figure 22c). However, the aforementioned designs utilized metallic lattices and were difficult to be scaled down to the visible spectrum. Recent studies have revealed the possibility that cloaking effects may be exhibited by photonic and plasmonic crystal lattice metamaterials. The initially examined metamaterials in this context include periodic microarchitectures composed of metal wires in a cylindrical dielectric lattice, where the spheroidal silver wires are all perpendicular to the inner and outer interfaces of the cylinder.^[^ ^621^ ^]^ The experimental validation of EM cloaking at near‐visible wavelengths was pioneered with a quasi‐conformal mapping approach using dielectric materials^[^ ^66^ ^]^ (Figure 22d). Subsequently, an optical ‘carpet’ cloak that overcame the limitations of metallic metamaterials was fabricated^[^ ^622^ ^]^ (Figure 22d), which prevented the interaction of objects with EM waves and rendered them invisible. The focused‐ion‐beam milling was utilized to fabricate the carpet from 2D cylindrical lattices on a silicon‐on‐insulator framework. The device was divided into a uniform pattern and a variable pattern, enabling invisibility within a wavelength range from 1.4 µm to 1.8 µm. Typically, due to the limited resolution of the fabrication technique, the roughness of these devices in the visible range may lead to an increase in scattering losses at higher frequencies. Metasurfaces, artificial sheet materials containing subwavelength features on the surface that manipulates the phase of scattered waves, provide a new cloaking strategy to cloak large objects. For example, an ultrathin (80 nm thick) invisible skin cloak consisting of a metasurface with distributed phase shifts can redirect the visible light of 730 nm and render any wrapped object invisible^[^ ^623^ ^]^ (Figure 22e). Additionally, a full‐polarization 3D carpet cloak that maintains the phase and amplitude is achieved by using metasurfaces, allowing for the manipulation of the amplitude, phase, and polarization of light^[^ ^624^ ^]^ (Figure 22f). Furthermore, another ‘woodpile’ lattice structure was devised and fabricated via electron‐beam lithography, which exhibited superior overall performance in invisible cloaking and integral imaging.^[^ ^625^ ^]^

Drawing inspiration from the invisible theory in optics, lattice metamaterials have also been explored for various fields beyond invisibility, such as elastic, acoustic, mass diffusion, heat, and hydrodynamic cloaking.^[^ ^425^ ^]^ In dynamic mechanics, elastic waves are mechanical waves that propagate as transverse or longitudinal waves in solids. In contrast, transverse waves cannot propagate in fluids since shear deformations are prohibited, thus mechanical waves can only propagate as longitudinal waves, known as acoustic waves in fluids.^[^ ^626^ ^]^ For example, a square cloaking pyramid set upon a reflective surface is used to conceal an inside object to demonstrate a 3D acoustic ground cloak for sound in the air^[^ ^627^ ^]^ (Figure 22g). A 5 mm air‐filled cubic unit cell contains a 1.6 mm thick acrylic plate with a 0.85 mm perforated hole in the center, and is devised to function at the frequency of 3 kHz in the pyramid. A nearly omnidirectional sound pulse is sent toward the pyramidal cloak tip, demonstrating an almost perfect 3D cloaking functionality. In quasi‐static mechanics, a pentamode lattice‐based elasto‐mechanical core‐shell unfeelability cloak was introduced^[^ ^35^ ^]^ (Figure 22h). The measured displacement fields of the dip‐in DLW fabricated polymer‐based pentamode lattice samples exhibit a very good cloaking performance for an arbitrary object inside the rigid hollow cylinder. Based on this concept; by wrapping a homogeneous isotropic shell around the lattice, the core–shell geometry can appear as elastic as its isotropic‐compliant homogeneous surroundings. Therefore, the geometry can appear to be the same as the environment with respect to compressive and shear loads.^[^ ^35^ ^]^ For perfect elastic cloaking, pentamode lattices that possess a pseudo‐elasticity tensor with non‐zero eigenvalues for pressure stimuli are devised^[^ ^628^, ^629^ ^]^ Additionally, pentamode lattices with a large bulk‐to‐shear moduli ratio (*K*/*G* = 1000) have been experimentally realized in both macroscopic and microscopic fields recently.^[^ ^630^ ^]^ Furthermore, another modified FCC pentamode lattices are proposed to achieve tailorable mass density and bulk modulus independently.^[^ ^631^ ^]^

### 4.5 Biomedical Implants

Lattice metamaterials hold great promise for medical applications, especially as biomedical implants. The global market for additively manufactured lattice metamaterials in biomedical industries is going to reach US$147 billion by 2027.^[^ ^73^ ^]^ The most common applications include bone reconstruction,^[^ ^632^ ^]^ cardiovascular stents,^[^ ^633^ ^]^ heart valves,^[^ ^634^ ^]^ dental implants,^[^ ^635^ ^]^ tissue regeneration,^[^ ^636^ ^]^ and drug delivery.^[^ ^637^ ^]^ Typically, the design of lattice metamaterials for biomedical applications involves an intricate interaction of structural, mechanical, and biological considerations. For example, biocompatibility and bio‐functionality are required in bone tissue scaffolds to facilitate the cell seeding, ingrowth, and tissue regeneration.^[^ ^638^ ^]^ Additionally, their mechanical properties should be matched with those of natural bones to prevent stress shielding or load redistribution.^[^ ^72^ ^]^ Lattice metamaterials exhibit large surface areas and high interconnected porosities, thereby satisfying the abovementioned multifunctional biomechanical requirements. Their large surface areas facilitate initial cell seeding and promote interactions between scaffolds and external environments, and their interconnected porosities ensure the permeability of implants, which is crucial for cell and tissue ingrowth, nutrient and waste transport, and vascularization.^[^ ^73^ ^]^ Nevertheless, their mechanical properties, including stiffness and strength, tend to decrease with increasing porosities. As a result, their structural design must be optimized to achieve a balance between biological and mechanical properties required for biomedical applications.

**Figure 23:**
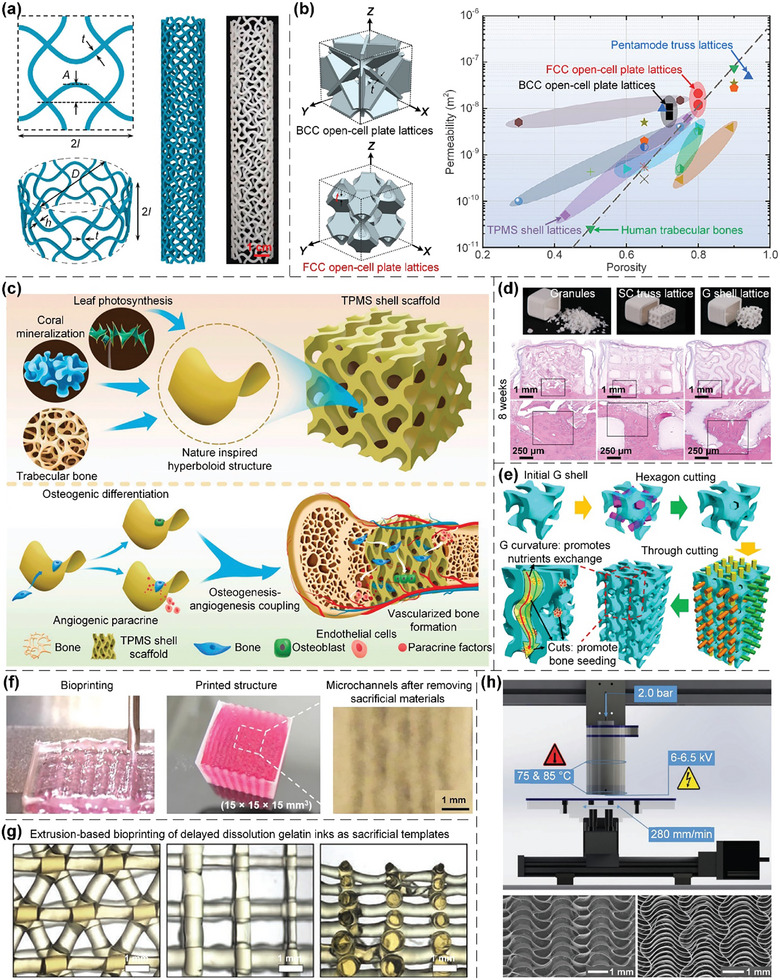
Multifunctional applications of lattice metamaterials as biomedical implants. a) A cardiovascular stent with NPR tubular structures. Reproduced with permission.^[^ ^633^ ^]^ Copyright 2022, Elsevier. b) BCC and FCC plate‐based Ti‐6Al‐4V scaffolds with mechanical performances akin to those of natural bones. Reproduced with permission.^[^ ^640^ ^]^ Copyright 2022, Elsevier. c) Schematic illustration of 3D‐printed biomimetic TPMS scaffolds that promote vascularized bone formation. Reproduced with permission.^[^ ^68^ ^]^ Copyright 2022, PNAS. d) In‐silico optimized G‐type TPMS scaffolds that show enhanced formation and colonization of new bones than granule bone substitutes and SC truss lattices in rat calvarium models after 8 weeks. Reproduced with permission.^[^ ^642^ ^]^ Copyright 2022, Wiley. e) Hierarchical design of G‐type TPMS shell lattices with additional pores on walls to promote both mass transport and cell seeding. Reproduced with permission.^[^ ^643^ ^]^ Copyright 2021, Elsevier. f) Bioprinting of human skeletal muscle constructs with excellent in‐vitro cellular viability, alignment, and maturation. Reproduced with permission.^[^ ^646^ ^]^ Copyright 2018, Springer. g) Truss lattices by extrusion‐based bioprinting with programmable delayed dissolution. Reproduced with permission.^[^ ^67^ ^]^ Copyright 2023, Wiley. h) Heart valve tissue engineering enabled by melt electrowriting. Reproduced with permission.^[^ ^634^ ^]^ Copyright 2019, Wiley.

Lattice metamaterials for biomedical applications most commonly comprise truss‐, plate‐, and shell‐based microarchitectures. For example, Ti‐6Al‐4V scaffolds with various truss‐based lattices, including Schwarz D‐type and tetrahedral truss lattices, were fabricated, among which Schwarz D‐type truss‐based scaffolds were found to obtain superior mechanical properties and promote the formation of new bones in vivo.^[^ ^639^ ^]^ Additionally, 3D‐printed auxetic tubular truss lattices with enhanced mechanical resilience can be used as an alternative stent for treating coronary artery disease because stents can effectively open narrow coronary arteries to improve the blood flow to the heart^[^ ^633^ ^]^ (**Figure** **23**a). Plate lattices possess more exceptional specific stiffness and strength than truss lattices,^[^ ^10^ ^]^ in which BCC and FCC plate‐based Ti‐6Al‐4V scaffolds have been devised to obtain mechanical performances akin to those of natural bones^[^ ^640^ ^]^ (Figure 23b). Moreover, the permeability of these scaffolds is also aligned with those of natural bones, thereby facilitating the mass transport of oxygen, nutrients, and wastes. Furthermore, shell lattices, especially TPMS, are widely adopted in biomedical implants for reducing stress concentrations and enhancing the permeability. Recently, hydroxyapatite scaffolds with split‐P TPMS shell lattices were devised, which enhanced the mechanical strength (up to 152 MPa, similar to that of bones) and promoted new bone formation in rabbit femur models.^[^ ^641^ ^]^ In addition, the versatile design of TPMS shell lattices with different Gaussian curvatures was demonstrated, which incorporated biomimetic hyperboloidal topography to direct the osteogenic differentiation and angiogenic paracrine of human mesenchymal stem cells while supporting their attachments and proliferation^[^ ^68^ ^]^ (Figure 23c). The 3D‐printed bioceramic scaffolds significantly enhanced new bone‐tissue growth in both rabbit femoral defects and mouse subcutaneous models, showcasing their potential in bone tissue engineering. Furthermore, using a computational model of neotissue formation, G‐type TPMS shell lattices were optimized to promote bone tissue ingrowth and osteoinduction compared with the granules of Bio‐Oss (a commercial bone substitute material) and SC truss scaffolds^[^ ^642^ ^]^ (Figure 23d).

Moreover, the goal of replicating natural‐tissue properties has also driven the novel design of lattice metamaterials. For example, the incorporation of hierarchical and gradient microarchitectures, which are commonly found in natural materials such as bones, has emerged as a promising strategy for advancing biomedical lattice designs. By combing millimeter‐scaled G‐type TPMS shell lattices with micrometer‐scaled pores on walls, Ti‐6Al‐4V implants with bone‐like mechanical properties and enhanced deformation stability were devised^[^ ^643^ ^]^ (Figure 23e). Drawing inspiration from sea urchin spines, Ti‐6Al‐4V scaffolds with biomimetic tapered struts and graded porosities were devised, which exhibited enhanced mechanical and mass‐transport performances, and superior cellular responses and osteogenic activities.^[^ ^644^ ^]^ These novel designs highlight exciting prospects for the development of bioinspired lattice metamaterials that exhibit superior mechanical and biological performances for biomedical applications. On the other hand, recent advancements in bioprinting techniques and newly developed biomaterials have significantly expanded the medical scenarios.^[^ ^645^ ^]^ For instance, a bioengineered implantable skeletal muscle tissue made of human primary muscle progenitor cells was fabricated via a 3D bioprinting technique^[^ ^67^, ^646^ ^]^ (Figure 23f). A well‐organized multi‐layer muscular bundle composed of viable, closely spaced, and aligned myofiber‐like microarchitectures was visible in bioprinted skeletal muscle tissues. According to the in vivo study, following eight weeks of implantation, the bioprinted muscle constructs were effectively integrated with the host vascular and neural networks and achieved approximately 82% functional recovery in a rodent model of tibialis anterior muscle deficiency. Additionally, by taking advantage of the unique capability of the ruthenium complex and sodium persulfate initiating system to crosslink native tyrosine groups present in non‐chemically modified gelatin, a new class of sacrificial inks that exhibited customizable and programmable delayed dissolution profiles (1–17 days) were developed. By delaying the dissolution of sacrificial inks, this sacrificial bioprinting technique shows the unique ability to control the cell activity, thereby facilitating the deposition of mineralized matrix and the formation of capillary‐like networks in osteogenic and vasculogenic cultures^[^ ^67^, ^646^ ^]^ (Figure 23g). Furthermore, the unique abilities of melt electrowriting are utilized to generate functional scaffolds including precisely regulated fibrous microarchitectures that mimic the wavy microarchitectures of collagen fibers and their recruitment that depends on the load. It opens up a novel way to fabricate scaffolds with precisely regulated microarchitectures, biocompatibility, and tailored nonlinear and anisotropic mechanical properties for use in heart valve tissue engineering^[^ ^634^ ^]^ (Figure 23h).

### 4.6 Other Multifunctional Applications

Owing to the precision in structural control and the capacity of integrating multiple constitutive materials, AM techniques enable simultaneous optimization of mechanical and other physical properties in lattice metamaterials, thereby broadening their applications in many other domains apart from the mainstream multi‐physical applications mentioned above in Sections 4.1–4.5. They include energy harvesters,^[^ ^647^, ^648^, ^649^ ^]^ energy conversion and storage equipment,^[^ ^650^, ^651^, ^652^ ^]^ exchange membranes and efficient catalysts,^[^ ^179^, ^573^ ^]^ morphing aerospace structures,^[^ ^653^ ^]^ self‐sensing and self‐powered equipment,^[^ ^654^, ^655^, ^656^ ^]^ flexible electronic devices,^[^ ^657^, ^658^, ^659^ ^]^ mechanical computing,^[^ ^660^, ^661^ ^]^ human–machine interface,^[^ ^662^, ^663^ ^]^ etc.

**Figure 24:**
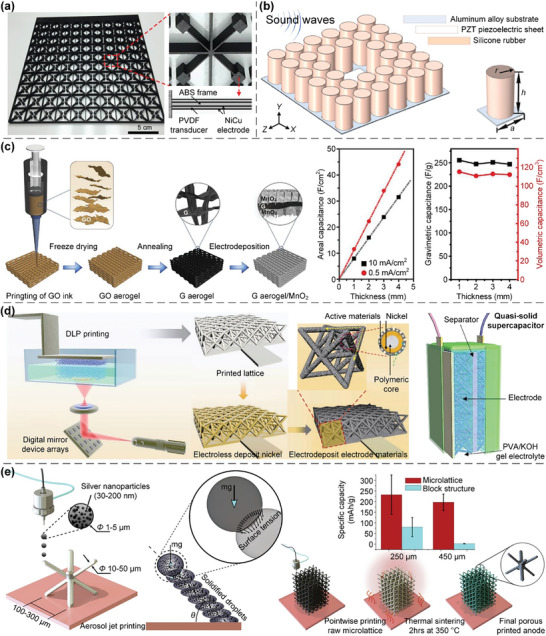
Other multifunctional applications of lattice metamaterials. a) Lattice metamaterials for simultaneous vibration isolation and energy harvesting. Reproduced with permission.^[^ ^647^ ^]^ Copyright 2017, AIP. b) Piezoelectric lattice metamaterials for high‐performance acoustic energy harvesting. Reproduced with permission.^[^ ^648^ ^]^ Copyright 2022, Elsevier. c) 3D‐printed graphene aerogel/MnO_2_ pseudocapacitive electrodes with superior capacitance normalized to area, mass, and volume. Reproduced with permission.^[^ ^534^ ^]^ Copyright 2019, Elsevier. d) 3D‐printed hierarchical composite truss lattices for use as high‐performance quasi‐solid supercapacitors. Reproduced with permission.^[^ ^651^ ^]^ Copyright 2019, Springer. e) Hierarchical porous microlattice electrodes for extremely high capacity lithium ion batteries fabricated by aerosol jet 3D printing. Reproduced with permission.^[^ ^652^ ^]^ Copyright 2018, Elsevier.

More specifically, energy harvesters aim to convert ambient energy, such as vibration, sound, wind, solar, and thermal energy, into electrical energy and to drive low‐power electronic devices. For example, lattice metamaterials that simultaneously achieve energy harvesting and vibration isolation are realized by a square array of free‐standing cantilevers coated with polyvinylidene difluoride (PVDF) that is attached to a 3D‐printed primary structural frame^[^ ^647^ ^]^ (**Figure** **24**a). Thus, a complete bandgap is generated by the distributed local resonance associated with the square array of piezoelectrically active cantilevers and the strong coupling of the bulk elastic wave propagating along the structural frame. The external vibration energy is trapped and converted into the kinetic energy of cantilevers while operating in the stop‐band. Through the mechano‐electrical conversion of integrated piezoelectric elements, the kinetic energy is further transformed into electric energy. Additionally, piezoelectric metamaterials with optimal thickness substrate are proposed to maximize the output power for high‐performance acoustic energy harvesting^[^ ^648^ ^]^ (Figure 24b). Moreover, AM techniques allow for precise control to integrate piezoelectric elements into lattices and can enable potential enhancement of their piezoelectric responses. For example, the effective piezoelectric responses can be significantly enhanced by optimizing AM techniques for miniaturized 3D‐printed piezoceramic microstructural transducers.^[^ ^650^ ^]^ These transducers are designed with specific curvatures and microarchitectures to enable high and localized pressure sensing, thereby enhancing lateral resolution and energy focus.

Lattice metamaterials have also been used to optimize ion diffusion and electrical conductivity in energy storage and electrochemistry, resulting in more efficient and higher‐performance energy conversion and storage equipment. This effect was demonstrated by employing 3D printing and unidirectional freezing to fabricate pseudoplastic nanocomposite metamaterials for intrinsically stretchable supercapacitors.^[^ ^69^ ^]^ The design integrates porous honeycombs with a synergistic blend of materials, leading to a large surface area for energy storage and ion transport with enhanced electrical conductivity and mechanical stability. The unique combination of microarchitecture configurations and constitutive materials results in durable performances, which are capable of enduring substantial deformations without performance degradation. Furthermore, a 3D printable conducting polymer ink based on poly(3,4‐ethylenedioxythiophene):polystyrene sulfonate was introduced into lattice manufacturing to improve the charge storage capacity, long‐term stability, and mechanical flexibility.^[^ ^199^ ^]^ In the realm of energy storage, overcoming the challenges of high mass loading of electrically active materials on electrodes while retaining electrochemical performance is critical.^[^ ^57^ ^]^ Traditional electrodes often suffer from reduced capacitive performance at higher mass loadings as a result of sluggish ion diffusion. Advances in lattice design provide new solutions to this challenge. For instance, a 3D‐printed graphene aerogel pseudocapacitive electrode with an exceptionally high MnO_2_ loading was developed, which exhibits a remarkable areal capacitance^[^ ^534^ ^]^ (Figure 24c). The lattice microarchitectures allow for ultrahigh mass loading of MnO_2_, and facilitates efficient ion diffusion within its porous structures, thus enhancing the capacitive performance. The flexibility in lattice design is also applicable to electrode fabrication, which enlarges electrolyte permeation pathways and interconnected microarchitectures, thereby holding promise for use in the next‐generation high‐energy and high‐power‐density batteries. For example, by combining 3D printing with fast thermal shock technique, an ultrathick electrode of Li‐CO_2_ battery was devised to exhibit low overpotential, high cycling stabilities over 100 cycles, and excellent rate capabilities.^[^ ^70^ ^]^ The design approach, which involves a reduced graphene oxide lattice framework loaded with functional particles, not only facilitates electrolyte permeation and distribution, but also ensures robust electrochemical performances under various operational conditions at the prerequisite of maintaining structural integrity. To overcome the strict rheological requirement of the ink, by utilizing electroless plating and engineering 3D hierarchical porous graphene onto scaffolds, SLA technique is proposed to fabricate hierarchical metallic composite octet‐truss lattices for use in quasi‐solid supercapacitor applications^[^ ^651^ ^]^ (Figure 24d). The supercapacitor devices made of composite truss lattices span several orders of pore size from nm to mm. They show comparable behaviors to the state‐of‐the‐art carbon‐based supercapacitors in terms of areal capacitance (57.75 mF cm^−2^), rate capability (70% retention, 2–40 mA cm^−2^), long lifespan (96% after 5000 cycles), and exceptional energy density (0.008 mWh cm^−2^). Additionally, compared to thin solid Ag block electrodes, microlattice electrodes with porous solid truss components exhibit a remarkable improvement in battery performance, including 400% increase in specific capacity, 100% increase in areal capacity, and a high electrode volume utilization^[^ ^652^ ^]^ (Figure 24e). The microlattice electrodes demonstrate exceptional robustness by retaining morphologies after 40 electrochemical cycles. An exceptionally robust high‐capacity battery system is then generated from 3D microlattices with a hierarchical porosity, which improves electrolyte flow through the electrode volume, expands the surface area for electrochemical reaction, and reduces the intercalation‐induced stress.

In addition, AM techniques have also enabled the incorporation of additional catalytic materials into lattice metamaterials and facilitated their hierarchical designs and surface area optimization for use in catalysts. For instance, a novel 3D freestanding electrode employs a robust corrosion‐resistant Ti‐6Al‐4V titanium alloy lattice as the catalyst support, which is fabricated via LPBF 3D printing technique.^[^ ^573^ ^]^ The intricately designed hierarchical lattices, featuring both microscale and macroscale elements, lead to optimized surface areas and provide efficient pathways for mass and electron transfer, thereby enhancing the overall efficiency of the oxygen evolution reaction process. Moreover, the use of alloy and the specific design of lattice microarchitectures contribute to enhanced durability and stability under harsh conditions that are typically encountered in reaction processes. In summary, these microarchitecture designs highlight the significant role of AM techniques in enhancing the multi‐physical and physicochemical properties of lattice metamaterials. This advancement is pivotal in optimizing the microarchitecture configurations of lattice metamaterials for a broad range of multifunctional applications.

## 5 Future Prospects

After summarizing the existing research outputs of multi‐physical lattice metamaterials enabled by AM techniques in previous sections, here, we attempt to provide our own insights and predictions on the potential future development in this field. We anticipate that the following three fields will grow strongly in future studies of multi‐physical metamaterials, which include the inverse design of novel stimuli‐responsive lattice metamaterials, the design of multi‐physical lattice metamaterials via more freeform design strategies, and the design of novel disordered and quasi‐periodic/aperiodic metamaterials.

### 5.1 Stimuli‐Responsive Lattice Metamaterials

Taking architected metamaterials as black boxes, the innovative stimuli‐responsive mechanisms and the corresponding characterizations deserve more in‐depth studies, including programmable, nonlinear, and large mechanical deformations, multi‐stimuli‐responsive coupling effects, and reprogrammable capabilities^[^ ^109^, ^110^, ^111^, ^112^ ^]^ Unlike conventional lattice metamaterials that maintain their mechanical/physical properties throughout the lifetime, 4D‐printed programmable lattices can sense and respond to external stimuli,^[^ ^664^, ^665^ ^]^ such as sound, electromagnet/light, heat, humidity, and solvent, to achieve tailored multifunctionality. Therefore, they possess the capabilities of mode conversion, shape transformation, and performance regulation in space and time, and serve as a viable approach to achieve a substantially larger design space. However, the development of highly sensitive constitutive materials and the inverse design for 4D printing have been less explored in existing studies on programmable lattice metamaterials. More specifically, existing stimuli‐responsive constitutive materials, such as sound‐sensitive, electromagnet/light‐sensitive, and heat‐sensitive materials, are generally unable to respond in a high speed, and they are typically limited to small deformations and a single deformation mode.^[^ ^666^, ^667^, ^668^, ^669^ ^]^ Accordingly, the reaction speed of these stimuli‐responsive materials needs to be increased in the future studies, and their multi‐mode and large‐strain deformation behaviors need to be further explored. Moreover, existing studies on the inverse design of 4D‐printed programmable lattice metamaterials remain quite limited, and mainly focus on a specific form of internal microarchitectures, such as 2D cellular lattices,^[^ ^585^, ^670^ ^]^ kirigami,^[^ ^671^ ^]^ and 3D truss^[^ ^672^ ^]^ lattices. Consequently, the inverse design of 4D‐printed lattices with more flexible, freeform, and all‐purpose deformation modes warrants more comprehensive studies in the future studies.

### 5.2 Freeform Design Strategies

The development of multi‐physical lattice metamaterials through more freeform design strategies is another emerging topic. More specifically, the two‐scale concurrent design of lattice microarchitectures and their macroscale material distributions via topology optimization serves as a more freeform design approach.^[^ ^157^, ^673^, ^674^, ^675^ ^]^ In the future studies, by incorporating different interacting mechanical/physical properties as the objective and constraint functions, the optimization framework can facilitate the freeform design of lightweight lattices to satisfy multifunctionality requirements. Besides, the AM process requirements of lattice metamaterials, such as connectivity issues and overhang constraints, can be simultaneously addressed in the optimization framework. Furthermore, bioinspired design approaches offer an additional powerful tool for devising multi‐physical lattice metamaterials that simultaneously achieve superior mechanical, biomimetic, and other physical properties.^[^ ^166^, ^676^, ^677^, ^678^ ^]^ Particularly, the creation of biocompatible lattices with superior strength, specific energy absorption, osteogenesis performances, and tailored mechanical‐transport properties, can be successfully accomplished by introducing hierarchical microarchitectures. Moreover, AI techniques serve as another powerful design tool for multi‐physical lattice metamaterials. Traditional design approaches typically encounter expensive computational costs in case of large design scales or complicated physical scenarios.^[^ ^679^, ^680^ ^]^ Recent advances in AI techniques provide an effective way to address this issue by enhancing the design efficiency.^[^ ^681^, ^682^, ^683^, ^684^, ^685^, ^686^ ^]^ In future studies, AI techniques can be widely integrated into the inverse design of lattice metamaterials, in which the load, boundary, and constraint conditions of the design problems need to be parameterized first. Subsequently, the proposed AI models can be trained with sufficient training data comprising optimized designs with different parameters, and then used to predict accurate optimized designs with negligible computational efforts. Alternatively, AI models can also be utilized to predict the mechanical/physical properties of parameterized lattice metamaterials and then integrated into inverse design frameworks to seek optimized designs with target mechanical/physical properties. In addition, following their utilization in the development of lattice metamaterials featuring exceptional properties within a single mechanical/physical field, AI‐enabled design frameworks can be further employed to devise lattice metamaterials with superior multi‐physical properties via multi‐objective optimization. For example, multiple objectives can be taken as mechanical, acoustic, and breathability properties to devise lattice metamaterials with simultaneously enhanced mechanical energy absorption, sound absorption, and breathability, which show great potential in multifunctional applications that demand superior load‐bearing, energy absorption, and mass/heat transfer capabilities. Furthermore, AI techniques can also be integrated into AM processes to develop data‐driven models for expediting data processing and enhancing the manufacturing precision of large‐scale lattice metamaterials.^[^ ^687^, ^688^, ^689^ ^]^ More specifically, AI‐enabled manufacturing techniques can be employed to address tasks such as printing quality assessment, defect detection, and establishing correlations with geometric factors like inclination angles, curvature, feature size, and printing materials. These insights will inform toolpath generation and processing parameter optimization with adaptive configurations tailored to diverse lattice metamaterials encountered in AM processes.

### 5.3 Disordered and Quasi‐Periodic/Aperiodic Metamaterials

**Figure 25:**
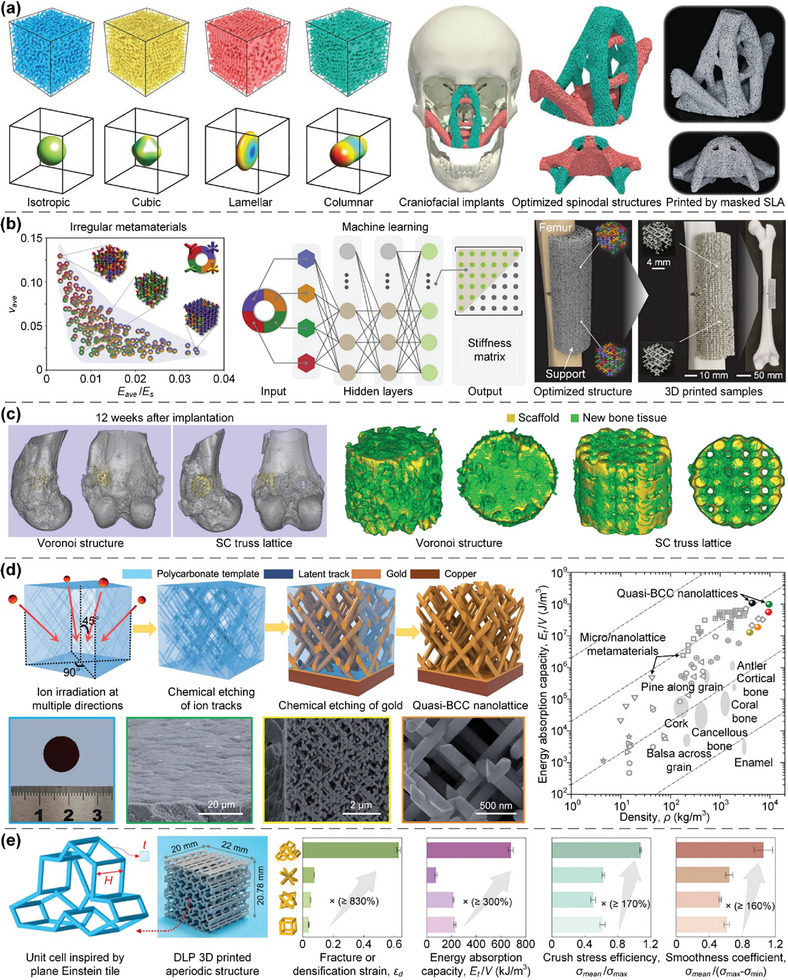
Future Prospects in disordered and quasi‐periodic/aperiodic metamaterials with enhanced multi‐physical properties and functionalities. a) Spinodal metamaterials are bicontinuous, nonperiodic, and stochastic, which promote mechanical and biological functionalities through topology optimization. Reproduced with permission.^[^ ^680^ ^]^ Copyright 2022, Wiley. b) Bioinspired disordered architected metamaterials towards optimal tissue support via topology optimization and machine learning. Reproduced with permission.^[^ ^700^ ^]^ Copyright 2024, Springer. c) In disordered Voronoi metamaterials, a diversified mechanical simulation environment is formed, which involves mass transportation, micro‐deformation, fluid shear stress and pressure, and biological factor diffusion. This environment fits the need for osteogenic mechanical stimulation better, and demonstrates superior osteogenic ability compared to regular scaffolds. Reproduced with permission.^[^ ^699^ ^]^ Copyright 2022, Elsevier. d) The ultrahigh energy absorption capability of quasi‐periodic BCC nanolattices makes them promising candidates for applications in electric conduction, heat transfer, and catalysis. Reproduced with permission.^[^ ^703^ ^]^ Copyright 2023, Springer. e) Aperiodic ordered metamaterials with exceptional strength, toughness, and damage‐tolerance. Reproduced with permission.^[^ ^705^ ^]^ Copyright 2024, Wiley.

As opposed to ordered periodic lattice metamaterials that are intensively reviewed in this article, disordered and quasi‐periodic/aperiodic metamaterials offer a substantially larger design space and merit more thorough studies. Recent developments in AM techniques have also facilitated the manufacture of these architected metamaterials with intricate microarchitectures. More specifically, disordered metamaterials consist of irregularly arranged internal microarchitectures, and can be classified as disordered foams,^[^ ^690^, ^691^, ^692^, ^693^ ^]^ disordered spinodal metamaterials,^[^ ^680^, ^694^, ^695^ ^]^ disordered Voronoi metamaterials,^[^ ^696^, ^697^, ^698^, ^699^ ^]^ and topologically optimized disordered metamaterials.^[^ ^700^, ^701^ ^]^ Disordered metamaterials can be engineered to obtain superior multi‐physical properties, such as bicontinuous, nonperiodic, and stochastic spinodal metamaterials that promote mechanical and biological functionalities^[^ ^680^ ^]^ (**Figure** **25**a), topologically optimized disordered metamaterials that allow for customized stress redistribution^[^ ^700^ ^]^ (Figure 25b), and disordered Voronoi metamaterials that exhibit exceptional osteogenic ability^[^ ^699^ ^]^ (Figure 25c). In addition to disordered metamaterials, the other design forms, such as quasi‐periodic^[^ ^702^, ^703^ ^]^ and aperiodic^[^ ^704^, ^705^, ^706^ ^]^ ordered metamaterials, can also be engineered to obtain superior multi‐physical properties. For instance, quasi‐periodic BCC nanolattices can be tailored to achieve ultrahigh energy absorption capabilities^[^ ^703^ ^]^ (Figure 25d), and aperiodic‐ordered metamaterials can be devised to achieve an unprecedented balance of strength and toughness enhancement^[^ ^704^, ^705^ ^]^ (Figure 25e). In fact, studies on 3D metamaterials initially started with disordered and aperiodic microarchitectures, such as stochastic foams, and later on, ordered periodic lattice metamaterials with simple microarchitectures and superior load‐bearing capabilities emerged. To date, disordered and quasi‐periodic/aperiodic metamaterials are found to exhibit more exceptional performances than ordered periodic lattices in terms of stiffness,^[^ ^702^ ^]^ strength,^[^ ^704^, ^705^ ^]^ toughness,^[^ ^704^, ^705^ ^]^ specific energy absorption,^[^ ^703^ ^]^ and bone regeneration capabilities.^[^ ^680^, ^699^, ^700^ ^]^ The reason is primarily attributed to the specific fragile zones or weak directions of ordered periodic lattices, while this issue can be efficiently overcome by the randomness of disordered and aperiodic metamaterials. Typically, disordered and quasi‐periodic/aperiodic‐ordered metamaterials possess a substantially larger number of design variables, thus necessitating a much higher computational cost in the design process. In future studies, AI‐enabled inverse design frameworks can be integrated into their design processes to enhance the design efficiency while maintaining superior multi‐physical properties. Besides, AI‐enabled 3D printing techniques can also be utilized to enhance the fabrication efficiency of disordered and quasi‐periodic/aperiodic metamaterials. More importantly, in pursuit of extreme multi‐physical properties and multifunctionalities, the optimized microarchitectures obtained by topology optimization and AI algorithms will most likely be disordered and aperiodic. The reason is that most application scenarios do not involve a regular block, and the irregular boundary conditions and multi‐parameter optimization algorithms tend to result in disordered and aperiodic microarchitectures. Overall, disordered and aperiodic metamaterials possess a substantially larger design space, exhibit more exceptional multi‐physical properties and multifunctionalities, and can be efficiently devised and fabricated via AI‐enabled design and AM processes. As a result, disordered and aperiodic metamaterials may gain more popularity in future studies.

### 5.4 Brief Prospect Summary

Overall, we have summarized the challenges and future prospects of multi‐physical metamaterials in three aforementioned fields, namely the inverse design of 4D‐printed stimuli‐responsive lattice metamaterials, the design of multi‐physical lattice metamaterials via more freeform design strategies (including topology optimization and AI algorithms), and the design of novel disordered and quasi‐periodic/aperiodic metamaterials. By addressing challenges pertaining to these fields, we anticipate an increasing number of innovative AM techniques‐enabled multi‐physical metamaterials to be devised for a broader range of multifunctional applications. More significantly, by overturning the cognitive limitations of humans on the physical properties and apparent natural laws of traditional materials, the concept of lattice metamaterials will serve as a powerful means to explore new material properties and functionalities, simultaneously enhance the multifunctional performances of architected materials, which will lead to new industrial applications and new areas for economic growth.

Moreover, although recent advances in AM techniques have facilitated the fabrication of lattice metamaterials with highly intricate internal microarchitectures, there still remain some technical challenges.^[^ ^707^ ^]^ For instance, the overhang constraints, surface roughnesses, and dimensional inaccuracies, especially at small scales, present significant challenges in producing high‐fidelity lattice metamaterials. Another significant challenge involves the validation of theoretical models used to predict the behaviors of multi‐physical lattice metamaterials. Although numerical simulations provide valuable insights into the expected performances of these architected materials, the real‐world testing often reveals discrepancies between the predicted and actual performances due to imperfections introduced during the manufacturing processes. The control of defects and the evaluation of their effects are key issues in the manufacturing processes, which, when combined with printing constraints, in turn affect the design rules of multi‐physical lattice metamaterials.^[^ ^151^, ^708^ ^]^ Besides, the interactions between various physical properties (mechanical, acoustic, EM/optical, thermal, and other properties) in lattice metamaterials typically require the use of multi‐material systems. The different constitutive materials, which are characterized by different mechanical/physical properties, usually require distinct manufacturing environments and conditions. However, current AM techniques still face limitations in terms of the number of materials that can be simultaneously fabricated,^[^ ^709^, ^710^, ^711^, ^712^ ^]^ and the mechanical/physical properties of the fabricated materials may degrade over time due to environmental factors such as temperature and humidity. Furthermore, another critical challenge lies in the scalability of AM techniques for large‐scale industrial applications. Although AM techniques exhibit significant advantages for prototyping and small‐batch production, the scalability and manufacturing speed of lattice metamaterials for mass production remains an challenge.^[^ ^713^ ^]^ These technical challenges need to be addressed via further exploration and development of AM technologies in the future studies.

## 6 Conclusions

In this review, we provide a comprehensive summary of the design principles, structure‐mechanism‐property relationships, interaction mechanisms, and multifunctional applications of lattice metamaterials enabled by AM techniques. First, the design principles and AM procedures of homogeneous lattices, inhomogeneous lattices, and other forms of lattice metamaterials are reviewed, in which the benefits and drawbacks of utilizing different AM techniques to fabricate different types of lattice metamaterials are discussed. Subsequently, the correlations between the internal microarchitectures and mechanical/physical properties of lattice metamaterials are summarized. In addition, we point out that tailoring lattice microarchitectures and selecting appropriate constitutive materials can enable the formation of lattices with a wide range of unconventional mechanical/physical properties, including acoustic, EM/optical, thermal, and many other physical properties. Moreover, the multifunctional applications of lattice metamaterials in various fields, including sound absorbers, insulators, and manipulators, sensors, actuators, and soft robots, thermal management, invisible cloaks, and biomedical implants, are enumerated. Specifically, all of the reviewed applications involve the coupling of properties from two different or even more disciplines: typically, a mechanical property and another physical property at least. Finally, a detailed discussion on the challenges and future prospects regarding the expanded design principles, unexplored multi‐physical properties, and potential multifunctional applications of lattice metamaterials enabled by AM techniques are discussed. Overall, this review provides the first comprehensive summary of AM techniques‐enabled multi‐physical lattice metamaterials, with a clear classification of their categories and respective design guidelines. Moreover, multiple classes of application scenarios are presented to support the design and development of novel lattice metamaterials with exceptional multifunctionalities.

## Conflict of Interest

The authors declare no conflict of interest.
